# Functional Classification of Uncultured “*Candidatus* Caldiarchaeum subterraneum” Using the Maple System

**DOI:** 10.1371/journal.pone.0132994

**Published:** 2015-07-21

**Authors:** Hideto Takami, Wataru Arai, Kazuhiro Takemoto, Ikuo Uchiyama, Takeaki Taniguchi

**Affiliations:** 1 Microbial Genome Research Group, Yokohama Institute, Japan Agency for Marine-Earth Science and Technology (JAMSTEC), Yokohama, Kanagawa, Japan; 2 Department of Bioscience and Bioinformatics, Kyushu Institute of Technology, Iizuka, Fukuoka, Japan; 3 Laboratory of Genome Informatics, National Institute for Basic Biology, National Institutes of Natural Sciences, Nishigonaka, Myodaiji, Okazaki, Aichi, Japan; 4 Graduate School of Bioscience and Biotechnology, Tokyo Institute of Technology, Meguro-ku, Tokyo, Japan; 5 Advanced Science & Innovation Group, Mitsubishi Research Institute Inc. (MRI), Nagata-cho, Chiyoda-ku, Tokyo, Japan; University of Freiburg, GERMANY

## Abstract

In this study, the metabolic and physiological potential evaluator system based on Kyoto Encyclopedia of Genes and Genomes (KEGG) functional modules was employed to establish a functional classification of archaeal species and to determine the comprehensive functions (functionome) of the previously uncultivated thermophile “*Candidatus* Caldiarchaeum subterraneum” (*Ca*. C. subterraneum). A phylogenetic analysis based on the concatenated sequences of proteins common among 142 archaea and 2 bacteria, and among 137 archaea and 13 unicellular eukaryotes suggested that *Ca*. C. subterraneum is closely related to thaumarchaeotic species. Consistent with the results of the phylogenetic analysis, clustering and principal component analyses based on the completion ratio patterns for all KEGG modules in 79 archaeal species suggested that the overall metabolic and physiological potential of *Ca*. C. subterraneum is similar to that of thaumarchaeotic species. However, *Ca*. C. subterraneum possessed almost no genes in the modules required for nitrification and the hydroxypropionate–hydroxybutyrate cycle for carbon fixation, unlike thaumarchaeotic species. However, it possessed all genes in the modules required for central carbohydrate metabolism, such as glycolysis, pyruvate oxidation, the tricarboxylic acid (TCA) cycle, and the glyoxylate cycle, as well as multiple sets of sugar and branched chain amino acid ABC transporters. These metabolic and physiological features appear to support the predominantly aerobic character of *Ca*. C. subterraneum, which lives in a subsurface thermophilic microbial mat community with a heterotrophic lifestyle.

## Introduction

Since the existence of undescribed physiological types of archaea in addition to extremophiles and methanogens in marine plankton was demonstrated in 1992 [1, 2], subsequent studies have characterized the archaeal abundance and diversity in the overall biosphere, suggesting that archaeal species have significant impacts on carbon and nitrogen cycling [3]. In addition to traditional cultivation methods, culture-independent metagenomic analyses, particularly those based on high-throughput sequencing, are now feasible methods to obtain genomic sequences of uncultivated archaea that thrive in various environments [4, 5]. In fact, the complete genomes of uncultivated archaea, including those within candidate phyla, have been recovered solely from metagenomic sequences [6–8]. Another culture-independent approach to identify genomes from uncultivated microbes is single-cell genomics, which involves the amplification and sequencing of DNA from single cells collected directly from terrestrial and marine environmental samples [9–12].

We obtained the complete genome of an uncultivated archaeal species in the candidate phylum, Hot Water Crenarchaeotic Group I, from the metagenomes of a microbial mat community that flourishes along a subsurface geothermal stream [13–15]. The phylogenetic position of the microbe based on this composite genome was similar to that of thaumarchaeotic species such as *Nitrosopumilus maritimus* and *Cenarchaeum symbiosum*. However, we proposed that this crenarchaeotic group should be considered a novel phylum, *Aigarchaeota*, owing to its unique genomic traits that were distinct from those of known phyla and candidate divisions such as *Crenarchaeota*, *Euryarchaeota*, “Korarchaeota”, *Nanoarchaeota*, and *Thaumarchaeota*; accordingly, we named the microbe *Candidatus* Caldiarchaeum subterraneum (*Ca*. C. subterraneum) [14].

A primary objective of genomic analyses of uncultivated *Ca*. C. subterraneum is to deduce its lifestyle on the basis of potential comprehensive functions (functionome) of genes harbored in its genome. We performed a detailed genomic analysis of its metabolic and physiological features, but it was difficult to capture its overall functional potential and to compare it with that of other archaea. The functional categories defined by the Kyoto Encyclopedia of Genes and Genomes (KEGG) [16] and SEED [17] databases are extremely broad with respect to metabolic and physiological features. Additionally, it is difficult to automatically annotate each function solely by assigning ortholog identifiers (IDs). However, KEGG provides an easy method for the computational annotation and characterization of more detailed individual functions using four types of functional modules: pathway, molecular complex, functional set, and signature modules [18]. These modules comprise small units of subpathways and multiple molecules such as the subunits of transporters and receptors, which encompass broad functional categories, including energy metabolism, carbohydrate and lipid metabolism, and nucleotide and amino acid metabolism. Previously, we developed a method to evaluate the potential functionome by calculating the module completion ratios (MCRs) for KEGG modules [19]. We also developed a metabolic and physiological potential evaluator (MAPLE) to automate a series of steps used in this method [20]. In the present study, we applied the MAPLE system (http://www.genome.jp/) to the functional classification of archaea and characterized the potential functionome of the *Ca*. C. subterraneum genome.

In this study, we performed clustering and statistical analyses to determine functional classifications based on the MCR patterns of 79 archaeal species, including the uncultivated *Ca*. C. subterraneum. We also determined that the metabolic potential of *Ca*. C. sunterraneum based on overall MCR patters was similar to that of thaumarchaeotic species.

## Materials and Methods

### Phylogenetic analysis based on common orthologous genes

The orthologous relationships between each *Ca*. C. subterraneum gene and the genes of other prokaryotic species were identified using the DomClust program [21] based on the all-against-all similarities between *Ca*. C. subterraneum and 143 prokaryotic species (141 archaeal species and two bacterial species, as described in S1 Table) from the MBGD database [22]. Similarly, the orthologous relationships between *Ca*. C. subterraneum and 149 other organisms (136 archaeal species and 13 unicellular eukaryotic species, as listed in S1 Table) were identified. The resulting ortholog table was visualized and analyzed using the RECOG system (http://mbgd.genome.ad.jp/RECOG/). To determine the phylogenetic position of *Ca*. C. subterraneum among archaeal species, phylogenetic analyses were performed using a concatenated alignment of single-copy protein-coding genes without domain splitting, which were selected using the MBGD database. Twenty-two orthologous families conserved in 142 archaea and two bacteria, or 12 orthologous families conserved in 137 archaea and 13 unicellular eukaryotes were used for each phylogenetic analysis. The sequences of genes in these families were aligned using MUSCLE [23] with the parameter "—maxiterate 1000" and the resulting alignments were processed using the Gblocks program [24] with the default settings to eliminate poorly aligned positions. The processed alignments were concatenated and subjected to a phylogenetic analysis. The phylogenetic position of *Ca*. C. subterraneum was inferred by MEGA6 using the maximum-likelihood method [25]. The substitution model LG+ had the lowest Akaike information criterion (or, AIC) value and was selected as the best-fit model by ProtTest 2.4 [26]. According to this result, the LG+G model was employed to produce the maximum-likelihood tree (1,000 bootstrap replicates using the LG model with gamma distributed rates, partial deletion of gaps, and a site coverage cutoff of 95%).

### Calculation of the MCR and pathway analysis

MCRs of all KEGG functional modules (228 pathways, 271 molecular complexes, 86 functional sets, and 8 signature modules) in each type of archaea were calculated based on a Boolean algebra-like equation [19] using the MAPLE system [20]. MAPLE is an automatic system that is used to map genes in an individual genome and metagenome to functional modules defined by KEGG. It calculates the MCR, the percentage of a module component filled with input genes, as follows: it assigns a KEGG orthology (KO) ID to the query genes using the KEGG Automatic Annotation Server (KAAS), maps the KO-assigned genes to the KEGG functional modules, and finally calculates an MCR for each functional module. If all genes are assigned to all KO IDs in each reaction according to the Boolean algebra-like equation, the MCR is 100%. For this analysis, one genome was selected from each archaeal genus (a total of 79 genomes, including *Ca*. C. subterraneum) listed in S2 Table and a reference genome set was constructed to cover all of the completely sequenced prokaryotes (as of August 2, 2014). When the type species for each genus was registered in the KEGG GENOME database, it was selected as a representative in this study; if not, the first registered species for each genus was selected as a representative. If there were multiple genomic sequences for a single species, the earliest registered sequence was selected. The KEGG pathway [27] and module databases were used for pathway construction [18].

### Clustering and PCA analyses based on the MCR pattern

To characterize the overall MCR pattern of *Ca*. C. subterraneum based on comparisons with those of the other 78 archaea, the pairwise Euclidean distances between the overall MCR patterns for each archaea were used and the complete-linkage clustering method was employed for the functional classification of 79 archaea using an R statistical package [28]. KEGG modules with the same MCR values ranging from 0% to 100% in all archaea, including *Ca*. C. subterraneum, were excluded from this analysis. Similar to the clustering analysis, the overall MCR patterns of *Ca*. C. subterraneum and the other 78 archaea, except for the modules with no differences in the MCR values among all archaea, were subjected to a principal components analysis (PCA). PCA was performed using the R statistical environment.

## Results

### Phylogenetic position of *Ca*. C. subterraneum among archaea

**Fig 1:**
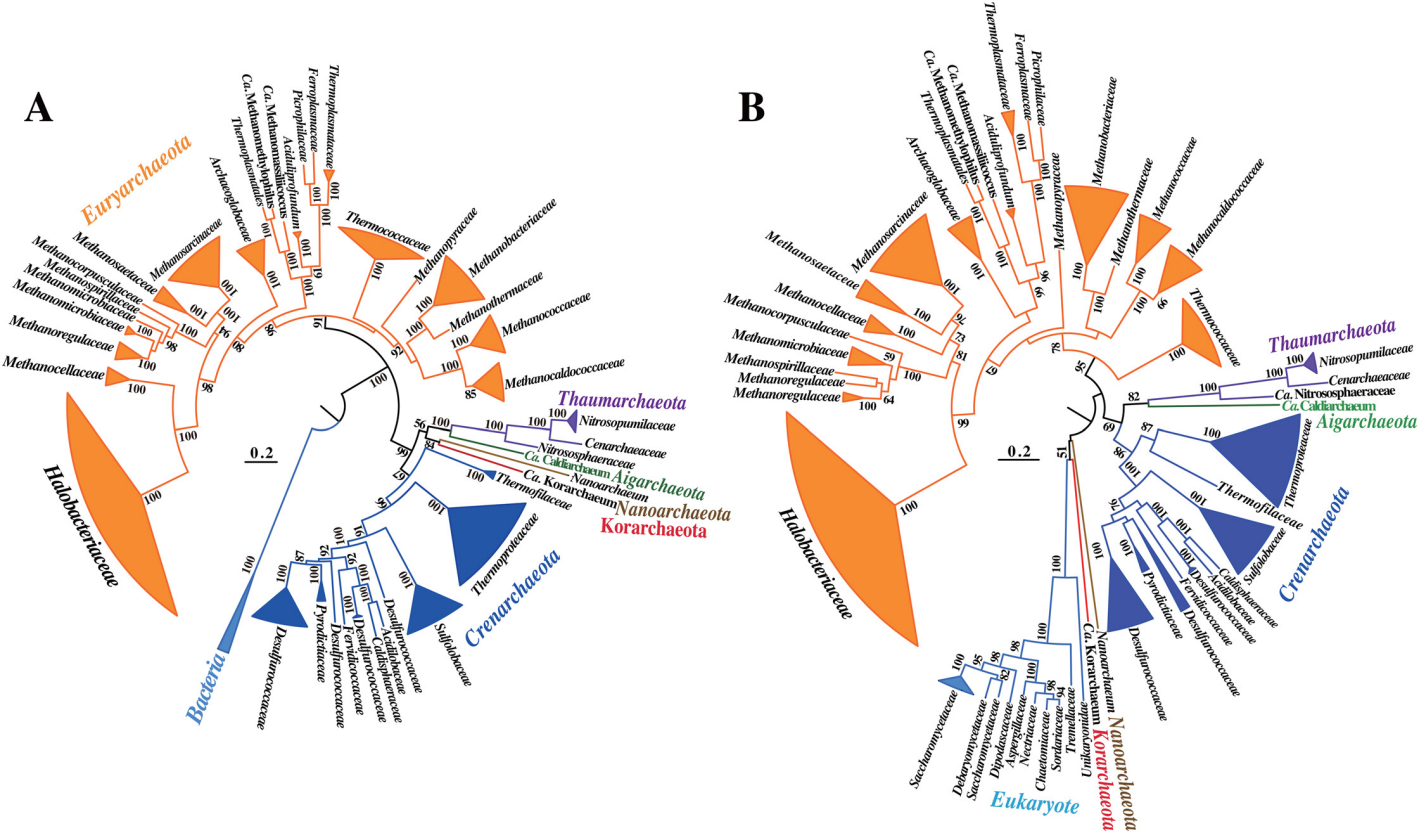
Phylogenetic position of “*Candidatus* Caldiarchaeum subterraneum” based on concatenated common protein sequences. A maximum-likelihood tree was constructed using MEGA [25]. The phylogenetic trees were collapsed at the family level except for *Nanoarchaeum*, “*Ca*. Korarchaeum,” and “*Ca*. Caldiarchaeum.” (A) Concatenated alignment of the sequences of 22 common proteins with no paralogs and domain splitting (hisS, pheS, valS, rpl11p, rpl14p, rpl1P, rpl22p, rpl2p, rpl3p, rpl5p, rpl6p, rplW, rps10p, rps11p, rps19p, rps2P, rps3p, rps4p, rps5p, rps7p, rps8p, and rps9p) among 142 archaea, including “*Ca*. A. autotrophicum” and two bacteria as an outgroup, as listed in S1 Table. (B) Concatenated alignment of the sequences of 12 common protein sequences without paralogs and domain splitting (eif2G, rfcL, eif6, rpl10e, rpl15p, rpl18p, rpl7ae, rplP0, rps19p, rps3p, rps5p, and rio1) among 137 archaea, including “*Ca*. C. subterraneum” and 13 unicellular eukaryotes as the outgroup (S1 Table). The numbers indicate the bootstrap support expressed as percentages. Bootstrap values of less than 50% were omitted.

To confirm the phylogenetic position of the thermophile *Ca*. C. subterraneum among archaea, we conducted a genome-wide phylogenetic analysis using the maximum-likelihood method with the LG+G substitution model, which was not used in the previous study [14] and we obtained a concatenated alignment of 22 broadly conserved proteins among 79 archaea, including *Ca*. C. subterraneum and two representative bacterial species (*Escherichia coli* and *Bacillus subtilis*) as an outgroup (S1 Table). The phylogenetic tree demonstrated that *Ca*. C. subterraneum formed a cluster with five species within *Thaumarchaeota* (Fig 1A). The thermophile “*Candidatus* Nitrososphaera gargensis” (*Ca*. N. gargensis) [29, 30] was somewhat distantly related to other mesophilic thaumarchaeotic species; species within the families *Nitrosopumilaceae* and *Cenoarchaeaceae* comprised a sub-cluster. Thus, *Ca*. C. subterraneum was closely related to the thermophile *Ca*. N. gargensis based on this tree (Fig 1A). We constructed another phylogenetic tree using the concatenated alignment of 12 broadly conserved genes among 137 archaea, including *Ca*. C. subterraneum, and 13 unicellular eukaryotes as the outgroup. The topology was similar to that of the former tree except for the phylogenetic position of the species within “Korarchaeota” and *Nanoarchaeota*, but there was no difference in the phylogenetic relationships between *Ca*. C. subterraneum and thaumarchaeotic species (Fig 1B). “Korarchaeota” formed a cluster with the eukaryotic cluster, which comprised several sub-clusters, but *Nanoarchaeota* was independent in this tree, although these two species formed a cluster in the previous tree.

### Functional classification of archaea based on the MCR patterns of the KEGG modules

**Fig 2:**
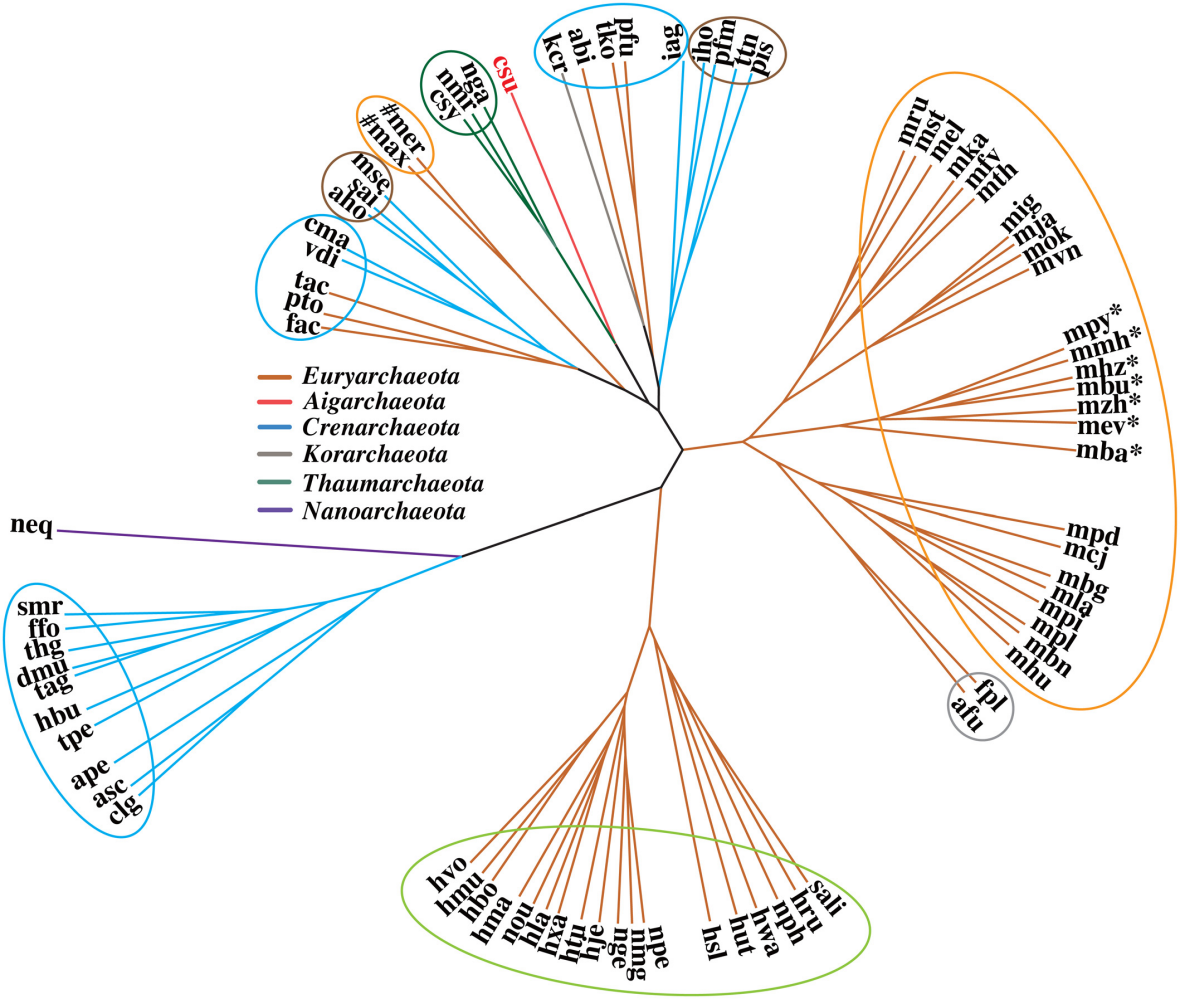
Functional classification of archaea based on the MCR patterns of the KEGG modules. csu, *Ca*. Caldiarchaeum subterraneum; nga, *Ca*. Nitrososphaera gargensis; nmr, *Nitrosopumilus maritimus*; csy, *Cenarchaeum symbiosum*. Full species names for all other abbreviations are listed in S2 Table. Orange circle, methanogens; yellow-green circle, halophiles; gray circle, hyperthermophilic facultative autotrophic sulfur reducers; blue circle, heterotrophic (hyper)-thermophilic acidophiles or neutrophiles; brown circle, chemolithoautotrophic or facultative autotrophic (hyper)-thermophilic acidophiles; green circle, facultative autotrophic ammonia oxidizers. *, methanogens, which can generate methane from all possible substrates (CO_2_, acetate, methanol, and methylamine); #, methanogens isolated from the human gut, which can generate methane only from methanol.

The MCR of all KEGG modules in 79 archaea, including *Ca*. C. subterraneum, was calculated to evaluate their metabolic and physiological potential using the MAPLE system [20] (S1 Fig and S3 Table). The MCR patterns of 273 modules, except those with identical MCR values, among 79 archaea were subjected to a clustering analysis to obtain functional classifications of archaea. Three thaumarchaeotic species formed a small cluster with *Ca*. C. subterraneum, suggesting similar overall metabolic and physiological potential (Fig 2). Thaumarchaeotic species have been known as chemolithoautotrophic ammonia oxidizers, but recent marine isolates, *Nitrosopumilus maritimus* strains PS0 and HCA1, display obligate mixotrophy [31]. Thus, *Ca*. C. subterraneum is similar to thaumarchaeotic species with respect to not only phylogeny, but also metabolic and physiological potential. Species within *Euryarchaeota* were divided into two major clusters, i.e., halophiles and methanogens, and small clusters such as hyperthermophilic sulfur reducers and hyperthermophilic acidophiles or neutrophiles. The methanogens isolated from environmental samples such as terrestrial soils and a hydrothermal vent formed a large cluster with three sub-clusters. However, interestingly, two other methanogens, “*Ca*. Methanomassiliicoccus intestinalis” [32] and “*Ca*. Methanomethylophilus alvus” [33], isolated from human feces, formed an independent cluster, although the two species were distantly related based on the phylogeny (Fig 2). This result indicates that the difference in habitat clearly reflects the overall difference in metabolic and physiological potential, whereas methanogenesis is a common phenotypic property. Both species are only able to generate methane from methanol, while all other species can generate methane from CO_2_. In addition, seven species that formed another cluster, *Methanosarcina barkeri*, *Methanococcoides burtonii*, *Methanohalophilus mahii*, *Methanohalobium evestigatum*, *Methanosalsum zhilinae*, *Methanolobus psychrophilus*, and *Methanomethylovorans hollandica*, have the ability to generate methane from all 4 possible substrates, CO_2_, acetate, methanol, and methylamine (Fig 2). Species within *Crenarchaeota* were divided into three clusters and a major cluster comprised of heterotrophic (hyper)-thermophilic acidophiles or neutrophiles. The members that formed this cluster were sulfur compound reducers, except *Fervidicoccus fontis* [34] and *Thermosphaera aggregans* [35]. Three other crenarchaeotic species with similar phenotypic properties to the members that formed the major cluster were spread between two different clusters comprising chemolithoautotrophic or mixotrophic (hyper)-thermophilic acidophiles.

### PCA of the MCR patterns

**Fig 3:**
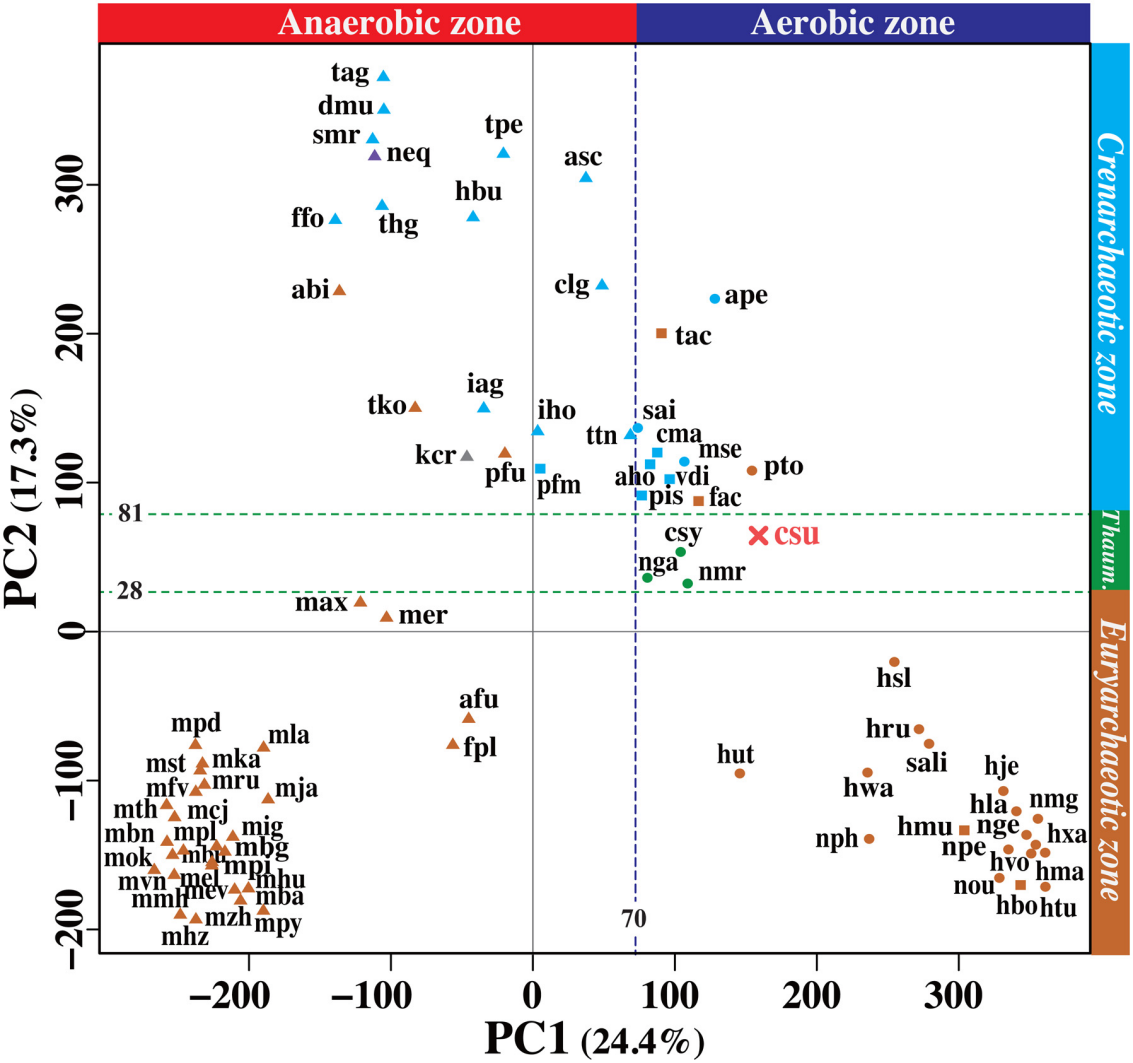
Distribution of “*Ca*. C. subterraneum” along the first and second PCA axes based on MCR patterns. Obligate and facultative anaerobes are denoted by triangles and squares, respectively. Aerobes are denoted by circles. X indicates uncultivated species. Light brown, *Euryarchaeota*; light blue, *Crenarchaeota*; green, *Thaumarchaeota*; gray, “Korarchaeota”; purple, *Nanoarchaeota*; csu, “*Ca*. Caldiarchaeum subterraneum”. Full species names for all other abbreviations are listed in S2 Table. MCR patterns of 273 modules (pathway: 161, structural complex: 101, and functional set: 11), except for those with the same MCR values, among 79 archaea were subjected to this analysis.

Hierarchical clustering (Fig 2) provides an intuitive means to visualize a classification; however, it cannot explain the dominant factors (i.e., reaction modules, in this study) that determine the clusters owing to limitations of the method. Thus, we performed a PCA, which explains the main factors determining the clusters by avoiding problems that result from multicollinearity (i.e., overlap among modules). The overall physiological features that characterize aerobes and anaerobes were identified according to the preliminary PCA of the MCR patterns of the archaeal species. In this analysis, the first principal component (PC1) was correlated with the oxygen requirement for growth, so that anaerobes and aerobes could be distinguished along the first axis. The second PC (PC2) was correlated with the phylogenetic classification at the phylum level, such as *Crenarchaeota*, *Thaumarchaeota*, and *Euryarchaeota*. Thus, we used the PCA to confirm whether *Ca*. C. subterraneum possessed a signature similar to that of other aerobes as well as phylogenetically related species. As shown in Fig 3, all aerobic archaeal species, including facultative anaerobes, were located to the right-hand side of the border (ca. 70), which discriminated aerobes from anaerobes, except for *Pyrolobus fumarii* [36], a facultative microaerophile. *Ca*. C. subterraneum was positioned at the right-hand side of the border along with the aerobes *Picrophilus torridus* [37], *Aeropyrum pernix* [38], and *Halorhabdus utahensis* [39]. KEGG modules such as methanogenesis (M00356), pyruvate:ferredoxin oxidoreductase (M00310), succinate dehydrogenase (M00149), and cytochrome c oxidase (M00155) contributed to the variance along the first principal axis, although the factor loading of each module was small because the analysis included over 270 modules (S2A Fig).

Most of the euryarchaeotic species were located in a euryarchaeotic zone along the second axis, except for several (hyper)-thermophilic heterotrophs. These exceptional euryarchaeotic species were mixed within the crenarchaeotic zone, together with *Nanoarchaeum equitans* [40] and “*Ca*. Korarchaeum cryptofilum” [9]. Consequently, *Ca*. C. subterraneum was located in the thaumarchaeotic zone (ca. 28–81). In this case, modules, such as the archaeal exosome (M00390), which was identified in archaea, except for the halophiles and half of the methanogens, and DNA polymerase II complex (M00261), were not identified in *Crenarchaeota* and contributed to the variance in PC2, as shown in S2B Fig. These results demonstrate that *Ca*. C. subterraneum is probably an aerobe that is similar to thaumarchaeotic species in overall metabolic and physiological potential; however, more careful examinations of these properties are required owing to the low percentages of variance explained (see Discussion).

### MCR patterns involved in carbon fixation

**Fig 4:**
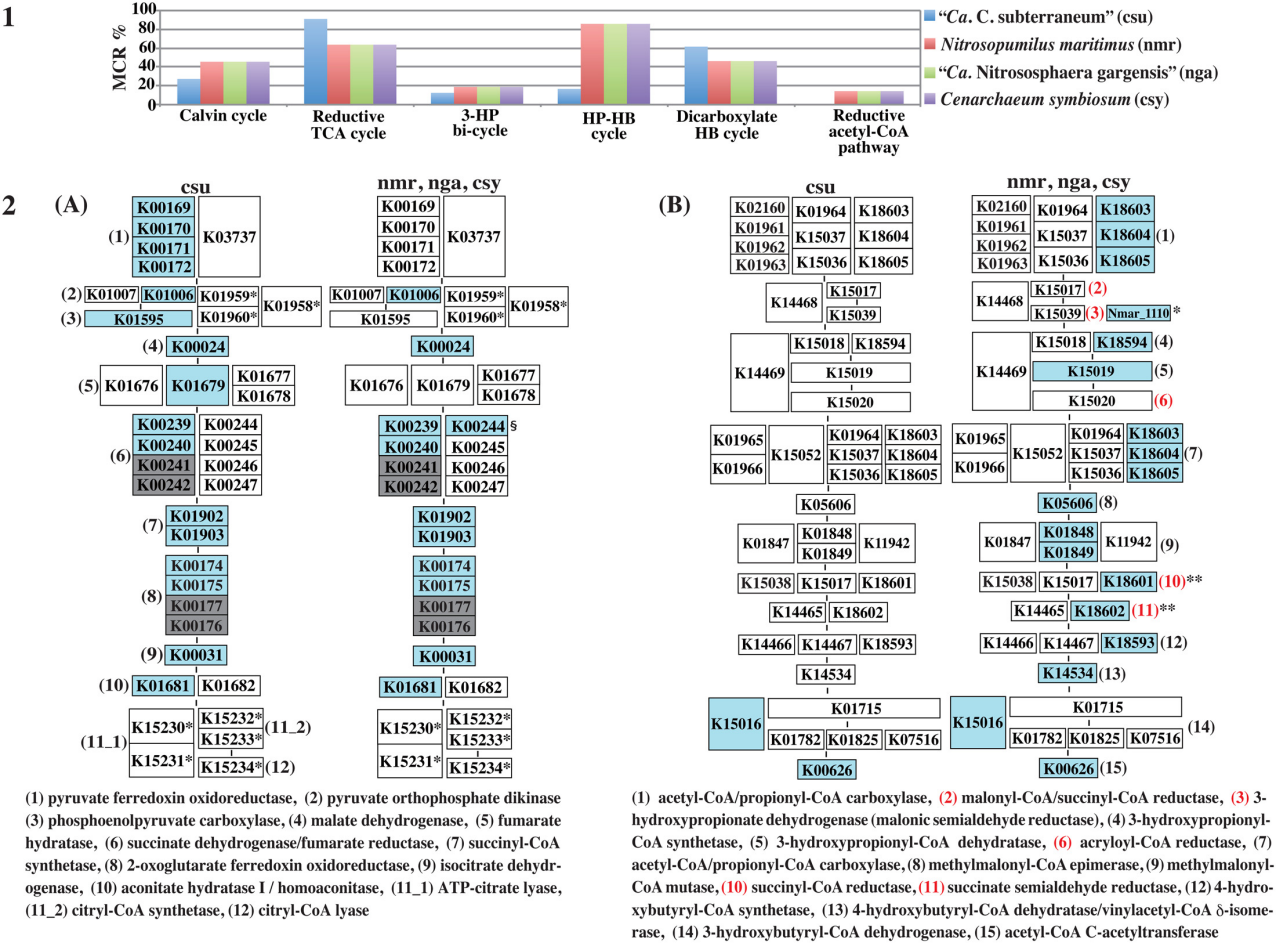
Comparison of MCR patterns for carbon fixation in “*Ca*. C. subterraneum” and three thaumarchaeotic species. **1.** MCR patterns of six major carbon fixation pathways. 3–HP bicycle, 3–hydroxypropionate bicycle; HP–HB cycle, hydroxypropionate–hydroxybutyrate cycle; dicarboxylate HB cycle, dicarboxylate hydroxybutyrate cycle; **2,** Mapping patterns of genes to two carbon fixation modules. Numbers in parentheses show the order of reaction steps in each module. In each “K number” set of components of the module, the vertically connected and horizontally located K numbers indicate complexes and alternatives, respectively [19]. **(A)** Reductive TCA cycle (M00173). *Specific enzymes for this pathway; §found only in *Cenarchaeum symbiosum*. **(B)** HP–HB cycle (M00375). Numbers in red show the reaction steps for which the corresponding genes were not identified in the genomes of the three thaumarchaeotic species, although their enzymatic activities have been detected experimentally [44]. *The gene responsible for step 3 has been experimentally identified in the *N*. *maritimus* genome very recently, whereas the K number has not yet been assigned to the gene (Nmar_1110) by KEGG [48]. **Because the K numbers were incorrectly assigned to the genes of thaumarchaeotic species, the genes responsible for steps 10 and 11 have not yet been identified in their genomes.

**Fig 5:**
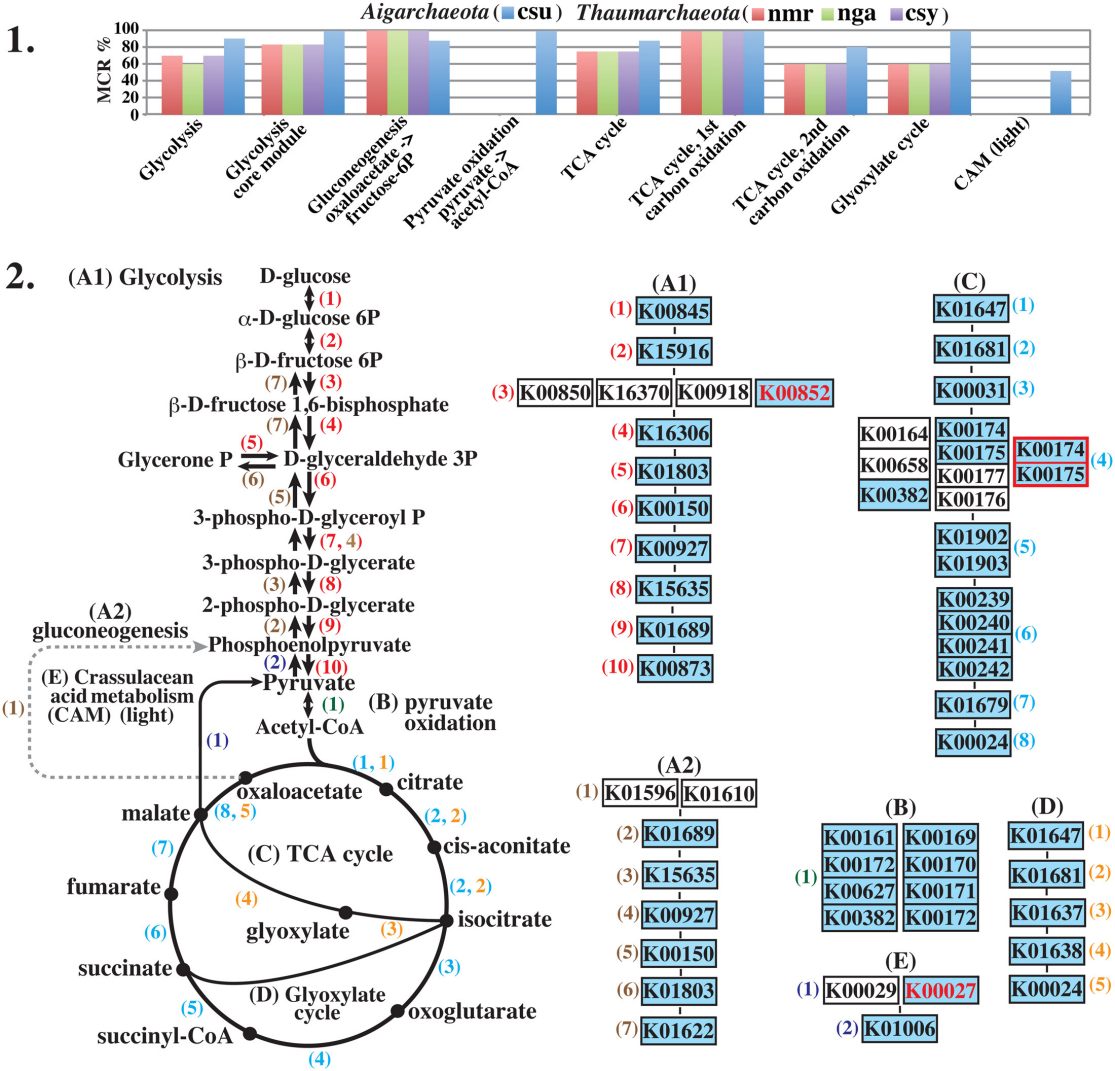
Major central carbohydrate metabolism in “*Ca*. C. subterraneum.” **1.** Comparison of the MCR patterns between aigarchaeotic and thaumarchaeotic species. csu, *Ca*. Caldiarchaeum subterraneum; nmr, *Nitrosopumilus maritimus*; nga, *Ca*. Nitrososphaera gargensis; csy, *Cenarchaeum symbiosum*. **2.** Pathway map for central carbohydrate metabolism (left side) and mapping pattern of the modules corresponding to the pathway map in *Ca*. C. subterraneum (right side). In each “K number” set of components of the module, the vertically connected and horizontally located K numbers indicate complexes and alternatives, respectively [19]. K numbers in blue boxes indicate the orthologous genes identified in *Ca*. C. subterraneum. Grayish dashed lines and arrows show missing reaction steps in the pathway. Numbers in parentheses on each module component correspond to those on the pathway map. A1, glycolysis (M0001): (1), glucokinase; (2), glucose/mannose-6-phosphate isomerase; (3), 6-phosphofructokinase or ADP-dependent phosphofructokinase/glucokinase; (4), fructose-bisphosphate aldolase; (5), triosephosphate isomerase; (6), glyceraldehyde-3-phosphate dehydrogenase; (7), phosphoglycerate kinase; (8), 2,3-bisphosphoglycerate-independent phosphoglycerate mutase; (9), enolase; (10), pyruvate kinase. A2, gluconeogenesis (M0003): (1), ADP-dependent phosphofructokinase/glucokinase; (2), enolase; (3), 2,3-bisphosphoglycerate- independent phosphoglycerate mutase; (4), phosphoglycerate kinase; (5), glyceraldehyde-3-phosphate dehydrogenase, (6), triosephosphate isomerase; (7), fructose 1,6-bisphosphate aldolase/phosphatase. B, pyruvate oxidation (M00307): (1), pyruvate dehydrogenase. C, TCA (Krebs) cycle (M0009): (1), citrate synthase; (2), aconitate hydratase; (3), isocitrate dehydrogenase; (4), 2-oxoglutarate dehydrogenase; (5), succinyl-CoA synthetase; (6), succinate dehydrogenase; (7), fumarate hydratase, class II; (8), malate dehydrogenase. D, glyoxylate cycle (M00012): (1), citrate synthase; (2), aconitate hydratase; (3), isocitrate lyase; (4), malate synthase; (5), malate dehydrogenase. M number shows identifier of each KEGG module. E, Crassulacean acid metabolism–light (M00169): (1), malate dehydrogenase; (2), pyruvate, orthophosphate dikinase. K numbers shown in red have not been assigned to the KEGG module, but similar enzymatic reactions to the K numbers assigned to the module have been confirmed. The reaction component shown by the red frame in the TCA cycle shows αβ-heterodimeric 2-oxoacid:ferredoxin oxidoreductase [55, 56], which is not yet reflected in the KEGG module.

The results of our analyses suggested that *Ca*. C. subterraneum is similar to thaumarchaeotic species, both phylogenetically and in terms of its overall metabolic and physiological potential. Thus, we focused on the MCR patterns of six types of carbon fixation modules in *Ca*. C. subterraneum and thaumarchaeotic species, i.e., *Nitrosopumilus maritimus* [41], *Cenarchaeum symbiosum* [42], and “*Ca*. Nitrososphaera gargensis” [29], which fix carbon in the hydroxypropionate–hydroxybutyrate (HP-HB) cycle [43, 44]. We found that *Ca*. C. subterraneum could not complete any of the carbon fixation modules and the MCR values were very low except for the reductive tricarboxylic acid (TCA) cycle (M00173) (Fig 4-1). Apparently, *Ca*. C. subterraneum had a high completion ratio of more than 90% for the reductive TCA cycle module, but the most important reaction step (steps 11_1 or 11_2 and 12) catalyzed by ATP-citrate lyase [45] or citryl-CoA synthetase and citryl-CoA lyase, which are the key enzymes in this cycle [46, 47], was not complete (Fig 4-2-A). The MCR of the reductive TCA cycle sometimes has a high value in other organisms because the enzymes used by this cycle are shared among many other pathway modules. For example, the enzymes used for steps 4 to 10 of the reductive TCA cycle (Fig 4-2-A) are shared in all steps of the TCA cycle, except for citrate synthase involved in step 1 (Fig 5-2-C). In fact, *Ca*. C. subterraneum possesses enzymes related to all reaction steps shared between both cycles. If *Ca*. C. subterraneum harbors different types of key enzymes for the reductive TCA cycle, it could perform carbon fixation, but it is parsimonious to infer that it has an extremely poor carbon fixation potential. The thaumarchaeotic species were also unable to complete any of the carbon fixation modules, but are known to possess the HP-HB cycle for carbon fixation (Fig 4-2B). The HP-HB cycle is not complete in thaumarchaeotic species because the genes responsible for 5 reaction steps (steps 2, 3, 6, 10, and 11), which are common to both crenarchaeotic and thaumarchaeotic HP-HB cycles, have not yet been identified in their genomes using bioinformatics methods [44]. The K numbers corresponding to the enzymes responsible for steps 10 (K18601: succinyl-CoA reductase) and 11 (K18602: succinate semialdehyde reductase) are assigned to the Nmar_1608 gene, and the paralogous Nmar_1110 and Nmar_161 genes, respectively, in the KEGG database; however, these K numbers were incorrectly assigned by KEGG. Recently, it has been experimentally confirmed that Nmar_1110 is malonic semialdehyde reductase, responsible for step 3 [48]. This misannotation by KEGG should be corrected immediately. The thaumarchaeotic HP-HB cycle, which is thought to have arisen independently in thaumarchaeal and crenarchaeal lineages by convergent evolution, is the most energy-efficient aerobic pathway for CO_2_ fixation, and the activities of all enzymes employed by the HP-HB cycle, which comprises 15 reaction steps, have been confirmed experimentally [44]. Thus, the remaining 4 unidentified genes that did not show significant homology to crenarchaeotic enzymes should be present in their genomes. Almost no enzymes for this cycle were identified bioinformatically in the *Ca*. C. subterraneum genome, but the overall MCR patterns for other carbon fixation modules were similar to those of thaumarchaeotic species (S1 Fig and S3 Table).

### MCR patterns involved in central metabolism and transporters

The carbon fixation potential of *Ca*. C. subterraneum was extremely poor despite previous suggestions that it has an autotrophic lifestyle [3, 13]. Thus, we analyzed the MCR patterns involved in central carbohydrate metabolism as well as the transporters related to sugar and amino acid transport, and compared the patterns with those of thaumarchaeotic species (S3 Fig and Fig 5-1). *Ca*. C. subterraneum did not complete the KEGG module for the Embden–Meyerhof (EM) pathway comprising 10 reaction steps because two enzymes responsible for step 3, ATP-dependent 6-phosphofructokinase (ATP-PFK) (K00850 or K16370) and ADP-dependent phosphofructokinase/glucokinase (K00918), were not identified in the genome (Fig 5-2-A1). However, another type of ATP-PFK that shows no sequence similarity to classical ATP-PFKs has been purified and characterized in the two crenarchaeotic species *Aeropyrum pernix* and *Desulfurococcus amylolyticus* [49–51]. Both enzymes are in the PFK-B family within the ribokinase superfamily based on sequence similarities. In the KEGG database, the orthologous group containing ATP-PFK from *A*. *pernix* (APE_0012) belonging to the PFK-B family is defined as ribokinase (EC: 2.7.1.15) and the different K number of K00852 is assigned to this orthologous group. At present, K00852 has not yet been assigned to the module for the EM pathway, but it should be assigned to reaction step 3 in this module together with currently assigned KOs. In the *Ca*. C. subterraneum genome, there are two K00852-assigned genes, CSUB_C0883 and CSUB_C1035, and APE_0012 and CSUB_C1035 share 74.4% similarity (ca. 30% identity). Thus, the gene product of CSUB_C1035 presumably acts as ATP-PFK, responsible for reaction step 3.

Similar to the enzymes involve in the reductive TCA cycle and TCA cycle, the enzymes used for 4 to 9 reaction steps in the EM pathway modules are shared with those involved in reaction steps 2 to 7 of the gluconeogenesis module (Fig 5-2-A2). Actually, *Ca*. C. subterraneum completed the gluconeogenesis module with a high MCR of 85.7%, although phosphoenolpyruvate carboxykinase, which is responsible for reaction step 1, was not identified. *Ca*. C. subterraneum possesses genes encoding malate dehydrogenase: oxaloacetate-decarboxylating (CSUB_C1205) and pyruvate orthophosphate dikinase (CSUB_C1231), which enable the conversion of malate to phosphoenolpyruvate via pyruvate. A series of reactions have been defined as the KEGG module for crassulacean acid metabolism (CAM)-light (M00169), comprising 2 reaction steps, and K00029 and K01006 were assigned to each step as shown in Fig 5E. The gene product of CSUB_C1205 shows ca. 85% similarity (56% identity) to the K00029-assigned gene (SSO_2869) from *Sulfolobus solfataricus* [52], but a different K number, K00027, is assigned to this gene in KEGG database. Accordingly *Ca*. C. subterraneum cannot complete the current M00169 module, to which K00027 is not assigned for unclear reasons. One of two specific enzymes in the module for gluconeogenesis, fructose 1,6-bisphosphate aldolase/phosphatase, was identified in both *Ca*. C. subterraneum and thaumarchaeotic species [53, 54]. *Ca*. C. subterraneum also completed the pyruvate oxidation module connected to the TCA cycle.

**Fig 6:**
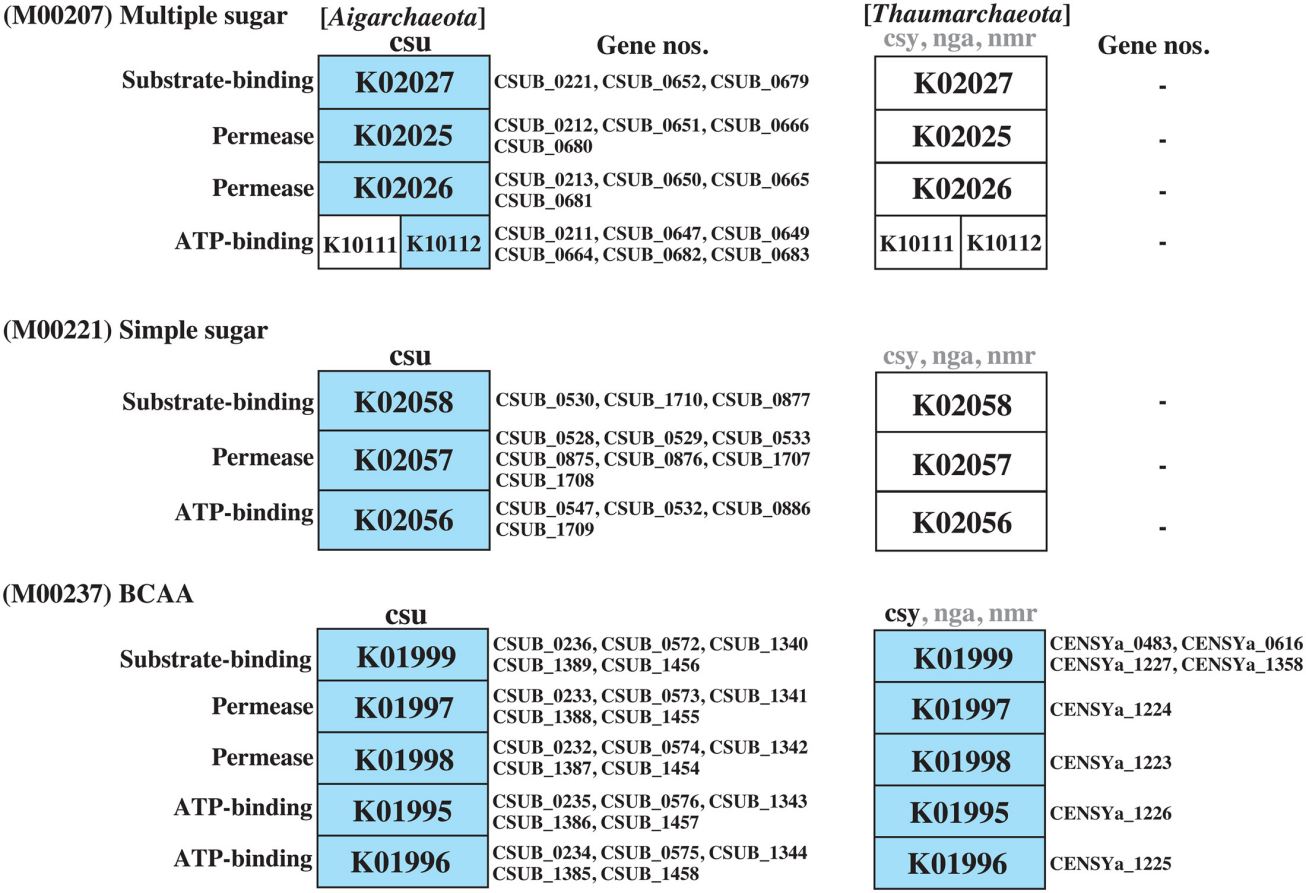
Sugar and blanched chain amino acid (BCAA) ABC transporters module abundance in *Ca*. C. subterraneum and thaumarchaeotic species. K numbers in blue boxes indicate the orthologous genes identified in each species. csu, *Ca*. Caldiarchaeum subterraneum; nmr, *Nitrosopumilus maritimus*; nga, *Ca*. Nitrososphaera gargensis; csy, *Cenarchaeum symbiosum*. Grayish characters indicate the species with no gene set in the module. M numbers indicate the module IDs defined by KEGG.

All of the thaumarchaeotic species showed a high MCR value for the EM pathway because they completed the gluconeogenesis modules. Two of three thaumarchaeotic species, *N*. *maritimus* and *C*. *symbiosum*, completed the EM pathway with an MCR of 70%, but lacked glucokinase (step 1) and pyruvate kinase (step 10) as well as the enzyme (6-phosphofructokinase or ADP-dependent phosphofructokinase/glucokinase) responsible for step 3 (S3 Fig). However, thermophilic “*Ca*. N. gargensis” possessed an ATP-PFK within the PFK-B family showing 75% similarity (ca. 32% identity) to that of *Ca*. C. subterraneum, unlike the two other thaumarchaeotic species. *Ca*. C. subterraneum possesses at least three sets of multiple sugar and simple sugar ABC transporters (M00207 and M00221), while the thaumarchaeotic species possess no sugar transporters as shown in Fig 6. Thus, this organism presumably has the ability to metabolize the sugars taken up by these transporters via the EM pathway.

*Ca*. C. subterraneum did not complete the current KEGG module for the TCA cycle, which comprises eight reaction steps; the genes encoding the γ (K00177) and δ (K00176) subunits of 2-oxoglutarate ferredoxin oxidoreductase (step 3) were not identified in the genome, but those for the α (K00174) and β (K00175) subunits were identified. Another enzyme type with a different subunit composition, such as an αβ (heterodimer), α_2_β_2_ (dimer of heterodimer), and α_2_ homodimer, has been identified [55–58]. However, these reaction components are not yet connected to step 4 in the current KEGG module; accordingly, this module should be improved to enable more precise bioinformatic characterization of archaeal metabolism. The gene products of CSUB_C1635 (α subunit) and CSUB_C1634 (β subunit) in the *Ca*. C. subterraneum genome show sequence similarity of more than 84% to each subunit of the αβ-type enzyme from *Sulfolobus tokodaii* strain 7 [55]. Thus, the αβ-type heterodimeric enzyme from *Ca*. C. subterraneum is thought to function in reaction step 4 of the TCA cycle module, similar to in *S*. *tokodaii*. In addition, *Ca*. C. subterraneum possesses the glyoxylate cycle to bypass the decarboxylation steps in the TCA cycle. As mentioned above, because this organism also possesses malate dehydrogenase (CSUB_C1205) and pyruvate orthophosphate dikinase (CSUB_C1231), which enable the conversion of malate to phosphoenolpyruvate, this cycle connected to the gluconeogenesis pathway is thought to be involved in the production of cell components. In contrast to *Ca*. C. subterraneum, thaumarchaeotic species possess almost none of the key enzymes responsible for the glyoxylate cycle (S3 Fig).

*Ca*. C. subterraneum possesses five sets of blanched chain amino acids (BCAA), such as leucine, isoleucine, and valine ABC transporters, whereas among the thaumarchaeotic species, *C*. *symbiosum* possesses only one set (Fig 6). Except for valine, BCAAs are converted to acetyl-CoA via the degradation pathways identified in many bacteria and eukaryotic species. The degradation pathways of leucine and isoleucine have not yet been identified in archaea, including *Ca*. C. subterraneum, although genes related to these pathways have been partially identified in their genomes. Thus, this organism may employ an unknown alternative pathway to convert these amino acids to acetyl-CoA. Moreover, a citrate transporter (CSUB_C0827), which is one of the metabolites in the TCA cycle that possibly stimulates growth, was identified in the *Ca*. C. subterraneum genome.

## Discussion

A primary objective of genomic analyses is to deduce the potential functionome of individual organisms. Since the genome of uncultured *Ferroplasma* type II [59] from a natural acidophilic biofilm has been nearly complete for more than a decade, uncultivated archaeal genomes, even within candidate phyla, have been recovered by metagenomics or a hybrid method of metagenomics and single-cell genomics from various environmental samples [9–11, 14, 60]. However, evaluating the potential functionome remains difficult because standard methodology has not yet been established to extract functional features such as those related to metabolism, energy generation, and transportation systems. Thus, we developed a new method to evaluate the potential functionome by calculating the MCRs for KEGG modules [19]. Recently, we launched the MAPLE system to automate a series of steps used in this method [20]. In the present study, we applied this new method to the functional classification of archaeal species and we determined the functional characteristics of the previously uncultured *Ca*. C. subterraneum in a comparative analysis with other archaea based on MCR patterns.

MCR is an easy-to-understand measure to evaluate functional potential. Generally, there is a correlation between the completeness of a KEGG module and the likelihood that an organism can perform the physiological function corresponding to the module. However, when KOs used for a module are shared with other independent modules, i.e., the reductive TCA cycle (Fig 4) and TCA cycle (Fig 5), the MCR does not necessarily reflect the working probability of each functional module. Thus, the relationship between the MCR of the targeted module and others to which the same KOs are assigned and the contribution of the specific KOs for each module to the MCR should be considered when a module is not complete. In this study, the MCR patterns of 273 modules, regardless of the relationships between modules, were subjected to a PCA to characterize the overall physiological features of archaea. Consequently, 79 archaea were classified into two groups (aerobes and anaerobes) according to PC1 and into 3 major taxonomic groups, *Euryarchaeota*, *Crenarchaeota*, and *Thaumarchaeota*, according to PC2. These interpretations according to the loadings in PC1 and PC2 are biologically plausible (see Results). However, some exceptions were observed. For example, some euryarchaeal species belonged to the *Crenarchaeota* group. These outliers can be explained by the low percentages of variance along PC1 and PC2 (Fig 3). In general, a PCA provides understandable explanation by reducing high-dimensional features (i.e., the series of reaction modules, in this study) and by avoiding problems associated with multicollinearity, i.e., overlap between modules; however, such a reduction in dimensionality was not effective in this study owing to the data complexity. Thus, we are unable to provide a deeper discussion. The difficulty in interpreting the results is a known limitation of the PCA [61]. Accordingly, higher-level statistical analyses are necessary, such as the sparse PCA [61]. However, we did not use the sparse PCA owing to the higher computational cost. For a more detailed analysis, additional physiological properties (i.e., growth temperature and nutrient requirements) of archaea are also necessary for biologically meaningful interpretations of the principle components. In this study, the relationship between physiological/taxonomic properties and PCs was not comprehensively evaluated owing to the lack of physiological data for each species, excluding oxygen requirement. Thus, it is possible that oxygen requirement and taxonomic groups are not optimal descriptors of PC1 and PC2, respectively. Some data on physiological properties is available [62]; however, the collection of more physiological properties of archaeal species is important for future studies.

The genome of *Ca*. C. subterraneum was reconstructed from a metagenomic library obtained from a subsurface thermophilic microbial mat community in a 70°C hot water stream in a Japanese epithermal mine [14]. Because this hot water stream contains low levels of organic matter, the microbial mat community at the oxic–anoxic interface is considered to be supported by geological energy and carbon sources such as CO_2_ and CH_4_, which are supplied by the geological aquifer [63–65]. The microbial mat comprised at least 16 prokaryotic phylotypes, including phyla such as *Chloroflexi*, *Proteobacteria*, *Deinococcus-Thermus*, and *Thaumarchaeota*. However, *Ca*. C. subterraneum was dominant together with “*Candidatus* Acetothermus autotrophicum” (*Ca*. A. autotrophicum), the genomic sequence of which has almost been completed [15]. The genomic analysis of *Ca*. A. autotrophicum demonstrated its chemolithoautotrophic potential based on homo-acetogenesis via H_2_ and CO_2_, although the detailed mechanisms for energy generation still remain unclear. In contrast to *Ca*. A. autotrophicum, *Ca*. C. subterraneum has an extremely poor carbon fixation potential, but completed the modules responsible for major central carbohydrate metabolism. *Ca*. C. subterraneum also possesses all enzymes responsible for the glyoxylate cycle, which has been well studied in *Escherichia coli* [66]. *E*. *coli* can grow using the glyoxylate cycle, which is an anaplerotic pathway of the TCA cycle when acetate serves as the sole carbon source for growth. Therefore, *Ca*. C. subterraneum also may use acetate produced by acetogenic *Ca*. A. autotrophicum as a carbon source for growth in the microbial mat community. Moreover, *Ca*. C. subterraneum possesses multiple sets of ABC transporters required for the uptake of sugars and BCAAs, thereby supporting its heterotrophic lifestyle in the microbial mat community. In addition to *Ca*. A. autotrophicum, another potentially chemolithoautotrophic phylotype that is closely related to “*Ca*. Nitrosocaldus yellowstonii” (*Thaumarchaeota*), *Hydrogenobacter thermophilus* (*Aquificae*), *Hydrogenophilus thermoluteolus* (*Betaproteobacteria*), and a methanotrophic phylotype similar to *Methylohalobius crimensis* (*Gammaproteobacteria*), which probably use CO_2_ and CH_4_ supplied by the geothermal aquifer, coexist with *Ca*. C. subterraneum. However, the abundance of these phylotypes was low in the microbial mat community [15]. Given the abundance and diversity of these chemolithoautotrophic and methanotrophic phylotypes, they may serve as the primary energy and carbon source suppliers in the microbial mat community, and contribute to the primary production of the heterotrophic phylotypes, including *Ca*. C. subterraneum in the anaerobic organic-depleted state.

In conclusion, using the MAPLE system, the archaeal species registered in the KEGG GENOME database were classified into various groups that possess similar phenotypic properties, such as halophiles, methanogens, ammonia oxidizers, heterotrophic (hyper)-thermophilic acidophiles or neutrophiles, and hyperthermophilic facultative autotrophic sulfur reducers. Moreover, methanogens were classified into two major groups with different ecological niches, the human gut and other earth environments. The functional classification of previously uncultivated *Ca*. C. subterraneum using the MAPLE system and subsequent metabolic analyses based on the MCR patterns of individual KEGG modules, supported the predominantly aerobic nature of *Ca*. C. subterraneum, which lives in a subsurface thermophilic microbial mat community with a heterotrophic lifestyle. However, it is still challenging for the archaeal science community to elucidate metabolic features using bioinformatics methods because many of the unusual features of archaeal metabolism revealed to date are not reflected in the KEGG modules. Thus, the MAPLE system will be more helpful for characterizing archaeal metabolism, even in uncultured archaea, when the KEGG modules incorporate more archaeal metabolic features. Also, manual data curation and literature mining are still important strategies when implement MAPLE.

## Supporting Information

S1 Fig Comparison of the module completion ratio (MCR) patterns of uncultured *“Ca*. C. Subterraneum” and those of thaumarchaeotic species. (PDF)

S2 Fig Major central carbohydrate metabolism in thaumarchaeotic species. (PDF)

S3 Fig Functional modules that contributed to the variance in the first and second principal axes in the principle components analysis (PCA) of MCR patterns of 79 archaeal species. (PDF)

S1 Table List of organisms used to produce the phylograms in Fig 1. (PDF)

S2 Table List of archaeal species used to evaluate the physiological and metabolic potential with the MAPLE system. (PDF)

S3 Table Module completion patterns for the 79 archaeal species listed in S2 Table. (PDF)
