# Supplementary material for: Functional Classification of Uncultured “Candidatus Caldiarchaeum subterraneum” Using the Maple System
Source: PLoS One. 2015 Jul 21;10(7):e0132994. doi: 10.1371/journal.pone.0132994 (PMC4510362; doi:10.1371/journal.pone.0132994)
Supplement: S1 Fig — (PDF) [file pone.0132994.s001.pdf]

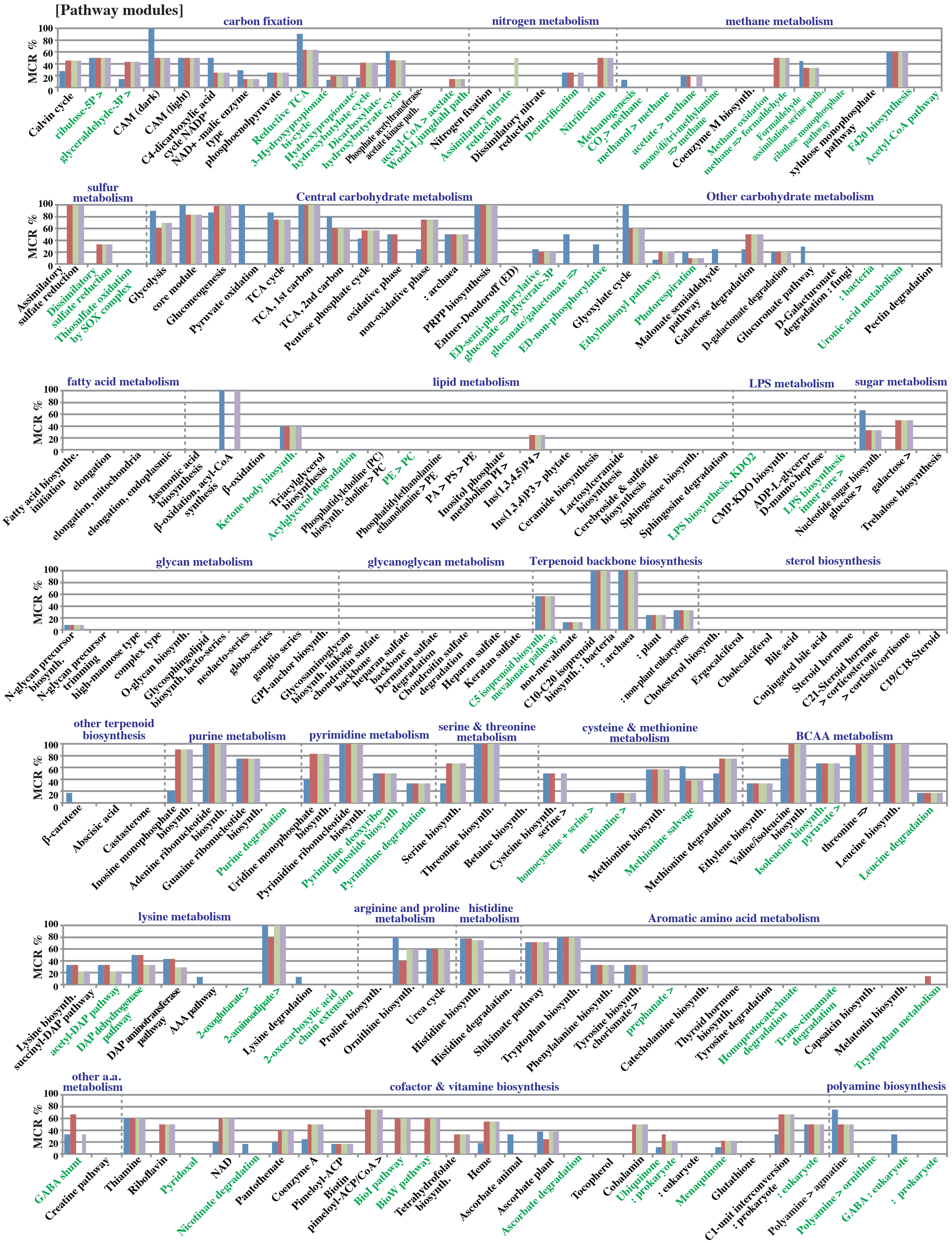

Figure S1 Comparison of the MCR pattern of uncultured *Ca* . 'Caldiarchaeum subterraneum' with those of representative species within *Thaumarchaeota*. MCR: module completion ratio, Green shows restricted modules [19], Complete name of each module is described in Table S3. ■, *Ca*. 'Caldiarchaeum subterraneum' ; ■, *Ca*. 'Nitrososphaera gargensis' ; ■, *Cenarchaeum symbiosum*; ■, *Nitrosopumilus maritimus*

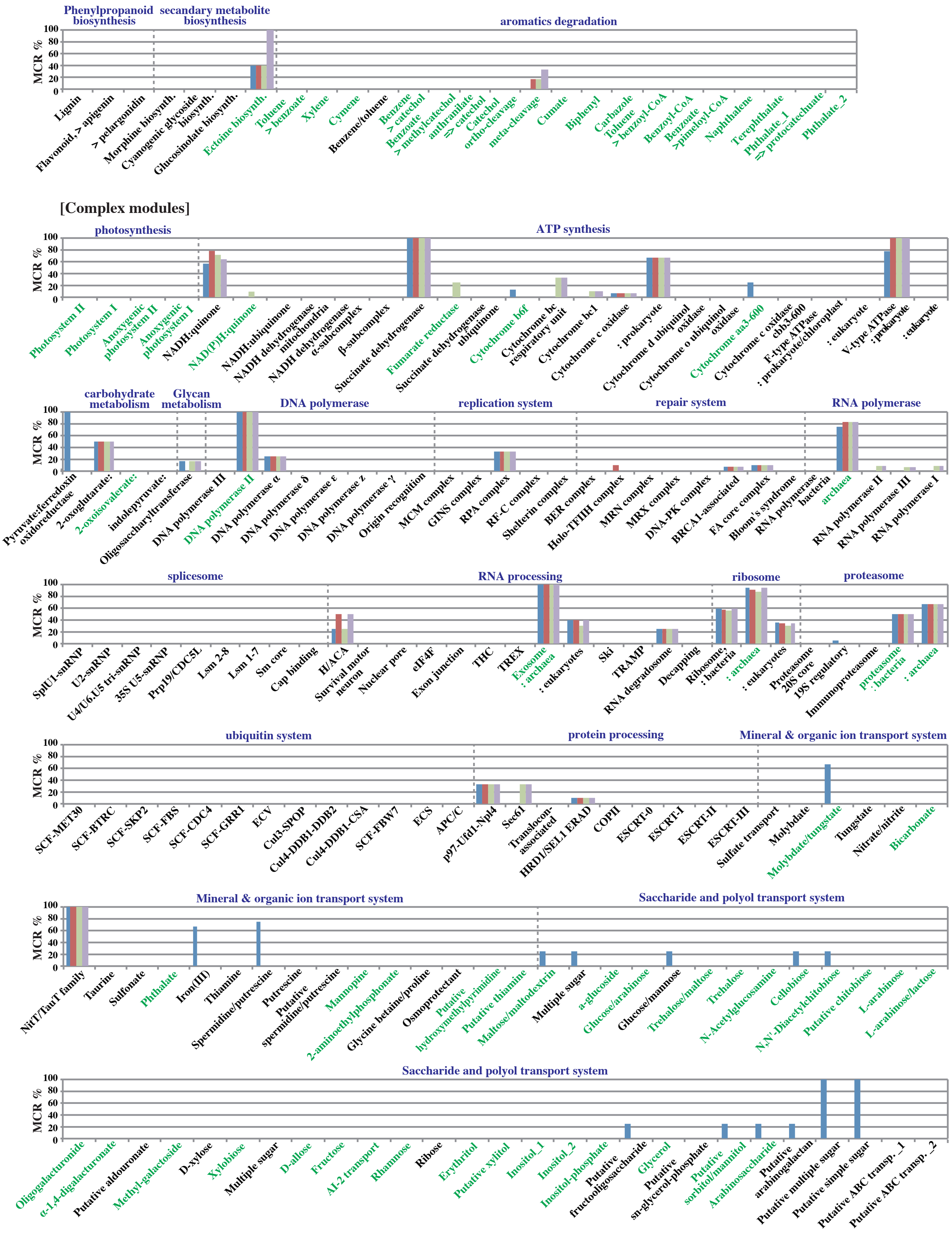

Figure S1 Comparison of module completion pattern of uncultured Ca. "Caidiarchaeum" with those of representative species within *Thaumarchaeota*. continued

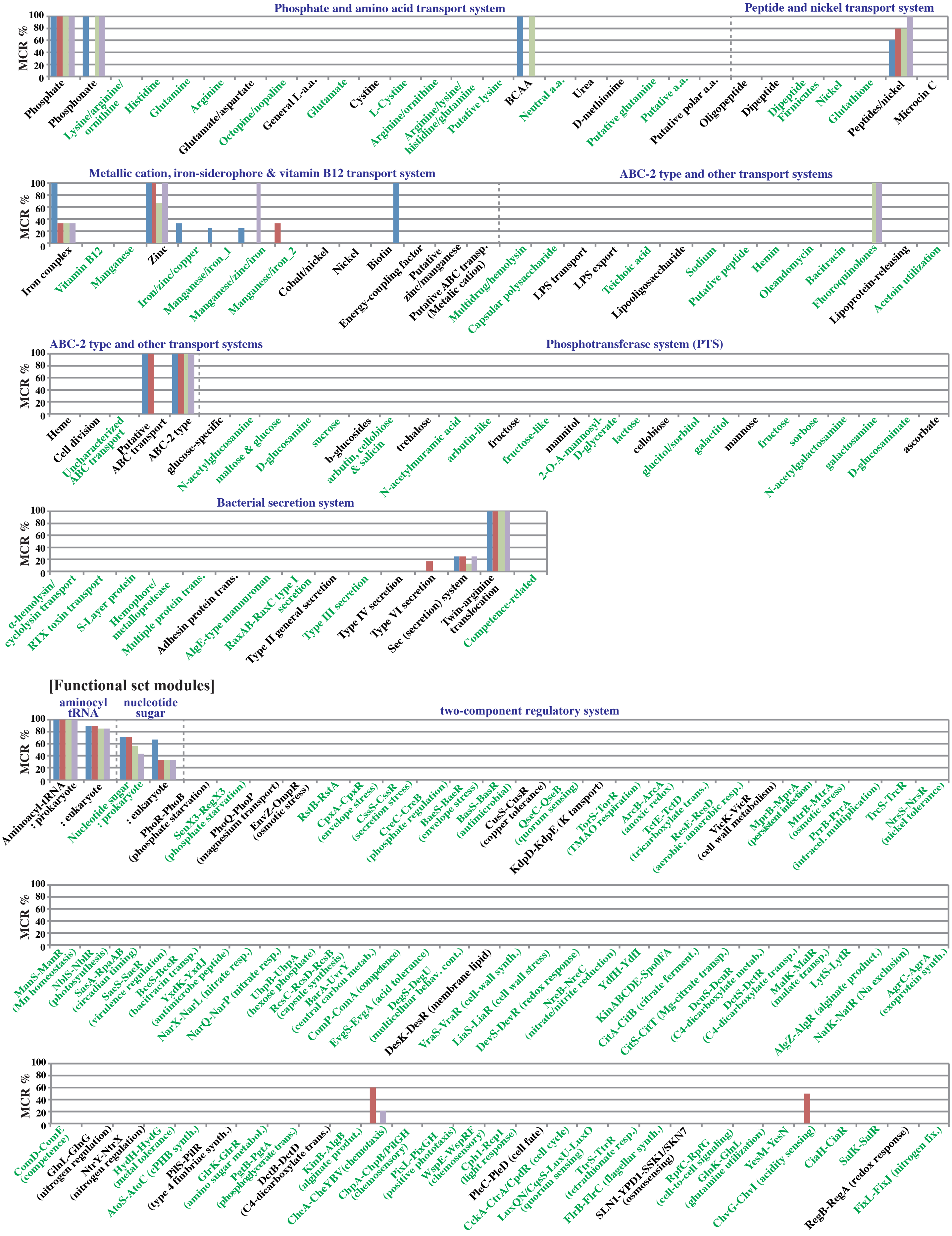

Figure S1 Comparison of module completion pattern of uncultured Ca. "Caidiarchaeum" with those of representative species within *Thaumarchaeota*. continued
