# Supplementary material for: Functional Classification of Uncultured “Candidatus Caldiarchaeum subterraneum” Using the Maple System
Source: PLoS One. 2015 Jul 21;10(7):e0132994. doi: 10.1371/journal.pone.0132994 (PMC4510362; doi:10.1371/journal.pone.0132994)
Supplement: S2 Fig — (PDF) [file pone.0132994.s002.pdf]

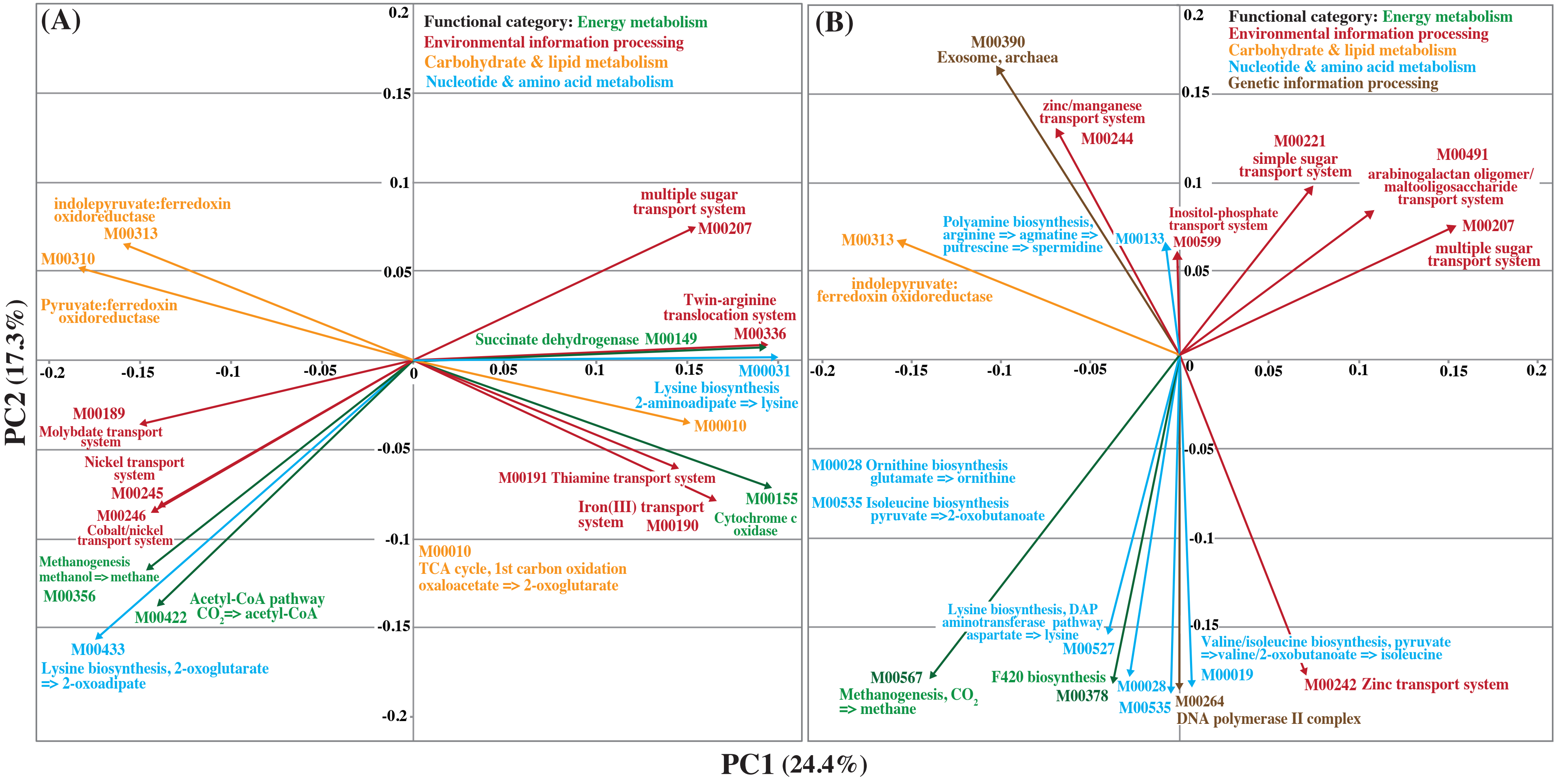

Figure S2 Functional modules contributing the variance at the first and second principal axes in PCA of the MCR patterns of 79 archaeal species. (A) Modules with high factor loding on the PC1. (B) Modules with high factor loading on the PC2. Each color shows large functional categories defined by KEGG (http://www.genome.jp/kegg-bin/get_htext?ko00002.keg).
