# Supplementary material for: Functional Classification of Uncultured “Candidatus Caldiarchaeum subterraneum” Using the Maple System
Source: PLoS One. 2015 Jul 21;10(7):e0132994. doi: 10.1371/journal.pone.0132994 (PMC4510362; doi:10.1371/journal.pone.0132994)
Supplement: S3 Fig — (PDF) [file pone.0132994.s003.pdf]

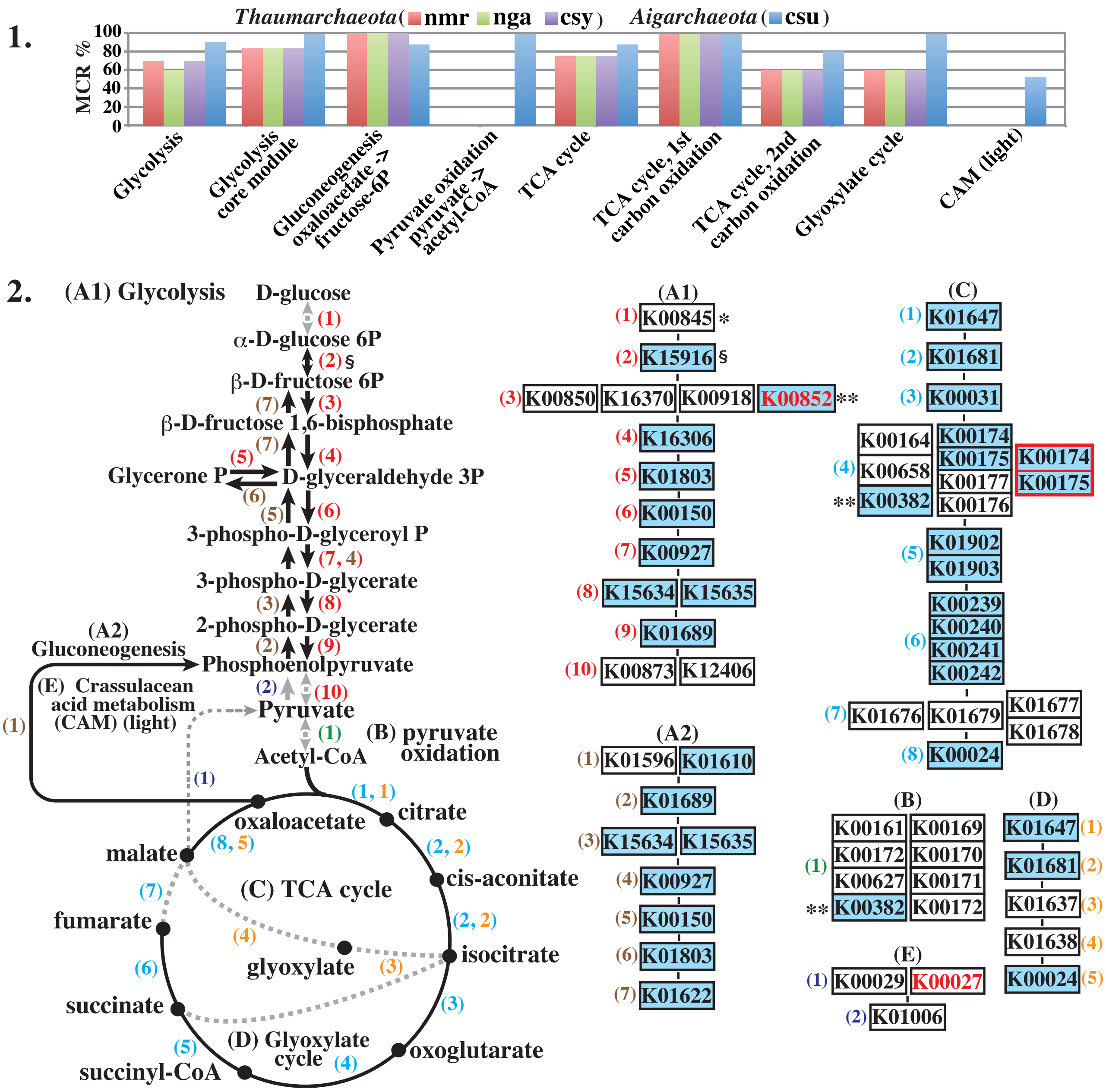

Figure S3 Major central carbohydrate metabolism in the representative species within *Thaumarchaeota* **1**. Comparision of the module completion patterns of thaumarchaeotic and aigarchaeotic species. nmr, *Nitrosopumilus maritimus* ; nga, *Ca*. 'Nitrososphaera gargensis' ; csy, *Cenarchaeum symbiosum*; csu; *Ca*. 'Caldiarchaum subterraneum' . **2**. Pathway map for central carbohydrate metabolism (left side) and mapping pattern of the modules corresponding to the pathway map in three thaumarchaotic species (right side). In each K number set comprising components of the module, vertically connected and horizontally located K numbers indicate complex and alternatives, respectively [19]. K numbers in blue boxes indicate the orthologous genes identified in the thaumarchaeotic species. *, Other 5 orthologous genes (K00844, K12407, K00886, K08074, and K00918) are omitted in this figure due to drawing space. §, Missing only in nga. **, Missing in nir and csy. Grayish dashed lines and arrows shows missing reaction steps in the pathway. Numbers in parentheses on each module component correspond to those on the pathway map. K numbers shown in red have not been assigned to the KEGG module although their similar enzymatic reactions to the K numbers assigned to the module have been confirmed. The reaction component shown by red frame in the TCA cycle shows αβ-heterodimeric 2-oxoacid: ferredoxin oxidereductase [55, 56], which has not been reflected to the KEGG module.
