# Supplementary material for: Functional Classification of Uncultured “Candidatus Caldiarchaeum subterraneum” Using the Maple System
Source: PLoS One. 2015 Jul 21;10(7):e0132994. doi: 10.1371/journal.pone.0132994 (PMC4510362; doi:10.1371/journal.pone.0132994)
Supplement: S1 Table — (PDF) [file pone.0132994.s004.pdf]

Table S1 List of organisms used for phylograms in Figure 1

Superkingdom	Phylum	Species	Species ID	family	Figure
Archaea	*Aigarchaeota*	*Caldiarchaeum subterranum*	csu	Unclassified	A, B
	Crenarchaeota	*Aeropyrum camini*	acj	*Desulfurococcaceae*	A, B
		Acidianus hospitalis	aho	*Sulfolobaceae*	A, B
		Aeropyrum pernix	ape	*Desulfurococcaceae*	A, B
		Acidilobus saccharovorans	asc	*Acidilobaceae*	A, B
		Caldisphaera lagunensis	clg	*Caldisphaeraceae*	A, B
		Caldivirga maquilingensis	cma	*Thermoproteaceae*	A, B
		Desulfurococcus fermentans	dfd	*Desulfurococcaceae*	A, B
		Desulfurococcus kamchatkensis	dka	*Desulfurococcaceae*	A
		Desulfurococcus mucosus	dmu	*Desulfurococcaceae*	A, B
		Fervidicoccus fontis	ffo	*Fervidicoccaceae*	A, B
		Hyperthermus butylicus	hbu	*Pyrodictiaceae*	A, B
		Ignisphaera aggregans	iag	*Desulfurococcaceae*	A, B
		Ignicoccus hospitalis	iho	*Desulfurococcaceae*	A, B
		Metallosphaera cuprina	mcn	*Sulfolobaceae*	A, B
		Metallosphaera sedula	mse	*Sulfolobaceae*	A, B
		Pyrobaculum aerophilum	pai	*Thermoproteaceae*	A, B
		Pyrobaculum arsenaticum	pas	*Thermoproteaceae*	A, B
		Pyrobaculum calidifontis	pcl	*Thermoproteaceae*	A, B
		Pyrolobus fumarii	pfm	*Pyrodictiaceae*	A, B
		Pyrobaculum islandicum	pis	*Thermoproteaceae*	A, B
		Pyrobaculum oguniense	pog	*Thermoproteaceae*	A, B
		Pyrobaculum sp.	pyr	*Thermoproteaceae*	A
		Sulfolobus acidocaldarius	sai	*Sulfolobaceae*	A, B
		Staphylothermus hellenicus	shc	*Desulfurococcaceae*	A, B
		Sulfolobus islandicus	sih	*Sulfolobaceae*	A, B
		Staphylothermus marinus	smr	*Desulfurococcaceae*	A, B
		Sulfolobus solfataricus	sol	*Sulfolobaceae*	A, B
		Sulfolobus tokodaii	sto	*Sulfolobaceae*	A, B
		Thermosphaera aggregans	tag	*Desulfurococcaceae*	A, B
		Thermofilum sp.	thb	*Thermofilaceae*	A
		Thermogladius cellulolyticus	thg	*Desulfurococcaceae*	A, B
		Pyrobaculum neutrophilum	tne	*Thermoproteaceae*	A, B
		Thermofilum pendens	tpe	*Thermofilaceae*	A, B
		Thermoproteus tenax	ttn	*Thermoproteaceae*	A, B
		Thermoproteus uzoniensis	tuz	*Thermoproteaceae*	A, B
		Vulcanisaeta distributa	vdi	*Thermoproteaceae*	A, B
		Vulcanisaeta moutnovskia	vmo	*Thermoproteaceae*	A, B
	Euryarchaeota	*Aciduliprofundum boonei*	abi	Unclassified	A, B
		Aciduliprofundum sp.	acf	Unclassified	A, B
		Archaeoglobus fulgidus	afu	*Archaeoglobaceae*	A, B
		Archaeoglobus profundus	apo	*Archaeoglobaceae*	A, B
		Archaeoglobus sulfaticallidus	ast	*Archaeoglobaceae*	A
		Archaeoglobus veneficus	ave	*Archaeoglobaceae*	A, B
		Ferroplasma acidarmanus	fac	*Ferroplasmaceae*	A, B
		Ferroglobus placidus	fpl	*Archaeoglobaceae*	A, B

Halobacterium salinarum	hal	*Halobacteriaceae*	A, B
Halogeometricum borinquense	hbo	*Halobacteriaceae*	A, B
Haloarcula hispanica	hhi	*Halobacteriaceae*	A, B
Halalkalicoccus jeotgali	hje	*Halobacteriaceae*	A, B
Halorubrum lacusprofundi	hla	*Halobacteriaceae*	A, B
Haloarcula marismortui	hma	*Halobacteriaceae*	A, B
Haloferax mediterranei	hme	*Halobacteriaceae*	A, B
Halomicrobium mukohataei	hmu	*Halobacteriaceae*	A, B
Halovivax ruber	hru	*Halobacteriaceae*	A, B
Halobacterium salinarum	hsl	*Halobacteriaceae*	A, B
Halorhabdus tiamatea	hti	*Halobacteriaceae*	A, B
Haloterrigena turkmenica	htu	*Halobacteriaceae*	A, B
Halorhabdus utahensis	hut	*Halobacteriaceae*	A, B
Haloferax volcanii	hvo	*Halobacteriaceae*	A, B
Haloquadratum walsbyi	hwc	*Halobacteriaceae*	A, B
Halopiger xanaduensis	hxa	*Halobacteriaceae*	A, B
Methanosarcina acetivorans	mac	*Methanosarcinaceae*	A, B
Methanococcus aeolicus	mae	*Methanococcaceae*	A, B
Methanomethylophilus alvus	max	Unclassified	A, B
Methanosarcina barkeri	mba	*Methanosarcinaceae*	A, B
Methanoculleus bourgensis	mbg	*Methanomicrobiaceae*	A, B
Methanoregula boonei	mbn	*Methanoregulaceae*	A, B
Methanococcoides burtonii	mbu	*Methanosarcinaceae*	A, B
Methanosaeta concilii	mcj	*Methanosaetaceae*	A, B
Methanobrevibacter sp.	meb	*Methanobacteriaceae*	A, B
Methanobacterium sp.	mel	*Methanobacteriaceae*	A, B
Methanoculleus marisnigri	mem	*Methanomicrobiaceae*	A, B
Methanomassiliicoccus intestinalis	mer	Unclassified	A, B
Methanohalobium evestigatum	mev	*Methanosarcinaceae*	A, B
Methanobacterium sp.	mew	*Methanobacteriaceae*	A, B
Methanocella conradii	mez	*Methanocellaceae*	A, B
Methanocaldococcus fervens	mfe	*Methanocaldococcaceae*	A, B
Methanoregula formicica	mfo	*Methanoregulaceae*	A, B
Methanocaldococcus sp.	mfs	*Methanocaldococcaceae*	A, B
Methanothermus fervidus	mfv	*Methanothermaceae*	A, B
Methanosaeta harundinacea	mhi	*Methanosaetaceae*	A, B
Methanospirillum hungatei	mhu	*Methanospirillaceae*	A, B
Methanomethylovorans hollandica	mhz	*Methanosarcinaceae*	A, B
Methanocaldococcus infernus	mif	*Methanocaldococcaceae*	A, B
Methanotorris igneus	mig	*Methanocaldococcaceae*	A, B
Methanocaldococcus jannaschii	mja	*Methanocaldococcaceae*	A, B
Methanopyrus kandleri	mka	*Methanopyraceae*	A, B
Methanocorpusculum labreanum	mla	*Methanocorpusculaceae*	A, B
Methanosarcina mazei	mma	*Methanosarcinaceae*	A, B
Methanothermobacter marburgensis	mmg	*Methanobacteriaceae*	A, B
Methanohalophilus mahii	mmh	*Methanosarcinaceae*	A, B
Methanococcus maripaludis	mmq	*Methanococcaceae*	A, B
Methanococcus maripaludis	mmz	*Methanococcaceae*	A
Methanothermococcus okinawensis	mok	*Methanococcaceae*	A, B

		Methanocella paludicola	mpd	*Methanocellaceae*	A, B
		Methanoplanus petrolearius	mpi	*Methanomicrobiaceae*	A, B
		Methanosphaerula palustris	mpl	*Methanoregulaceae*	A, B
		Methanolobus psychrophilus	mpy	*Methanosarcinaceae*	A, B
		Methanobrevibacter ruminantium	mru	*Methanobacteriaceae*	A, B
		Methanobrevibacter smithii	msi	*Methanobacteriaceae*	A, B
		Methanosphaera stadtmanae	mst	*Methanobacteriaceae*	A, B
		Methanothermobacter thermautotrophicus	mth	*Methanobacteriaceae*	A, B
		Methanosaeta thermophila	mtp	*Methanosaetaceae*	A, B
		Methanococcus vannielii	mvn	*Methanococcaceae*	A, B
		Methanococcus voltae	mvo	*Methanococcaceae*	A, B
		Methanocaldococcus vulcanius	mvu	*Methanocaldococcaceae*	A, B
		Methanosalsum zhilinae	mzh	*Methanosarcinaceae*	A, B
		Natrinema sp.	nat	*Halobacteriaceae*	A, B
		Natronobacterium gregoryi	nge	*Halobacteriaceae*	A, B
		Natrialba magadii	nmg	*Halobacteriaceae*	A, B
		Natronomonas moolapensis	nmo	*Halobacteriaceae*	A, B
		Natronococcus occultus	nou	*Halobacteriaceae*	A, B
		Natrinema pellirubrum	npe	*Halobacteriaceae*	A, B
		Natronomonas pharaonis	nph	*Halobacteriaceae*	A, B
		Pyrococcus abyssi	pab	*Thermococcaceae*	A, B
		Pyrococcus furiosus	pfi	*Thermococcaceae*	A, B
		Pyrococcus horikoshii	pho	*Thermococcaceae*	A, B
		Picrophilus torridus	pto	*Picrophilaceae*	A, B
		Pyrococcus yayanosii	pya	*Thermococcaceae*	A, B
		Pyrococcus sp.	pyn	*Thermococcaceae*	A, B
		Pyrococcus sp.	pys	*Thermococcaceae*	A
		Methanocella arvoryzae	rci	*Methanocellaceae*	A, B
		Salinarchaeum sp.	sali	*Halobacteriaceae*	A, B
		Thermoplasma acidophilum	tac	*Thermoplasmataceae*	A, B
		Thermoplasmatales archaeon	tar	Unclassified	A, B
		Thermococcus barophilus	tba	*Thermococcaceae*	A, B
		Thermococcus gammatolerans	tga	*Thermococcaceae*	A, B
		Thermococcus sp.	the	*Thermococcaceae*	A, B
		Thermococcus kodakarensis	tko	*Thermococcaceae*	A, B
		Thermococcus litoralis	tlt	*Thermococcaceae*	A, B
		Thermococcus onnurineus	ton	*Thermococcaceae*	A, B
		Thermococcus sibiricus	tsi	*Thermococcaceae*	A, B
		Thermoplasma volcanium	tvo	*Thermoplasmataceae*	A, B
	Korarchaeota	*Korarchaeum cryptofilum*	kcr	Unclassified	A, B
	Nanoarchaeota	*Nanoarchaeum equitans*	neq	Unclassified	A, B
	Thaumarchaeota	*Cenarchaeum symbiosum*	csy	*Cenarchaeaceae*	A, B
		Nitrososphaera gargensis	nga	Nitrososphaeraceae	A, B
		Nitrosopumilus sp.	nir	Nitrosopumilaceae	A, B
		Nitrosoarchaeum koreensis	nkr	Nitrosopumilaceae	A, B
		Nitrosopumilus maritimus	nmr	Nitrosopumilaceae	A, B
	Unclassified	*halophilic archaeon*	hah	Unclassified	A, B
Bacteria	*Firmicutes*	*Bacillus subtilis*	bsu	*Bacillaceae*	A
	γ-proteobacteria	*Escherichia coli*	eco	*Enterobacteriaceae*	A

Eukaryote	*Ascomycetes*	*Aspergillus fumigatus*	afm	*Aspergillaceae*	B
		Fusarium graminearum	fgr	*Nectriaceae*	B
		Thielavia terrestris	ttt	*Chaetomiaceae*	B
		Neurospora crassa	ncr	*Sordariaceae*	B
		Debaryomyces hansenii	dha	*Debaryomycetaceae*	B
		Yarrowia lipolytica	yli	*Dipodascaceae*	B
		Eremothecium cymbalariae	erc	*Saccharomycetaceae*	B
		Kazachstania africana	kaf	*Saccharomycetaceae*	B
		Pichia pastoris	ppa	*Phaffomycetaceae*	B
		Naumovozyma castellii	ncs	*Saccharomycetaceae*	B
		Saccharomyces cerevisiae	sce	*Saccharomycetaceae*	B
	Basidiomycetes	*Cryptococcus neoformans* JEC21	cne	*Tremellaceae*	B
	Microsporidians	*Encephalitozoon cuniculi*	ecu	*Unikaryonidae*	B
