# Supplementary material for: Functional Classification of Uncultured “Candidatus Caldiarchaeum subterraneum” Using the Maple System
Source: PLoS One. 2015 Jul 21;10(7):e0132994. doi: 10.1371/journal.pone.0132994 (PMC4510362; doi:10.1371/journal.pone.0132994)
Supplement: S2 Table — (PDF) [file pone.0132994.s005.pdf]

Table S2 List of the archaeal species used for evaluation of physiological and metabolic potential by MAPLE system

Species	Species ID	Class	Order
[***Euryarchaeota***: 53 species]			
Methanocaldococcus jannaschii	mja	*Methanococci*	*Methanococcales*
Methanotorris igneus	mig	*Methanococci*	*Methanococcales*
Methanococcus vannielii	mvn	*Methanococci*	*Methanococcales*
Methanothermococcus okinawensis	mok	*Methanococci*	*Methanococcales*
Methanosarcina barkeri	mba	*Methanomicrobia*	*Methanosarcinales*
Methanococcoides burtonii	mbu	*Methanomicrobia*	*Methanosarcinales*
Methanohalophilus mahii	mmh	*Methanomicrobia*	*Methanosarcinales*
Methanohalobium evestigatum	mev	*Methanomicrobia*	*Methanosarcinales*
Methanosalsum zhilinae	mzh	*Methanomicrobia*	*Methanosarcinales*
Methanolobus psychrophilus	mpy	*Methanomicrobia*	*Methanosarcinales*
Methanomethylovorans hollandica	mhz	*Methanomicrobia*	*Methanosarcinales*
Methanosaeta concilii	mcj	*Methanomicrobia*	*Methanosarcinales*
Methanospirillum hungatei	mhu	*Methanomicrobia*	*Methanomicrobiales*
Methanocorpusculum labreanum	mla	*Methanomicrobia*	*Methanomicrobiales*
Methanoculleus bourgensis	mbg	*Methanomicrobia*	*Methanomicrobiales*
Methanoplanus petrolearius	mpi	*Methanomicrobia*	*Methanomicrobiales*
Methanoregula boonei	mbn	*Methanomicrobia*	*Methanomicrobiales*
Candidatus Methanosphaerula palustris	mpl	*Methanomicrobia*	*Methanomicrobiales*
Methanocella paludicola	mpd	*Methanomicrobia*	*Methanocellales*
Methanomassiliicoccus sp. Mx1-Issoire	mer	*Methanomicrobia*	Unclassified
Methanothermobacter thermautotrophicus	mth	*Methanobacteria*	*Methanobacteriales*
Methanosphaera stadtmanae	mst	*Methanobacteria*	*Methanobacteriales*
Methanobrevibacter ruminantium	mru	*Methanobacteria*	*Methanobacteriales*
Methanobacterium lacus	mel	*Methanobacteria*	*Methanobacteriales*
Methanothermus fervidus	mfv	*Methanobacteria*	*Methanobacteriales*
Methanopyrus kandleri	mka	*Methanopyri*	*Methanopyrales*
Candidatus Methanomethylophilus alvus	max	Unclassified	Unclassified
Archaeoglobus fulgidus DSM 4304	afu	*Archaeoglobi*	*Archaeoglobales*
Ferroglobus placidus	fpl	*Archaeoglobi*	*Archaeoglobales*
Halobacterium salinarum	hsl	*Halobacteria*	*Halobacteriales*
Haloarcula marismortui	hma	*Halobacteria*	*Halobacteriales*
Haloquadratum walsbyi DSM 16790	hwa	*Halobacteria*	*Halobacteriales*
Natronomonas pharaonis	nph	*Halobacteria*	*Halobacteriales*
Halorubrum lacusprofundi	hla	*Halobacteria*	*Halobacteriales*
Halorhabdus utahensis	hut	*Halobacteria*	*Halobacteriales*
Halomicrobium mukohataei	hmu	*Halobacteria*	*Halobacteriales*
Haloterrigena turkmenica	htu	*Halobacteria*	*Halobacteriales*
Natrialba magadii	nmg	*Halobacteria*	*Halobacteriales*
Haloferax volcanii	hvo	*Halobacteria*	*Halobacteriales*
Halalkalicoccus jeotgali	hje	*Halobacteria*	*Halobacteriales*

Halogeometricum borinquense	hbo	*Halobacteria*	*Halobacteriales*
Halopiger xanaduensis	hxa	*Halobacteria*	*Halobacteriales*
Natrinema pellirubrum	npe	*Halobacteria*	*Halobacteriales*
Natronobacterium gregoryi	nge	*Halobacteria*	*Halobacteriales*
Halovivax ruber	hru	*Halobacteria*	*Halobacteriales*
Natronococcus occultus	nou	*Halobacteria*	*Halobacteriales*
Salinarchaeum sp. Harcht-Bsk1	sali	*Halobacteria*	*Halobacteriales*
Thermoplasma acidophilum	tac	*Thermoplasmata*	*Thermoplasmatales*
Picrophilus torridus	pto	*Thermoplasmata*	*Thermoplasmatales*
Ferroplasma acidarmanus	fac	*Thermoplasmata*	*Thermoplasmatales*
Pyrococcus furiosus DSM 3638	pfu	*Thermococci*	*Thermococcales*
Thermococcus kodakarensis	tko	*Thermococci*	*Thermococcales*
Aciduliprofundum boonei	abi	Unclassified	Unclassified
[***Crenarchaeota***: 20 species]			
Aeropyrum pernix	ape	*Thermoprotei*	*Desulfurococcales*
Staphylothermus marinus	smr	*Thermoprotei*	*Desulfurococcales*
Ignicoccus hospitalis	iho	*Thermoprotei*	*Desulfurococcales*
Desulfurococcus mucosus	dmu	*Thermoprotei*	*Desulfurococcales*
Thermosphaera aggregans	tag	*Thermoprotei*	*Desulfurococcales*
Ignisphaera aggregans	iag	*Thermoprotei*	*Desulfurococcales*
Thermogladius cellulolyticus	thg	*Thermoprotei*	*Desulfurococcales*
Hyperthermus butylicus	hbu	*Thermoprotei*	*Desulfurococcales*
Pyrolobus fumarii	pfm	*Thermoprotei*	*Desulfurococcales*
Sulfolobus acidocaldarius DSM 639	sai	*Thermoprotei*	*Sulfolobales*
Metallosphaera sedula	mse	*Thermoprotei*	*Sulfolobales*
Acidianus hospitalis	aho	*Thermoprotei*	*Sulfolobales*
Pyrobaculum islandicum	pis	*Thermoprotei*	*Thermoproteales*
Caldivirga maquilingensis	cma	*Thermoprotei*	*Thermoproteales*
Thermoproteus tenax	ttn	*Thermoprotei*	*Thermoproteales*
Vulcanisaeta distributa	vdi	*Thermoprotei*	*Thermoproteales*
Thermofilum pendens	tpe	*Thermoprotei*	*Thermoproteales*
Acidilobus saccharovorans	asc	*Thermoprotei*	*Acidilobales*
Caldisphaera lagunensis	clg	*Thermoprotei*	*Acidilobales*
Fervidicoccus fontis	ffo	*Thermoprotei*	*Fervidicoccales*
[***Thaumarchaeota***: 3 species]			
Nitrosopumilus maritimus	nmr	Unclassified	*Nitrosopumilales*
Cenarchaeum symbiosum	csy	Unclassified	*Cenarchaeales*
Candidatus "Nitrososphaera gargensis"	nga	Unclassified	*Nitrososphaerales*
[***Aigarchaeota***: 1 species]			
Candidatus "Caldiarchaeum subterraneum"	csu	Unclassified	Unclassified
[***Nanoarchaeota***: 1 species]			
Nanoarchaeum equitans	neq	Unclassified	Unclassified
[***Korarchaeota***: 1 species]			
Candidatus "Korarchaeum cryptofilum"	kcr	Unclassified	Unclassified
