# Supplementary material for: Functional Classification of Uncultured “Candidatus Caldiarchaeum subterraneum” Using the Maple System
Source: PLoS One. 2015 Jul 21;10(7):e0132994. doi: 10.1371/journal.pone.0132994 (PMC4510362; doi:10.1371/journal.pone.0132994)
Supplement: S3 Table — (PDF) [file pone.0132994.s006.pdf]

Table S3 Module completion patterns in 79 archaeal species listed in Table S2 (Methanogenic euryarchaeota).

ID	Module name	Module completion ratio (%)																										
		mja	mig	mvn	mok	mba	mbu	mmh	mev	mzh	mpy	mhz	mcj	mhu	mla	mbg	mpi	mbn	mpl	mpd	mer	mth	mst	mru	mel	mfv	mka	max
Pathway module																												
Energy metabolism																												
M00165	Reductive pentose phosphate cycle (Calvin cycle)	54.5	54.5	45.5	54.5	45.5	63.6	63.6	63.6	63.6	45.5	45.5	54.5	54.5	45.5	45.5	72.7	72.7	54.5	54.5	54.5	27.3	27.3	27.3	27.3	27.3	27.3	54.5
M00166	Reductive pentose phosphate cycle, ribulose-5P => glyceraldehyde-3P	75	75	50	75	75	75	75	75	75	75	75	100	100	75	100	100	100	100	75	75	50	50	50	50	50	50	75
M00167	Reductive pentose phosphate cycle, glyceraldehyde-3P => ribulose-5P	42.9	42.9	42.9	42.9	28.6	57.1	57.1	57.1	57.1	28.6	28.6	28.6	28.6	28.6	14.3	57.1	57.1	28.6	42.9	42.9	14.3	14.3	14.3	14.3	14.3	14.3	42.9
M00168	CAM (Crassulacean acid metabolism), dark	50	50	50	50	100	0	50	100	100	50	50	0	100	50	100	100	50	50	0	50	100	50	50	50	50	100	0
M00169	CAM (Crassulacean acid metabolism), light	0	0	0	0	50	50	50	50	50	50	50	0	50	0	0	0	0	0	0	0	0	0	0	0	0	0	0
M00172	C4-dicarboxylic acid cycle, NADP - malic enzyme type	0	0	0	0	50	25	25	50	50	25	25	0	50	0	25	25	0	0	0	25	25	0	0	0	25	25	0
M00171	C4-dicarboxylic acid cycle, NAD - malic enzyme type	0	0	0	0	28.6	14.3	14.3	28.6	28.6	14.3	14.3	0	28.6	0	14.3	14.3	0	0	0	14.3	14.3	0	0	0	14.3	14.3	0
M00170	C4-dicarboxylic acid cycle, phosphoenolpyruvate carboxykinase type	0	0	0	0	25	0	0	25	25	0	0	0	25	0	25	25	0	0	0	25	25	0	0	0	25	25	0
M00173	Reductive citrate cycle (Arnon-Buchanan cycle)	60	60	60	60	72.7	60	80	81.8	72.7	70	70	50	63.6	60	63.6	63.6	60	60	40	54.5	54.5	60	60	70	54.5	63.6	36.4
M00376	3-Hydroxypropionate bi-cycle	6.2	6.2	6.2	6.2	6.2	6.2	12.5	12.5	12.5	6.2	6.2	6.2	6.2	6.2	6.2	6.2	6.2	6.2	6.2	6.2	6.2	6.2	6.2	12.5	6.2	6.2	6.2
M00375	Hydroxypropionate-hydroxybutylate cycle	8.3	8.3	8.3	8.3	8.3	8.3	8.3	8.3	8.3	8.3	8.3	8.3	8.3	8.3	8.3	8.3	8.3	8.3	8.3	8.3	8.3	8.3	8.3	8.3	8.3	8.3	8.3
M00374	Dicarboxylate-hydroxybutyrate cycle	46.2	46.2	46.2	46.2	46.2	30.8	46.2	53.8	53.8	38.5	38.5	30.8	53.8	46.2	53.8	53.8	46.2	46.2	30.8	38.5	46.2	46.2	46.2	53.8	46.2	53.8	30.8
M00377	Reductive acetyl-CoA pathway (Wood-Ljungdahl pathway)	14.3	14.3	0	0	28.6	14.3	14.3	14.3	14.3	28.6	28.6	28.6	28.6	14.3	28.6	28.6	28.6	28.6	0	71.4	0	0	0	0	0	14.3	28.6
M00579	Phosphate acetyltransferase-acetate kinase pathway, acetyl-CoA => acetate	0	0	0	0	100	0	0	0	0	0	0	0	0	0	0	0	0	0	0	0	0	0	0	0	0	0	0
M00175	Nitrogen fixation, nitrogen => ammonia	0	100	100	100	100	0	0	0	0	100	0	100	0	0	0	100	100	100	0	0	100	0	0	100	0	0	0
M00531	Assimilatory nitrate reduction, nitrate => ammonia	0	0	0	0	0	0	0	0	0	0	0	0	0	0	0	0	0	0	0	0	0	0	0	0	0	0	50
M00530	Dissimilatory nitrate reduction, nitrate => ammonia	0	0	0	0	0	0	0	0	0	0	0	0	0	0	0	0	0	0	0	0	0	0	0	0	0	0	0
M00529	Denitrification, nitrate => nitrogen	0	0	0	0	0	0	0	0	0	0	0	0	0	0	0	0	0	0	0	0	0	0	0	0	0	0	0
M00528	Nitrification, ammonia => nitrite	0	0	0	0	0	0	0	0	0	0	0	0	0	0	0	0	0	0	0	0	0	0	0	0	0	0	0
M00567	Methanogenesis, CO2 => methane	75	100	100	100	100	100	100	100	100	100	100	100	100	100	100	100	100	100	100	25	100	100	100	100	100	100	25
M00357	Methanogenesis, acetate => methane	60	80	80	100	100	100	100	100	100	100	100	100	100	80	100	80	100	100	80	60	100	80	80	100	100	80	40
M00356	Methanogenesis, methanol => methane	33.3	66.7	66.7	66.7	100	100	100	100	100	100	100	66.7	66.7	66.7	66.7	66.7	66.7	66.7	66.7	100	66.7	100	66.7	100	66.7	66.7	100
M00563	Methanogenesis, mono-/di-/tri-methylamine => methane	25	50	50	50	100	100	100	100	100	100	100	50	50	50	50	50	50	50	50	75	50	50	50	50	50	50	75
M00358	Coenzyme M biosynthesis	75	100	100	100	0	25	25	0	0	25	25	25	0	0	0	0	0	0	25	25	100	100	50	100	100	100	0
M00174	Methane oxidation, methanotroph, methane => formaldehyde	0	0	0	0	0	0	0	0	0	0	0	0	0	0	0	0	0	0	0	0	0	0	0	0	0	0	0
M00346	Formaldehyde assimilation, serine pathway	33.3	33.3	22.2	22.2	44.4	22.2	33.3	44.4	55.6	44.4	44.4	22.2	55.6	33.3	44.4	44.4	33.3	33.3	33.3	33.3	44.4	33.3	33.3	33.3	33.3	44.4	22.2

ID	Module name	Module completion ratio (%)																										
		mja	mig	mvn	mok	mba	mbu	mmh	mev	mzh	mpy	mhz	mcj	mhu	mla	mbg	mpi	mbn	mpl	mpd	mer	mth	mst	mru	mel	mfv	mka	max
M00345	Formaldehyde assimilation, ribulose monophosphate pathway	50	50	33.3	50	50	75	75	75	75	75	75	33.3	75	50	75	50	100	75	100	33.3	50	50	50	50	50	50	33.3
M00344	Formaldehyde assimilation, xylulose monophosphate pathway	0	0	0	0	0	25	25	25	25	0	0	25	25	25	0	25	50	25	25	0	0	0	0	0	0	0	0
M00378	F420 biosynthesis	100	100	100	100	80	80	80	80	100	100	80	80	60	60	60	80	60	60	80	0	80	80	80	80	80	80	0
M00422	Acetyl-CoA pathway, CO2 => acetyl-CoA	100	100	100	100	100	100	100	100	100	100	100	100	100	0	50	0	100	50	0	0	100	0	0	100	100	100	0
M00176	Assimilatory sulfate reduction, sulfate => H2S	33.3	33.3	33.3	33.3	33.3	33.3	33.3	66.7	33.3	33.3	33.3	33.3	33.3	33.3	33.3	33.3	33.3	33.3	66.7	33.3	0	0	0	0	0	0	33.3
M00596	Dissimilatory sulfate reduction, sulfate => H2S	0	0	0	0	0	0	33.3	33.3	0	0	0	0	0	0	0	0	0	0	33.3	0	0	0	0	0	0	0	0
Carbohydrate & lipid metabolism																												
M00001	Glycolysis (Embden-Meyerhof pathway), glucose => pyruvate	100	90	100	100	100	100	100	100	100	100	100	90	100	80	100	70	90	100	90	70	60	60	70	60	60	60	60
M00002	Glycolysis, core module involving three-carbon compounds	100	83.3	100	100	100	100	100	100	100	100	100	83.3	100	83.3	100	83.3	83.3	100	100	83.3	83.3	83.3	100	83.3	83.3	83.3	83.3
M00003	Gluconeogenesis, oxaloacetate => fructose-6P	62.5	62.5	75	62.5	62.5	62.5	62.5	62.5	62.5	50	50	75	62.5	75	75	62.5	75	75	50	62.5	62.5	62.5	62.5	62.5	62.5	62.5	75
M00307	Pyruvate oxidation, pyruvate => acetyl-CoA	100	100	100	100	100	100	100	100	100	100	100	100	100	100	100	100	100	100	100	100	0	100	100	100	100	100	100
M00009	Citrate cycle (TCA cycle, Krebs cycle)	50	50	50	50	62.5	37.5	50	50	50	62.5	62.5	37.5	50	50	50	50	50	50	25	62.5	62.5	50	62.5	62.5	50	50	37.5
M00010	Citrate cycle, first carbon oxidation, oxaloacetate => 2-oxoglutarate	0	0	0	0	100	66.7	66.7	66.7	66.7	100	100	66.7	0	0	0	0	0	0	33.3	100	33.3	0	33.3	33.3	33.3	0	66.7
M00011	Citrate cycle, second carbon oxidation, 2-oxoglutarate => oxaloacetate	80	80	80	80	40	20	40	40	40	40	40	20	80	80	80	80	80	80	20	40	80	80	80	80	60	80	20
M00004	Pentose phosphate pathway (Pentose phosphate cycle)	71.4	71.4	71.4	71.4	28.6	28.6	28.6	28.6	28.6	28.6	28.6	28.6	28.6	28.6	28.6	57.1	28.6	28.6	42.9	71.4	14.3	14.3	14.3	14.3	14.3	14.3	71.4
M00006	Pentose phosphate pathway, oxidative phase, glucose 6P => ribulose 5P	0	0	0	0	0	0	0	0	0	0	0	0	0	0	0	0	0	0	50	0	0	0	0	0	0	0	0
M00007	Pentose phosphate pathway, non-oxidative phase,fructose 6P => ribose 5P	100	100	100	100	25	25	25	25	25	25	25	25	25	25	25	75	25	25	25	100	25	25	25	25	25	25	100
M00580	Pentose phosphate pathway, archaea, fructose 6P => ribose 5P	100	100	100	100	100	100	100	100	100	100	100	100	100	100	100	100	100	100	100	100	100	100	100	100	100	100	100
M00005	PRPP biosynthesis, ribose 5P => PRPP	100	100	100	100	100	100	100	100	100	100	100	100	100	100	100	100	100	100	100	100	100	100	100	100	100	100	100
M00008	Entner-Doudoroff pathway, glucose-6P => glyceraldehyde-3P + pyruvate	0	0	0	0	0	0	0	0	0	0	0	0	0	0	0	0	0	0	0	0	0	0	0	0	0	0	0
M00308	Semi-phosphorylative Entner-Doudoroff pathway, gluconate => glyceraldehyde-3P + pyruvate	20	20	20	20	20	20	20	20	20	20	20	20	20	20	20	20	20	20	20	20	20	20	20	20	20	25	20
M00633	Semi-phosphorylative Entner-Doudoroff pathway, gluconate/galactonate => glycerate-3P	0	0	0	0	0	0	0	0	0	0	0	0	0	0	0	0	0	0	0	0	0	0	0	0	0	25	0
M00309	Non-phosphorylative Entner-Doudoroff pathway, gluconate => glyceraldehyde + pyruvate	0	0	0	0	33.3	0	0	33.3	33.3	33.3	33.3	0	33.3	33.3	0	0	0	33.3	33.3	0	0	0	0	0	0	0	0
M00012	Glyoxylate cycle	20	20	20	20	60	20	40	40	40	60	60	20	20	20	20	20	20	20	0	40	40	20	40	40	20	20	20
M00373	Ethylmalonyl pathway	7.1	7.1	7.1	7.1	7.1	7.1	7.1	7.1	7.1	7.1	7.1	7.1	7.1	7.1	7.1	7.1	7.1	7.1	7.1	7.1	7.1	7.1	7.1	7.1	7.1	7.1	7.1
M00532	Photorespiration	20	20	0	10	40	30	20	20	30	30	30	30	30	40	20	30	20	20	30	40	10	20	10	10	10	10	40
M00013	Malonate semialdehyde pathway, propanoyl-CoA => Acetyl-CoA	0	0	0	0	0	0	0	0	0	0	0	0	0	0	0	0	0	0	0	0	0	0	0	0	0	0	0
M00632	Galactose degradation, Leloir pathway, galactose => alpha-D-glucose-1P	25	25	25	25	50	50	50	25	25	50	50	50	25	25	25	25	25	25	50	25	25	25	25	25	25	25	50

ID	Module name	Module completion ratio (%)																										
		mja	mig	mvn	mok	mba	mbu	mmh	mev	mzh	mpy	mhz	mcj	mhu	mla	mbg	mpi	mbn	mpl	mpd	mer	mth	mst	mru	mel	mfv	mka	max
M00552	D-galactonate degradation, De Ley-Doudoroff pathway, D-galactonate => glycerate-3P	20	20	20	20	20	20	20	20	20	20	20	20	20	20	20	20	20	20	20	20	20	20	20	20	20	20	20
M00014	Glucuronate pathway (uronate pathway)	14.3	14.3	14.3	14.3	14.3	14.3	14.3	14.3	14.3	14.3	14.3	14.3	14.3	0	0	14.3	14.3	0	14.3	0	0	14.3	14.3	14.3	14.3	0	0
M00631	D-Galacturonate degradation (bacteria), D-galacturonate => pyruvate + D-glyceraldehyde 3P	0	0	0	0	0	0	0	0	0	0	0	0	0	0	0	0	0	0	0	0	0	0	0	0	0	0	0
M00061	Uronic acid metabolism	0	0	0	0	0	0	0	0	0	0	0	0	0	0	0	0	0	0	0	0	0	0	0	0	0	0	0
M00081	Pectin degradation	0	0	0	0	0	0	0	0	0	0	0	0	0	0	0	0	0	0	0	0	0	0	0	0	0	0	0
M00082	Fatty acid biosynthesis, initiation	0	0	0	0	0	0	0	0	0	0	0	0	0	0	0	0	0	0	0	0	0	0	0	0	0	0	0
M00113	Jasmonic acid biosynthesis	0	0	0	0	0	0	0	0	0	0	0	0	0	0	0	0	0	0	0	0	0	0	0	0	0	0	0
M00086	beta-Oxidation, acyl-CoA synthesis	0	0	0	0	0	0	0	0	0	0	0	0	100	100	100	100	100	100	0	0	0	100	100	100	0	0	0
M00087	beta-Oxidation	0	0	0	0	0	0	0	0	0	0	0	0	0	0	0	0	0	0	0	0	0	0	0	0	0	0	0
M00088	Ketone body biosynthesis, acetyl-CoA => acetoacetate/3-hydroxybutyrate/acetone	40	40	40	40	40	40	40	40	40	40	40	60	40	40	40	40	40	40	40	40	40	40	40	40	40	40	40
M00093	Phosphatidylethanolamine (PE) biosynthesis, PA => PS => PE	66.7	66.7	33.3	66.7	66.7	66.7	66.7	66.7	66.7	66.7	66.7	66.7	0	0	0	0	0	0	0	0	66.7	33.3	33.3	66.7	0	66.7	0
M00131	Inositol phosphate metabolism, Ins(1,3,4,5)P4 => Ins(1,3,4)P3 => myo-inositol	25	25	0	25	25	25	25	25	25	25	25	25	25	25	25	25	25	25	25	25	25	25	0	25	25	25	25
M00094	Ceramide biosynthesis	0	0	0	0	0	0	0	0	0	0	0	0	0	0	0	0	0	0	0	0	0	0	0	0	0	0	0
M00099	Sphingosine biosynthesis	0	0	0	0	0	0	0	0	0	0	0	0	0	0	0	0	0	0	0	0	0	0	0	0	0	0	0
M00060	Lipopolysaccharide biosynthesis, KDO2-lipid A	0	0	0	0	0	0	0	0	0	0	0	0	0	0	0	0	11.1	0	0	0	0	0	0	0	0	0	0
M00064	ADP-L-glycero-D-manno-heptose biosynthesis	25	75	0	75	0	0	0	0	0	0	0	0	0	0	0	50	0	0	0	25	0	0	0	0	0	0	50
M00080	Lipopolysaccharide biosynthesis, inner core => outer core => O-antigen	0	8.3	0	8.3	0	0	0	0	0	0	0	0	0	0	0	0	0	0	0	0	0	0	0	0	0	0	0
M00549	Nucleotide sugar biosynthesis, glucose => UDP-glucose	33.3	33.3	33.3	33.3	33.3	33.3	33.3	0	33.3	33.3	33.3	33.3	66.7	66.7	33.3	0	33.3	33.3	33.3	0	33.3	33.3	33.3	33.3	33.3	0	0
M00554	Nucleotide sugar biosynthesis, galactose => UDP-galactose	0	0	0	0	50	50	50	0	50	50	50	50	0	0	0	0	0	0	50	0	0	0	0	0	0	0	0
M00565	Trehalose biosynthesis, D-glucose 1P => trehalose	40	40	40	40	40	0	0	20	20	20	20	20	20	0	40	0	20	0	0	0	0	0	0	0	0	0	0
M00055	N-glycan precursor biosynthesis	7.7	7.7	7.7	7.7	0	7.7	0	0	0	7.7	0	0	0	0	0	0	0	0	0	0	7.7	7.7	7.7	0	7.7	0	0
M00076	Dermatan sulfate degradation	0	0	0	0	0	0	0	0	0	0	0	0	0	0	0	0	0	0	0	0	0	0	0	0	0	0	0
M00077	Chondroitin sulfate degradation	0	0	0	0	0	0	0	0	0	0	0	0	0	0	0	0	0	0	0	0	0	0	0	0	0	0	0
M00078	Heparan sulfate degradation	0	0	0	0	0	0	0	0	0	0	0	0	0	0	0	0	0	0	0	0	0	0	0	0	0	0	0
M00095	C5 isoprenoid biosynthesis, mevalonate pathway	71.4	71.4	71.4	71.4	71.4	71.4	71.4	71.4	71.4	71.4	71.4	57.1	71.4	71.4	71.4	71.4	71.4	71.4	71.4	71.4	71.4	71.4	71.4	71.4	71.4	71.4	71.4
M00096	C5 isoprenoid biosynthesis, non-mevalonate pathway	12.5	12.5	12.5	12.5	12.5	12.5	12.5	12.5	12.5	12.5	12.5	12.5	12.5	12.5	12.5	12.5	12.5	12.5	12.5	12.5	12.5	12.5	25	12.5	12.5	12.5	25
M00364	C10-C20 isoprenoid biosynthesis, bacteria	100	100	100	100	100	100	100	50	100	100	100	100	100	100	100	100	100	100	100	100	100	100	100	100	100	100	100
M00365	C10-C20 isoprenoid biosynthesis, archaea	100	100	100	100	100	100	100	50	100	100	100	100	100	100	100	100	100	100	100	100	100	100	100	100	100	100	100

ID	Module name	Module completion ratio (%)																										
		mja	mig	mvn	mok	mba	mbu	mmh	mev	mzh	mpy	mhz	mcj	mhu	mla	mbg	mpi	mbn	mpl	mpd	mer	mth	mst	mru	mel	mfv	mka	max
M00366	C10-C20 isoprenoid biosynthesis, plants	25	25	25	25	25	25	25	25	25	25	25	25	25	25	25	25	25	25	25	25	25	25	25	25	25	25	25
M00367	C10-C20 isoprenoid biosynthesis, non-plant eukaryotes	33.3	33.3	33.3	33.3	33.3	33.3	33.3	33.3	33.3	33.3	33.3	33.3	33.3	33.3	33.3	33.3	33.3	33.3	33.3	33.3	33.3	33.3	33.3	33.3	33.3	33.3	33.3
M00097	beta-Carotene biosynthesis, GGAP => beta-carotene	0	0	0	0	0	0	0	0	16.7	0	0	0	0	0	0	0	0	0	0	0	16.7	0	0	0	0	0	0
M00372	Abscisic acid biosynthesis, beta-carotene => abscisic acid	0	0	0	0	0	0	0	0	0	0	0	0	0	0	0	0	0	0	0	0	0	0	0	0	0	0	0
Nucleotide & amino acid metabolism																												
M00048	Inosine monophosphate biosynthesis, PRPP + glutamine => IMP	90.9	90.9	90.9	90.9	90	90	90	90	90	90	90	90.9	80	90	90	90	90	80	90.9	80	81.8	81.8	81.8	81.8	81.8	81.8	80
M00049	Adenine ribonucleotide biosynthesis, IMP => ADP, ATP	100	100	100	100	100	100	100	100	100	100	100	100	100	100	100	100	100	100	100	100	100	100	100	100	100	100	100
M00050	Guanine ribonucleotide biosynthesis IMP => GDP, GTP	75	75	75	75	50	50	50	50	50	50	50	50	75	75	75	75	75	75	75	75	75	75	75	75	75	75	50
M00546	Purine degradation, xanthine => urea	0	0	0	0	0	0	0	0	0	0	0	0	0	0	0	0	0	0	0	0	0	0	0	0	0	0	0
M00051	Uridine monophosphate biosynthesis, glutamine (+ PRPP) => UMP	100	100	100	100	100	100	100	100	100	100	100	100	100	100	100	100	83.3	100	100	100	100	100	100	100	100	100	100
M00052	Pyrimidine ribonucleotide biosynthesis, UMP => UDP/UTP, CDP/CTP	100	100	100	100	100	100	100	100	100	100	100	100	100	100	100	100	100	100	100	100	100	100	100	100	100	100	66.7
M00053	Pyrimidine deoxyribonuleotide biosynthesis, CDP/CTP => dCDP/dCTP, dTDP/dTTP	62.5	87.5	87.5	87.5	75	75	75	75	75	75	75	75	75	62.5	75	75	75	75	75	62.5	87.5	75	87.5	87.5	87.5	87.5	50
M00046	Pyrimidine degradation, uracil => beta-alanine, thymine => 3-aminoisobutanoate	0	0	0	0	0	0	0	0	0	0	0	0	0	0	0	0	0	0	0	0	0	0	0	0	0	0	0
M00020	Serine biosynthesis, glycerate-3P => serine	66.7	66.7	66.7	66.7	100	66.7	66.7	66.7	66.7	66.7	66.7	66.7	33.3	33.3	33.3	33.3	33.3	33.3	66.7	66.7	66.7	66.7	66.7	66.7	66.7	66.7	66.7
M00018	Threonine biosynthesis, aspartate => homoserine => threonine	100	100	100	100	80	80	80	80	80	80	80	80	80	80	80	80	80	80	100	100	80	80	80	80	80	100	100
M00555	Betaine biosynthesis, choline => betaine	0	0	0	0	0	0	0	0	0	0	0	0	0	0	0	0	0	0	0	0	0	0	0	0	0	0	0
M00021	Cysteine biosynthesis, serine => cysteine	0	0	100	0	100	100	0	100	100	100	100	0	100	0	0	0	100	100	0	100	0	100	100	100	0	0	100
M00338	Cysteine biosynthesis, homocysteine + serine => cysteine	0	0	0	0	0	0	0	0	0	0	0	0	0	0	0	0	100	100	0	0	0	0	0	0	0	0	0
M00609	Cysteine biosynthesis, methionine => cysteine	16.7	16.7	16.7	16.7	16.7	16.7	16.7	16.7	16.7	16.7	16.7	16.7	16.7	16.7	16.7	16.7	16.7	16.7	16.7	16.7	16.7	16.7	16.7	16.7	16.7	16.7	16.7
M00017	Methionine biosynthesis, apartate => homoserine => methionine	57.1	57.1	57.1	57.1	57.1	57.1	57.1	57.1	57.1	57.1	57.1	57.1	57.1	57.1	57.1	57.1	57.1	57.1	57.1	57.1	57.1	57.1	57.1	71.4	57.1	57.1	71.4
M00034	Methionine salvage pathway	50	62.5	62.5	62.5	37.5	37.5	37.5	37.5	37.5	37.5	37.5	37.5	50	37.5	37.5	37.5	37.5	37.5	50	37.5	12.5	25	37.5	37.5	37.5	37.5	37.5
M00035	Methionine degradation	75	75	75	50	50	75	50	50	50	50	75	50	50	75	50	75	75	75	50	75	75	50	75	50	50	75	50
M00368	Ethylene biosynthesis, methionine => ethylene	33.3	33.3	33.3	33.3	33.3	33.3	33.3	33.3	33.3	33.3	33.3	33.3	33.3	33.3	33.3	33.3	33.3	33.3	33.3	33.3	33.3	33.3	33.3	33.3	33.3	33.3	33.3
M00019	Valine/isoleucine biosynthesis, pyruvate => valine / 2-oxobutanoate => isoleucine	100	100	100	100	100	100	100	100	100	100	100	100	100	100	100	100	100	100	100	100	100	100	100	100	100	100	75
M00535	Isoleucine biosynthesis, pyruvate => 2-oxobutanoate	100	100	100	100	100	100	100	100	100	100	100	100	100	100	100	100	100	100	100	66.7	100	100	100	100	100	100	66.7
M00570	Isoleucine biosynthesis, threonine => 2-oxobutanoate => isoleucine	80	80	80	80	80	80	80	80	80	80	80	100	100	80	80	80	80	80	80	80	80	80	80	80	80	80	60
M00432	Leucine biosynthesis, 2-oxoisovalerate => 2-oxoisocaproate	100	100	100	100	100	100	100	100	100	100	100	100	100	100	100	100	100	100	100	100	100	100	100	100	100	100	100
M00036	Leucine degradation, leucine => acetoacetate + acetyl-CoA	16.7	16.7	16.7	16.7	16.7	16.7	16.7	16.7	16.7	16.7	16.7	16.7	16.7	16.7	16.7	16.7	16.7	16.7	16.7	16.7	16.7	16.7	16.7	16.7	16.7	16.7	16.7

ID	Module name	Module completion ratio (%)																										
		mja	mig	mvn	mok	mba	mbu	mmh	mev	mzh	mpy	mhz	mcj	mhu	mla	mbg	mpi	mbn	mpl	mpd	mer	mth	mst	mru	mel	mfv	mka	max
M00016	Lysine biosynthesis, succinyl-DAP pathway, aspartate => lysine	88.9	88.9	88.9	88.9	66.7	66.7	66.7	77.8	77.8	66.7	66.7	88.9	88.9	88.9	88.9	88.9	88.9	88.9	77.8	88.9	77.8	77.8	77.8	77.8	77.8	88.9	77.8
M00525	Lysine biosynthesis, acetyl-DAP pathway, aspartate => lysine	66.7	66.7	66.7	66.7	55.6	55.6	55.6	66.7	66.7	55.6	55.6	66.7	66.7	66.7	66.7	66.7	66.7	66.7	66.7	66.7	66.7	66.7	66.7	66.7	66.7	66.7	66.7
M00526	Lysine biosynthesis, DAP dehydrogenase pathway, aspartate => lysine	83.3	83.3	100	83.3	83.3	83.3	83.3	83.3	83.3	83.3	83.3	83.3	83.3	83.3	83.3	83.3	83.3	83.3	83.3	83.3	83.3	83.3	83.3	83.3	83.3	83.3	83.3
M00527	Lysine biosynthesis, DAP aminotransferase pathway, aspartate => lysine	100	100	100	100	85.7	85.7	85.7	100	100	85.7	85.7	100	100	100	100	100	100	100	100	100	100	100	100	100	100	85.7	100
M00030	Lysine biosynthesis, AAA pathway, 2-oxoglutarate => 2-aminoadipate => lysine	0	0	0	0	0	0	0	0	0	0	0	0	0	0	0	12.5	0	0	0	0	0	0	0	0	0	0	0
M00433	Lysine biosynthesis, 2-oxoglutarate => 2-oxoadipate	100	100	100	100	100	66.7	100	100	100	100	100	100	100	100	66.7	100	100	100	100	0	100	100	100	100	100	100	0
M00031	Lysine biosynthesis, 2-aminoadipate => lysine	0	0	0	0	0	0	0	0	0	0	0	0	0	0	0	0	0	0	0	0	0	0	0	0	0	0	0
M00032	Lysine degradation, lysine => saccharopine => acetoacetyl-CoA	0	0	0	0	0	0	0	0	0	0	0	0	0	0	0	12.5	0	0	0	0	0	0	0	0	0	0	0
M00608	2-oxocarboxylic acid chain extension, 2-oxoglutarate => 2-oxoadipate => 2-oxopimelate => 2-oxosuberate	100	100	100	100	100	66.7	100	100	100	100	100	100	100	100	66.7	100	100	100	100	0	100	100	100	100	100	100	0
M00015	Proline biosynthesis, glutamate => proline	0	0	0	0	100	100	100	100	100	100	100	0	0	0	50	50	0	0	0	100	0	50	50	50	0	0	100
M00028	Ornithine biosynthesis, glutamate => ornithine	100	100	100	100	100	100	100	100	100	100	100	100	100	100	100	100	100	100	100	100	100	100	100	100	100	100	100
M00029	Urea cycle	60	60	60	60	60	60	60	60	60	60	60	60	60	60	60	60	60	60	80	60	60	60	60	60	60	60	60
M00026	Histidine biosynthesis, PRPP => histidine	88.9	88.9	88.9	88.9	88.9	88.9	88.9	88.9	88.9	88.9	88.9	77.8	88.9	88.9	88.9	88.9	88.9	88.9	88.9	87.5	88.9	88.9	88.9	88.9	88.9	88.9	87.5
M00045	Histidine degradation, histidine => N-formiminoglutamate => glutamate	0	0	0	0	0	0	0	0	0	0	0	0	0	0	0	0	0	0	0	0	0	0	0	0	0	0	0
M00022	Shikimate pathway, phosphoenolpyruvate + erythrose-4P => chorismate	71.4	71.4	71.4	71.4	71.4	71.4	71.4	71.4	71.4	71.4	71.4	71.4	71.4	60	42.9	42.9	60	42.9	71.4	66.7	60	71.4	71.4	71.4	71.4	71.4	66.7
M00023	Tryptophan biosynthesis, chorismate => tryptophan	100	100	100	100	100	100	100	100	100	100	100	100	100	100	100	100	100	100	100	25	75	100	100	100	100	100	100
M00024	Phenylalanine biosynthesis, chorismate => phenylalanine	66.7	66.7	66.7	66.7	66.7	66.7	66.7	66.7	66.7	66.7	66.7	66.7	66.7	33.3	66.7	66.7	66.7	66.7	66.7	66.7	66.7	66.7	66.7	66.7	66.7	66.7	66.7
M00025	Tyrosine biosynthesis, chorismate => tyrosine	66.7	66.7	66.7	66.7	66.7	66.7	66.7	66.7	66.7	66.7	66.7	66.7	66.7	33.3	66.7	66.7	66.7	66.7	66.7	50	66.7	66.7	66.7	66.7	66.7	33.3	66.7
M00042	Catecholamine biosynthesis, tyrosine => dopamine => noradrenaline => adrenaline	25	25	25	25	25	25	25	25	25	25	25	25	25	25	25	25	25	25	25	0	25	25	25	25	25	25	0
M00044	Tyrosine degradation, tyrosine => homogentisate	0	0	0	0	0	0	0	0	0	0	0	0	0	0	0	0	0	0	0	0	0	0	0	0	0	0	0
M00038	Tryptophan metabolism, tryptophan => kynurenine => 2-aminomuconate	0	0	0	0	14.3	0	0	14.3	0	14.3	14.3	14.3	0	0	14.3	14.3	14.3	14.3	14.3	0	0	0	0	0	0	0	0
M00027	GABA (gamma-Aminobutyrate) shunt	0	0	0	0	100	33.3	33.3	33.3	33.3	33.3	33.3	33.3	33.3	0	0	66.7	0	0	0	33.3	0	0	0	33.3	0	0	0
M00127	Thiamine biosynthesis, AIR => thiamine-P/thiamine-2P	60	60	100	60	100	60	100	60	60	100	100	100	100	100	100	100	80	100	100	100	60	100	100	60	60	60	100
M00125	Riboflavin biosynthesis, GTP => riboflavin/FMN/FAD	50	50	50	50	50	50	50	50	50	50	50	66.7	50	33.3	50	50	50	50	50	50	50	50	50	50	50	50	50
M00124	Pyridoxal biosynthesis, erythrose-4P => pyridoxal-5P	0	0	0	0	16.7	0	0	0	0	0	0	0	0	0	0	0	0	0	0	0	0	0	0	0	0	0	0
M00115	NAD biosynthesis, aspartate => NAD	60	60	60	60	60	60	60	60	60	60	60	60	60	60	60	60	60	60	60	60	60	60	60	60	60	60	60
M00622	Nicotinate degradation, nicotinate => fumarate	0	0	0	0	0	0	0	0	0	0	0	0	0	0	0	0	0	0	0	0	0	0	0	0	0	0	0
M00119	Pantothenate biosynthesis, valine/L-aspartate => pantothenate	20	20	40	20	20	20	20	20	20	20	40	40	20	40	40	40	40	20	40	60	20	40	40	40	20	20	40

ID	Module name	Module completion ratio (%)																										
		mja	mig	mvn	mok	mba	mbu	mmh	mev	mzh	mpy	mhz	mcj	mhu	mla	mbg	mpi	mbn	mpl	mpd	mer	mth	mst	mru	mel	mfv	mka	max
M00120	Coenzyme A biosynthesis, pantothenate => CoA	50	50	50	50	50	50	33.3	50	50	50	50	50	50	50	50	50	50	50	50	50	50	50	50	50	33.3	50	33.3
M00572	Pimeloyl-ACP biosynthesis, BioC-BioH pathway, malonyl-ACP => pimeloyl-ACP	0	0	0	0	16.7	0	0	0	0	0	16.7	0	16.7	0	0	0	16.7	0	0	0	0	0	16.7	0	0	0	16.7
M00123	Biotin biosynthesis, pimeloyl-ACP/CoA => biotin	100	100	100	100	0	0	0	0	25	0	0	0	0	25	0	25	0	0	0	25	25	0	75	0	25	25	25
M00573	Biotin biosynthesis, BioI pathway, long-chain-acyl-ACP => pimeloyl-ACP => biotin	80	80	80	80	0	0	0	0	20	0	0	0	0	20	0	20	0	0	0	20	20	0	60	0	20	20	20
M00577	Biotin biosynthesis, BioW pathway, pimelate => pimeloyl-CoA => biotin	100	100	100	100	0	0	0	0	20	0	0	0	0	20	0	20	0	0	0	20	20	0	80	0	20	20	20
M00126	Tetrahydrofolate biosynthesis, GTP => THF	0	0	0	0	33.3	16.7	16.7	16.7	16.7	16.7	16.7	33.3	16.7	16.7	16.7	16.7	16.7	16.7	0	33.3	0	0	0	0	0	0	33.3
M00121	Heme biosynthesis, glutamate => protoheme/siroheme	54.5	63.6	63.6	54.5	54.5	63.6	63.6	63.6	63.6	54.5	63.6	54.5	54.5	45.5	72.7	63.6	54.5	72.7	63.6	63.6	54.5	54.5	63.6	63.6	54.5	54.5	72.7
M00129	Ascorbate biosynthesis, animals, glucose-1P => ascorbate	33.3	33.3	33.3	33.3	33.3	33.3	33.3	16.7	33.3	16.7	33.3	16.7	33.3	16.7	0	16.7	33.3	0	16.7	0	16.7	33.3	33.3	33.3	33.3	0	0
M00114	Ascorbate biosynthesis, plants, glucose-6P => ascorbate	22.2	44.4	22.2	44.4	44.4	44.4	44.4	44.4	44.4	44.4	44.4	22.2	44.4	33.3	44.4	44.4	44.4	44.4	33.3	37.5	25	12.5	12.5	12.5	12.5	25	37.5
M00550	Ascorbate degradation, ascorbate => D-xylulose-5P	0	0	0	0	0	0	0	0	0	0	0	0	0	0	0	0	0	0	0	0	0	0	0	0	0	0	0
M00122	Cobalamin biosynthesis, cobinamide => cobalamin	37.5	37.5	37.5	37.5	50	37.5	37.5	37.5	37.5	37.5	37.5	37.5	50	25	50	50	37.5	37.5	25	37.5	37.5	37.5	37.5	37.5	37.5	37.5	50
M00117	Ubiquinone biosynthesis, prokaryotes, chorismate => ubiquinone	11.1	11.1	11.1	11.1	44.4	44.4	44.4	33.3	44.4	33.3	44.4	22.2	11.1	22.2	22.2	22.2	22.2	11.1	11.1	22.2	22.2	22.2	22.2	22.2	11.1	11.1	22.2
M00116	Menaquinone biosynthesis, chorismate => menaquinone	0	0	0	0	11.1	11.1	11.1	0	22.2	11.1	11.1	0	0	0	0	0	0	0	11.1	22.2	0	0	0	11.1	0	0	22.2
M00118	Glutathione biosynthesis, glutamate => glutathione	0	0	0	0	0	0	0	0	0	0	0	0	0	0	0	0	0	0	0	0	0	50	50	50	0	0	0
M00140	C1-unit interconversion, prokaryotes	33.3	33.3	0	0	66.7	66.7	66.7	66.7	66.7	66.7	66.7	66.7	66.7	66.7	66.7	66.7	66.7	66.7	33.3	100	33.3	33.3	33.3	33.3	33.3	33.3	100
M00141	C1-unit interconversion, eukaryotes	50	50	0	0	50	50	50	50	50	50	50	50	50	50	50	50	50	50	50	50	50	50	50	50	50	50	50
M00133	Polyamine biosynthesis, arginine => agmatine => putrescine => spermidine	100	100	100	100	50	50	50	50	50	50	50	25	50	50	50	50	50	50	75	25	50	50	50	50	50	75	50
M00134	Polyamine biosynthesis, arginine => ornithine => putrescine	0	0	0	0	50	0	50	0	0	50	50	0	0	0	0	0	50	50	50	0	0	0	0	0	0	0	0
M00135	GABA biosynthesis, eukaryotes, putrescine => GABA	0	0	0	0	33.3	0	0	0	33.3	0	33.3	0	0	0	0	0	0	0	0	33.3	0	0	0	0	0	0	33.3
M00039	Lignin biosynthesis, cinnamate => lignin	0	0	0	0	0	0	20	0	0	20	0	0	20	0	0	0	20	20	0	0	0	0	0	0	0	0	0
M00033	Ectoine biosynthesis, aspartate => ectoine	40	40	40	40	40	40	40	40	40	40	40	100	40	60	40	40	40	40	40	40	40	40	40	40	40	40	40
Secondary metabolism																												
M00569	Catechol meta-cleavage, catechol => pyruvate + acetaldehyde	0	0	16.7	0	0	0	0	0	0	0	0	0	0	16.7	0	0	16.7	16.7	16.7	0	16.7	16.7	16.7	16.7	0	0	0
M00541	Benzoyl-CoA degradation, benzoyl-CoA => 3-hydroxypimeloyl-CoA	0	0	0	0	0	0	0	0	0	0	0	0	0	0	0	0	0	0	0	0	0	0	0	0	0	0	0
M00540	Benzoate degradation, cyclohexanecarboxylic acid => pimeloyl-CoA	0	0	0	0	0	0	0	0	0	0	0	0	0	0	0	0	0	0	0	0	0	0	0	0	0	0	0
M00623	Phthalate degradation, phthalate => protocatechuate	0	0	0	0	0	0	0	0	0	0	0	0	0	0	0	0	0	0	0	0	0	0	0	0	0	0	0

ID	Module name	Module completion ratio (%)																										
		mja	mig	mvn	mok	mba	mbu	mmh	mev	mzh	mpy	mhz	mcj	mhu	mla	mbg	mpi	mbn	mpl	mpd	mer	mth	mst	mru	mel	mfv	mka	max
Structural complex																												
Energy metabolism																												
M00163	Photosystem I	0	0	0	0	0	0	0	0	0	0	0	0	0	0	0	0	0	0	0	0	0	0	0	0	0	0	0
M00144	NADH:quinone oxidoreductase, prokaryotes	0	0	0	0	78.6	71.4	78.6	78.6	71.4	78.6	71.4	76.9	9.1	0	0	0	0	18.2	9.1	64.3	18.2	0	0	18.2	25	0	64.3
M00145	NAD(P)H:quinone oxidoreductase, chloroplasts and cyanobacteria	0	0	0	0	0	0	0	0	0	0	0	0	0	0	0	0	0	0	0	9.1	0	0	0	0	0	0	0
M00149	Succinate dehydrogenase, prokaryotes	0	0	0	0	0	25	50	50	50	0	0	0	0	0	0	0	0	0	0	0	0	0	0	50	0	0	0
M00150	Fumarate reductase, prokaryotes	0	0	0	0	0	0	0	0	0	0	0	0	0	0	0	0	0	0	0	0	0	0	0	0	0	0	0
M00162	Cytochrome b6f complex	0	0	0	0	0	0	0	0	0	0	0	0	0	0	0	0	0	0	0	0	0	0	0	0	0	0	0
M00151	Cytochrome bc1 complex respiratory unit	0	0	0	0	0	0	0	0	0	0	0	0	0	0	0	0	0	0	0	0	0	0	0	0	0	0	0
M00152	Cytochrome bc1 complex	0	0	0	0	0	0	0	0	0	0	0	0	0	0	0	0	0	0	0	0	0	0	0	0	0	0	0
M00154	Cytochrome c oxidase	0	0	0	0	0	0	0	0	0	0	0	0	0	0	0	0	0	0	0	0	0	0	0	0	0	0	0
M00155	Cytochrome c oxidase, prokaryotes	0	0	0	0	0	0	0	0	0	0	0	0	0	0	0	0	0	0	0	0	0	0	0	0	0	0	0
M00153	Cytochrome d ubiquinol oxidase	0	0	0	0	100	0	0	0	0	0	0	0	0	0	0	0	0	0	0	0	0	0	0	0	0	0	0
M00416	Cytochrome aa3-600 menaquinol oxidase	0	0	0	0	0	0	0	0	0	0	0	0	0	0	0	0	0	0	0	0	0	0	0	0	0	0	0
M00157	F-type ATPase, prokaryotes and chloroplasts	0	0	0	0	87.5	0	0	0	0	0	0	0	0	0	0	0	0	0	0	0	0	0	0	0	0	0	0
M00159	V-type ATPase, prokaryotes	100	100	100	87.5	100	100	100	100	100	100	100	100	100	100	100	100	100	100	100	100	100	100	100	100	100	100	100
Carbohydrate & lipid metabolism																												
M00310	Pyruvate:ferredoxin oxidoreductase	100	100	100	100	100	100	100	100	100	100	100	100	100	100	100	100	100	100	100	100	75	100	100	100	100	100	100
M00311	2-oxoglutarate:ferredoxin oxidoreductase	100	100	100	100	50	50	50	50	50	50	50	50	100	100	100	100	100	100	0	100	100	100	100	100	100	100	0
M00312	2-oxoisovalerate:ferredoxin oxidoreductase	0	0	75	0	75	75	75	0	75	75	75	25	0	0	0	0	0	0	0	50	75	0	75	75	75	0	0
M00313	indolepyruvate:ferredoxin oxidoreductase	0	0	100	0	100	100	100	100	100	100	100	100	100	100	100	100	100	100	100	100	100	100	100	100	0	0	100
M00072	Oligosaccharyltransferase	16.7	16.7	16.7	16.7	16.7	16.7	16.7	16.7	16.7	16.7	16.7	16.7	16.7	16.7	16.7	16.7	16.7	16.7	16.7	16.7	16.7	16.7	16.7	16.7	16.7	0	0
Genetic information processing																												
M00260	DNA polymerase III complex, bacteria	0	0	0	0	0	0	0	0	16.7	0	0	0	0	16.7	0	0	0	16.7	16.7	0	0	0	16.7	0	0	0	16.7
M00264	DNA polymerase II complex, archaea	100	100	100	100	100	50	100	100	100	100	100	100	100	100	100	100	100	100	100	100	100	100	100	100	100	100	100
M00261	DNA polymerase alpha / primase complex	25	25	25	25	25	25	25	25	25	25	25	25	25	25	25	25	25	25	25	25	25	25	25	25	25	25	25
M00288	RPA complex	33.3	33.3	33.3	33.3	33.3	33.3	33.3	33.3	33.3	33.3	33.3	33.3	33.3	33.3	33.3	33.3	33.3	33.3	33.3	33.3	33.3	33.3	33.3	33.3	33.3	33.3	33.3
M00290	Holo-TFIIH complex	0	0	0	0	10	10	10	10	10	10	10	10	10	10	10	10	10	10	10	10	0	0	0	0	0	0	10

ID	Module name	Module completion ratio (%)																										
		mja	mig	mvn	mok	mba	mbu	mmh	mev	mzh	mpy	mhz	mcj	mhu	mla	mbg	mpi	mbn	mpl	mpd	mer	mth	mst	mru	mel	mfv	mka	max
M00413	FA core complex	10	10	10	10	10	10	10	10	10	10	10	10	10	10	10	10	10	10	10	10	10	10	10	10	10	10	10
M00184	RNA polymerase, archaea	92.3	100	100	100	100	100	100	100	100	100	100	100	100	100	76.9	100	100	100	100	100	100	100	100	100	100	100	100
M00180	RNA polymerase II, eukaryotes	0	0	0	0	0	0	0	0	0	0	0	0	0	0	0	0	0	0	0	0	0	0	0	0	0	0	0
M00181	RNA polymerase III, eukaryotes	0	0	0	0	0	0	0	0	0	0	0	0	0	0	0	0	0	0	0	0	0	0	0	0	0	0	0
M00182	RNA polymerase I, eukaryotes	0	0	0	0	0	0	0	0	0	0	0	0	0	0	0	0	0	0	0	0	0	0	0	0	0	0	0
M00425	H/ACA ribonucleoprotein complex	50	50	50	50	50	50	50	50	50	25	50	25	25	25	25	25	25	25	50	50	50	50	50	50	50	50	0
M00390	Exosome, archaea	0	0	0	0	100	100	100	100	100	100	100	100	0	0	0	0	0	0	100	100	100	100	100	100	100	100	100
M00391	Exosome, eukaryotes	0	0	0	0	40	40	40	30	40	40	40	40	0	0	0	0	0	0	40	40	40	40	40	30	40	40	40
M00392	Ski complex	0	0	0	0	33.3	0	0	0	0	0	0	0	0	0	0	0	0	0	0	0	0	0	0	0	0	0	0
M00394	RNA degradosome	25	25	25	25	25	25	25	25	25	25	25	25	25	25	25	25	25	25	25	25	25	25	25	25	25	25	25
M00178	Ribosome, bacteria	59.6	59.6	59.6	59.6	59.6	59.6	59.6	59.6	59.6	59.6	59.6	59.6	59.6	59.6	59.6	59.6	59.6	59.6	59.6	59.6	59.6	59.6	59.6	59.6	59.6	59.6	59.6
M00179	Ribosome, archaea	100	100	100	100	100	100	96.6	100	100	98.3	100	98.3	98.3	96.6	98.3	100	100	100	100	98.3	100	98.3	100	100	100	100	100
M00177	Ribosome, eukaryotes	36.7	35.4	35.4	35.4	32.9	32.9	30.4	32.9	32.9	31.6	32.9	31.6	32.9	31.6	31.6	32.9	32.9	32.9	32.9	31.6	35.4	34.2	35.4	35.4	35.4	36.7	32.9
M00341	Proteasome, 19S regulatory particle (PA700)	0	0	0	0	0	0	0	0	0	0	0	0	0	0	0	0	0	0	0	0	0	0	0	0	0	0	0
M00342	Bacterial proteasome	50	50	50	50	50	50	50	50	50	50	50	25	50	50	50	50	50	50	50	50	50	50	50	50	50	50	50
M00343	Archaeal proteasome	100	100	100	100	100	100	100	100	100	100	100	66.7	100	100	100	100	100	100	100	100	100	100	100	100	100	100	100
M00401	Sec61 complex	33.3	33.3	33.3	33.3	33.3	33.3	33.3	33.3	33.3	33.3	33.3	33.3	33.3	33.3	33.3	33.3	33.3	33.3	33.3	33.3	33.3	33.3	33.3	33.3	33.3	33.3	33.3
Environmental information processing																												
M00189	Molybdate transport system	0	100	100	100	100	100	100	0	0	100	100	100	100	0	100	100	100	100	100	0	100	0	100	0	66.7	100	0
M00423	Molybdate/tungstate transport system	100	100	100	100	0	0	0	100	0	0	0	0	0	100	100	33.3	0	0	0	0	0	0	0	100	100	0	0
M00186	Tungstate transport system	0	0	0	0	0	0	0	0	100	0	0	0	100	100	100	0	0	100	100	0	100	0	0	0	0	0	0
M00188	NitT/TauT family transport system	66.7	100	100	100	100	100	100	100	100	100	100	100	100	66.7	100	100	100	100	100	100	0	100	100	100	100	100	66.7
M00190	Iron(III) transport system	0	0	0	0	0	0	0	0	0	0	0	0	0	0	0	0	0	0	0	33.3	0	0	0	0	0	0	0
M00191	Thiamine transport system	0	0	0	0	0	0	0	0	0	0	0	0	0	0	0	0	0	0	0	0	0	0	0	0	0	0	33.3
M00299	Spermidine/putrescine transport system	0	0	0	0	0	0	0	0	0	0	0	0	0	0	0	0	0	0	0	0	0	0	0	0	0	0	0
M00193	Putative spermidine/putrescine transport system	0	0	0	0	0	0	0	0	0	0	0	0	0	0	0	0	0	0	0	50	0	0	0	0	0	0	0
M00208	Glycine betaine/proline transport system	0	0	100	0	100	100	100	100	100	0	0	0	0	100	100	100	0	0	0	0	0	0	0	0	0	0	0
M00209	Osmoprotectant transport system	0	0	0	0	0	100	0	100	0	0	100	0	0	0	0	0	0	100	0	0	0	0	0	0	0	0	0

ID	Module name	Module completion ratio (%)																										
		mja	mig	mvn	mok	mba	mbu	mmh	mev	mzh	mpy	mhz	mcj	mhu	mla	mbg	mpi	mbn	mpl	mpd	mer	mth	mst	mru	mel	mfv	mka	max
M00194	Maltose/maltodextrin transport system	0	0	0	0	0	0	0	0	0	0	0	0	0	0	0	0	0	0	0	0	0	0	0	0	0	0	0
M00196	Multiple sugar transport system	0	0	0	0	0	0	0	0	0	0	0	0	0	0	0	0	0	0	0	0	0	0	0	0	0	0	0
M00203	Glucose/arabinose transport system	0	0	0	0	0	0	0	0	0	0	0	0	0	0	0	0	0	0	0	0	0	0	0	0	0	0	0
M00605	Glucose/mannose transport system	0	0	0	0	0	0	0	0	0	0	0	0	0	0	0	0	0	0	0	0	0	0	0	0	0	0	0
M00604	Trehalose transport system	0	0	0	0	0	0	0	0	0	0	0	0	0	0	0	0	0	0	0	0	0	0	0	0	0	0	0
M00206	Cellobiose transport system	0	0	0	0	0	0	0	0	0	0	0	0	0	0	0	0	0	0	0	0	0	0	0	0	0	0	0
M00606	N,N'-Diacetylchitobiose transport system	0	0	0	0	0	0	0	0	0	0	0	0	0	0	0	0	0	0	0	0	0	0	0	0	0	0	0
M00212	Ribose transport system	0	0	0	0	0	0	0	0	0	0	0	0	0	0	0	0	0	0	0	0	0	0	0	0	0	0	0
M00599	Inositol-phosphate transport system	0	0	0	0	0	0	0	0	0	0	0	0	0	0	0	0	0	0	0	0	0	0	0	0	0	0	0
M00197	Putative fructooligosaccharide transport system	0	0	0	0	0	0	0	0	0	0	0	0	0	0	0	0	0	0	0	0	0	0	0	0	0	0	0
M00198	Putative sn-glycerol-phosphate transport system	0	0	0	0	0	0	0	0	0	0	0	0	0	0	0	0	0	0	0	0	0	0	0	0	0	0	0
M00200	Putative sorbitol/mannitol transport system	0	0	0	0	0	0	0	0	0	0	0	0	0	0	0	0	0	0	0	0	0	0	0	0	0	0	0
M00602	Arabinosaccharide transport system	0	0	0	0	0	0	0	0	0	0	0	0	0	0	0	0	0	0	0	0	0	0	0	0	0	0	0
M00491	arabinogalactan oligomer/maltooligosaccharide transport system	0	0	0	0	0	0	0	0	0	0	0	0	0	0	0	0	0	0	0	0	0	0	0	0	0	0	0
M00207	Putative multiple sugar transport system	0	0	0	0	0	0	0	0	0	0	0	0	0	0	0	0	0	0	0	0	0	0	0	0	0	0	0
M00221	Putative simple sugar transport system	0	0	0	0	0	0	0	0	0	0	0	0	0	0	0	0	0	0	0	0	0	0	0	0	0	0	0
M00211	Putative ABC transport system (Saccharide and polyol_2)	50	50	50	50	100	100	100	0	100	100	100	0	0	0	0	0	0	0	0	0	0	0	0	0	0	0	0
M00222	Phosphate transport system	100	100	100	100	100	100	100	100	75	100	100	100	100	100	100	100	100	100	100	75	100	100	0	100	100	100	75
M00223	Phosphonate transport system	0	0	0	0	0	0	0	0	0	33.3	0	0	0	0	0	0	0	0	0	0	0	0	0	0	0	0	0
M00237	Branched-chain amino acid transport system	100	100	0	0	0	0	20	0	0	20	0	20	100	0	0	20	0	20	0	0	0	0	0	0	0	0	0
M00236	Putative polar amino acid transport system	0	33.3	33.3	0	0	0	0	0	0	66.7	100	33.3	100	66.7	100	100	0	100	100	0	0	100	100	0	0	0	0
M00439	Oligopeptide transport system	0	0	0	0	0	0	0	0	0	0	0	20	0	40	0	0	0	0	0	0	0	0	0	0	0	0	0
M00239	Peptides/nickel transport system	0	100	0	0	100	20	100	0	80	100	0	100	100	100	60	100	100	100	100	0	0	100	100	0	0	0	0
M00240	Iron complex transport system	100	100	100	100	100	100	100	100	100	100	100	100	100	100	100	100	100	100	100	100	0	0	100	100	33.3	0	100
M00242	Zinc transport system	0	0	0	0	100	100	100	100	66.7	100	100	100	100	0	100	100	0	0	0	0	100	0	0	100	100	0	0
M00318	Iron/zinc/copper transport system	0	0	0	0	0	0	0	0	0	0	0	0	0	0	0	0	0	0	0	0	0	0	0	0	0	0	0
M00317	Manganese/iron transport system	0	0	0	0	0	0	0	0	0	0	0	0	0	0	0	0	0	0	0	0	0	0	0	0	0	0	0
M00319	Manganese/zinc/iron transport system	0	0	0	0	0	0	0	0	0	0	0	0	0	0	0	0	0	0	0	0	0	0	0	0	0	0	0

ID	Module name	Module completion ratio (%)																										
		mja	mig	mvn	mok	mba	mbu	mmh	mev	mzh	mpy	mhz	mcj	mhu	mla	mbg	mpi	mbn	mpl	mpd	mer	mth	mst	mru	mel	mfv	mka	max
M00243	Manganese/iron transport system	0	0	0	0	0	0	0	0	0	0	0	0	0	0	0	0	0	0	0	0	0	0	0	0	0	0	0
M00245	Cobalt/nickel transport system	100	100	100	100	100	100	100	100	100	100	100	100	100	75	100	100	100	100	100	0	100	100	100	100	100	75	25
M00246	Nickel transport system	100	100	100	100	100	100	100	100	100	100	100	100	100	100	100	100	100	100	100	0	100	100	100	100	100	100	33.3
M00581	Biotin transport system	0	0	0	0	33.3	33.3	33.3	33.3	33.3	33.3	33.3	0	33.3	33.3	33.3	33.3	33.3	33.3	33.3	0	33.3	0	0	33.3	33.3	0	0
M00582	Energy-coupling factor transport system	0	0	25	0	100	100	100	75	100	100	100	0	100	100	100	100	100	100	100	0	100	75	100	25	25	0	0
M00244	Putative zinc/manganese transport system	0	0	0	0	0	0	0	0	0	0	0	100	100	100	100	100	66.7	0	100	0	0	0	0	0	0	0	0
M00298	Multidrug/hemolysin transport system	0	0	0	0	0	0	0	0	0	0	0	0	0	0	0	0	0	0	0	100	0	0	0	0	0	0	0
M00250	Lipopolysaccharide transport system	0	0	0	0	0	0	0	0	0	0	100	100	100	0	0	100	100	100	100	0	0	100	100	100	0	0	100
M00320	Lipopolysaccharide export system	0	0	0	0	0	0	0	0	0	0	0	0	0	0	0	0	0	0	0	0	0	0	0	0	0	0	0
M00252	Lipooligosaccharide transport system	0	0	0	0	0	100	100	100	50	100	100	0	100	0	100	0	100	100	100	0	0	0	0	0	0	0	0
M00224	Fluoroquinolones transport system	0	0	0	0	0	0	0	0	0	0	0	0	0	0	0	0	0	0	100	0	0	0	0	0	0	0	0
M00255	Lipoprotein-releasing system	0	0	0	0	100	0	100	100	100	100	0	100	0	0	0	0	0	0	0	0	0	0	0	0	0	0	0
M00259	Heme transport system	0	0	0	0	0	66.7	100	100	100	100	100	0	0	0	0	0	0	0	0	0	0	0	0	0	0	0	0
M00258	Uncharacterized ABC transport system_2	100	100	100	100	100	100	100	100	100	100	100	100	100	100	100	100	100	100	100	0	100	0	100	100	0	100	0
M00254	Uncharacterized ABC transport system_3	100	100	100	0	100	100	0	100	100	100	100	100	100	50	100	100	100	100	100	100	100	100	100	100	100	100	100
M00273	PTS system, fructose-specific II component	0	0	0	0	0	0	0	0	0	0	0	0	0	0	0	0	0	0	0	0	0	0	0	0	0	0	0
M00279	PTS system, galactitol-specific II component	0	0	0	0	0	0	0	0	0	0	0	0	0	0	0	0	0	0	0	0	0	0	0	0	0	0	0
M00276	PTS system, mannose-specific II component	0	0	0	0	0	0	0	0	0	0	0	0	0	0	0	0	0	0	0	0	0	0	0	0	0	0	0
M00334	Type VI secretion system	0	0	0	0	0	0	0	0	0	0	0	0	0	0	0	0	0	0	0	0	0	0	0	0	0	0	0
M00335	Sec (secretion) system	44.4	44.4	44.4	44.4	44.4	44.4	44.4	44.4	44.4	44.4	44.4	44.4	44.4	44.4	44.4	44.4	44.4	44.4	44.4	25	44.4	44.4	44.4	44.4	44.4	44.4	25

ID	Module name	Module completion ratio (%)																										
		mja	mig	mvn	mok	mba	mbu	mmh	mev	mzh	mpy	mhz	mcj	mhu	mla	mbg	mpi	mbn	mpl	mpd	mer	mth	mst	mru	mel	mfv	mka	max
Functional set																												
Metabolism																												
M00360	Aminoacyl-tRNA biosynthesis, prokaryotes	94.4	94.4	100	100	100	94.4	100	100	100	100	100	94.4	100	94.4	88.9	94.4	100	100	100	100	94.4	100	100	100	94.4	94.4	100
M00359	Aminoacyl-tRNA biosynthesis, eukaryotes	80	80	85	85	90	80	85	85	85	85	85	80	90	80	80	80	90	90	90	85	80	85	85	85	80	80	85
M00362	Nucleotide sugar biosynthesis, prokaryotes	71.4	85.7	71.4	85.7	100	100	85.7	57.1	71.4	85.7	100	71.4	85.7	57.1	57.1	42.9	71.4	57.1	85.7	42.9	71.4	71.4	71.4	71.4	71.4	42.9	28.6
M00361	Nucleotide sugar biosynthesis, eukaryotes	50	50	50	50	66.7	66.7	66.7	50	50	50	66.7	33.3	66.7	50	33.3	50	66.7	33.3	50	33.3	50	50	50	50	50	33.3	33.3
Environmental information processing																												
M00454	KdpD-KdpE (potassium transport) two-component regulatory system	0	0	0	0	0	0	0	0	0	0	0	0	0	0	50	0	50	50	0	0	0	0	0	50	0	0	0
M00459	VicK-VicR (cell wall metabolism) two-component regulatory system	0	0	0	0	0	0	0	0	0	50	0	0	0	0	0	0	0	0	0	0	0	0	0	0	0	0	0
M00460	MprB-MprA (maintenance of persistent infection two-component regulatory system	0	0	0	0	0	0	0	0	0	0	0	0	0	0	0	0	0	0	0	0	0	0	0	0	0	0	0
M00506	CheA-CheYBV (chemotaxis) two-component regulatory system	0	0	60	60	80	80	0	60	60	80	60	0	60	0	60	60	80	80	40	0	0	0	0	20	0	0	0
M00507	ChpA-ChpB/PilGH (chemosensory) two-component regulatory system	0	0	0	0	0	0	0	0	0	0	0	0	0	0	0	20	0	0	0	0	0	0	0	0	0	0	0
M00511	PleC-PleD (cell fate control) two-component regulatory system	0	0	0	0	0	0	16.7	16.7	0	16.7	16.7	0	0	0	0	0	0	0	0	0	0	0	0	0	0	0	0
M00520	ChvG-ChvI (acidity sensing) two-component regulatory system	0	0	0	0	0	0	0	0	0	0	0	0	0	0	0	0	0	0	0	0	0	0	0	0	0	0	0

Full species name of each abbreviation is described in Table S2.

Table S3 Module completion patterns in 79 archaeal species listed in Table S2 (continied: Halophilic and other euryarchaeota).

ID	Module name	Module completion ratio (%)																									
		afu	fpl	hsl	hma	hwa	nph	hla	hut	hmu	htu	nmg	hvo	hje	hbo	hxa	npe	nge	hru	nou	sali	tac	pto	fac	pfu	tko	abi
Pathway module																											
Energy metabolism																											
M00165	Reductive pentose phosphate cycle (Calvin cycle)	36.4	45.5	36.4	36.4	36.4	45.5	36.4	63.6	45.5	36.4	45.5	63.6	36.4	63.6	36.4	36.4	36.4	36.4	36.4	36.4	45.5	45.5	45.5	54.5	54.5	63.6
M00166	Reductive pentose phosphate cycle, ribulose-5P => glyceraldehyde-3P	75	100	50	50	50	75	50	50	75	50	75	75	50	75	50	50	50	50	50	50	50	50	50	75	75	100
M00167	Reductive pentose phosphate cycle, glyceraldehyde-3P => ribulose-5P	14.3	14.3	28.6	28.6	28.6	28.6	28.6	71.4	28.6	28.6	28.6	57.1	28.6	57.1	28.6	28.6	28.6	28.6	28.6	28.6	42.9	42.9	42.9	42.9	42.9	42.9
M00168	CAM (Crassulacean acid metabolism), dark	100	100	100	100	100	50	100	50	100	100	100	100	100	100	100	100	100	50	100	100	50	100	100	50	0	0
M00169	CAM (Crassulacean acid metabolism), light	0	0	50	50	50	50	50	50	50	50	50	50	50	50	50	50	50	50	50	50	50	0	0	0	0	50
M00172	C4-dicarboxylic acid cycle, NADP - malic enzyme type	25	25	50	50	50	25	50	50	50	50	50	50	50	50	50	50	50	25	50	50	25	25	25	25	0	25
M00171	C4-dicarboxylic acid cycle, NAD - malic enzyme type	14.3	14.3	14.3	14.3	14.3	0	14.3	14.3	14.3	14.3	14.3	14.3	14.3	14.3	14.3	14.3	14.3	0	14.3	14.3	14.3	14.3	14.3	14.3	0	14.3
M00170	C4-dicarboxylic acid cycle, phosphoenolpyruvate carboxykinase type	25	25	25	25	25	0	50	25	25	50	50	25	50	25	50	50	50	25	50	25	0	25	25	25	0	0
M00173	Reductive citrate cycle (Arnon-Buchanan cycle)	81.8	81.8	81.8	81.8	81.8	72.7	81.8	72.7	81.8	81.8	81.8	81.8	81.8	81.8	81.8	81.8	81.8	72.7	81.8	81.8	72.7	81.8	72.7	54.5	27.3	45.5
M00376	3-Hydroxypropionate bi-cycle	18.8	25	25	25	25	25	25	12.5	25	25	25	25	25	25	25	25	25	25	25	25	22.2	22.2	11.1	18.8	18.8	18.8
M00375	Hydroxypropionate-hydroxybutylate cycle	33.3	33.3	33.3	33.3	16.7	25	33.3	8.3	33.3	33.3	33.3	33.3	33.3	33.3	33.3	33.3	33.3	33.3	33.3	33.3	28.6	28.6	21.4	25	25	25
M00374	Dicarboxylate-hydroxybutyrate cycle	76.9	69.2	61.5	61.5	46.2	46.2	61.5	46.2	61.5	61.5	61.5	61.5	61.5	61.5	61.5	61.5	61.5	53.8	61.5	61.5	46.2	61.5	53.8	30.8	23.1	30.8
M00377	Reductive acetyl-CoA pathway (Wood-Ljungdahl pathway)	14.3	14.3	14.3	42.9	42.9	14.3	28.6	14.3	14.3	14.3	14.3	14.3	14.3	28.6	14.3	14.3	42.9	14.3	42.9	14.3	28.6	28.6	28.6	0	0	28.6
M00579	Phosphate acetyltransferase-acetate kinase pathway, acetyl-CoA => acetate	0	0	0	0	0	0	0	0	0	0	0	0	0	0	0	0	0	0	0	0	0	0	0	0	0	0
M00175	Nitrogen fixation, nitrogen => ammonia	0	0	0	0	0	0	0	0	0	0	0	0	0	0	0	0	0	0	0	0	0	0	0	0	0	0
M00531	Assimilatory nitrate reduction, nitrate => ammonia	50	50	0	100	100	100	100	50	50	100	50	100	100	50	100	100	0	0	100	50	0	0	0	0	0	0
M00530	Dissimilatory nitrate reduction, nitrate => ammonia	0	50	0	0	0	0	0	0	0	0	0	0	0	0	0	0	0	0	0	0	0	0	0	0	0	0
M00529	Denitrification, nitrate => nitrogen	0	75	0	50	0	25	25	25	25	25	0	25	0	50	50	25	0	25	25	25	0	0	0	0	0	0
M00528	Nitrification, ammonia => nitrite	0	0	0	0	0	0	0	0	0	0	0	0	0	0	0	0	0	0	0	0	0	0	0	0	0	0
M00567	Methanogenesis, CO2 => methane	75	75	12.5	25	25	25	25	25	25	25	25	25	25	25	25	25	25	25	25	25	0	0	0	0	0	12.5
M00357	Methanogenesis, acetate => methane	60	60	20	20	20	20	20	20	20	20	20	20	20	20	20	20	20	20	20	20	20	20	20	0	0	20
M00356	Methanogenesis, methanol => methane	33.3	33.3	0	0	0	0	0	0	0	0	0	0	0	0	0	0	0	0	0	0	0	0	0	0	0	33.3
M00563	Methanogenesis, mono-/di-/tri-methylamine => methane	25	25	0	0	0	0	0	0	0	0	0	0	0	0	0	0	0	0	0	0	0	0	0	0	0	25
M00358	Coenzyme M biosynthesis	0	0	0	0	0	0	0	0	0	0	0	25	0	0	0	0	0	0	0	0	25	25	25	0	0	0
M00174	Methane oxidation, methanotroph, methane => formaldehyde	0	0	0	0	0	0	0	0	0	0	0	0	0	0	0	0	0	0	0	0	0	0	0	0	0	0
M00346	Formaldehyde assimilation, serine pathway	44.4	44.4	44.4	55.6	44.4	44.4	55.6	33.3	44.4	55.6	55.6	55.6	55.6	44.4	55.6	55.6	55.6	44.4	55.6	55.6	55.6	66.7	55.6	44.4	22.2	22.2

ID	Module name	Module completion ratio (%)																									
		afu	fpl	hsl	hma	hwa	nph	hla	hut	hmu	htu	nmg	hvo	hje	hbo	hxa	npe	nge	hru	nou	sali	tac	pto	fac	pfu	tko	abi
M00345	Formaldehyde assimilation, ribulose monophosphate pathway	50	50	0	0	0	0	0	33.3	0	0	0	33.3	0	33.3	0	0	0	0	0	0	0	33.3	0	33.3	33.3	33.3
M00344	Formaldehyde assimilation, xylulose monophosphate pathway	0	0	25	25	25	25	25	25	25	25	25	50	25	50	25	25	25	25	25	25	0	0	0	0	0	0
M00378	F420 biosynthesis	80	80	60	60	40	60	60	60	60	60	60	60	60	60	60	60	60	60	60	60	0	0	0	0	0	0
M00422	Acetyl-CoA pathway, CO2 => acetyl-CoA	100	100	0	0	0	0	0	0	0	0	0	0	0	0	0	0	0	0	0	0	0	0	0	0	0	0
M00176	Assimilatory sulfate reduction, sulfate => H2S	33.3	33.3	33.3	33.3	33.3	33.3	33.3	33.3	33.3	33.3	33.3	33.3	33.3	33.3	33.3	33.3	33.3	33.3	33.3	33.3	0	0	33.3	33.3	33.3	66.7
M00596	Dissimilatory sulfate reduction, sulfate => H2S	100	66.7	0	0	0	0	0	0	0	0	0	0	0	0	0	0	0	0	0	0	0	0	33.3	33.3	0	33.3
Carbohydrate & lipid metabolism																											
M00001	Glycolysis (Embden-Meyerhof pathway), glucose => pyruvate	70	90	90	90	90	80	90	90	90	90	100	90	90	90	90	90	100	100	90	90	80	90	80	100	100	80
M00002	Glycolysis, core module involving three-carbon compounds	83.3	100	100	100	100	100	100	100	100	100	100	100	100	100	100	100	100	100	100	100	100	100	100	100	100	100
M00003	Gluconeogenesis, oxaloacetate => fructose-6P	62.5	62.5	50	62.5	62.5	50	75	62.5	62.5	100	100	87.5	100	75	100	100	100	87.5	75	62.5	100	62.5	100	100	100	100
M00307	Pyruvate oxidation, pyruvate => acetyl-CoA	100	100	100	100	0	100	100	100	100	100	100	100	100	100	100	100	100	100	100	100	100	100	0	0	0	100
M00009	Citrate cycle (TCA cycle, Krebs cycle)	87.5	75	87.5	87.5	87.5	87.5	87.5	87.5	87.5	87.5	87.5	87.5	87.5	87.5	87.5	87.5	87.5	87.5	87.5	87.5	87.5	75	62.5	62.5	25	62.5
M00010	Citrate cycle, first carbon oxidation, oxaloacetate => 2-oxoglutarate	66.7	66.7	100	100	100	100	100	100	100	100	100	100	100	100	100	100	100	100	100	100	100	100	100	100	0	66.7
M00011	Citrate cycle, second carbon oxidation, 2-oxoglutarate => oxaloacetate	100	80	80	80	80	80	80	80	80	80	80	80	80	80	80	80	80	80	80	80	80	60	40	40	40	60
M00004	Pentose phosphate pathway (Pentose phosphate cycle)	28.6	28.6	42.9	42.9	42.9	42.9	42.9	71.4	42.9	42.9	42.9	42.9	42.9	42.9	42.9	42.9	42.9	42.9	42.9	50	71.4	71.4	71.4	42.9	42.9	71.4
M00006	Pentose phosphate pathway, oxidative phase, glucose 6P => ribulose 5P	0	0	50	50	50	50	50	0	50	50	50	50	50	50	50	50	50	50	50	66.7	0	0	0	0	0	0
M00007	Pentose phosphate pathway, non-oxidative phase,fructose 6P => ribose 5P	25	25	25	25	25	25	25	100	25	25	25	25	25	25	25	25	25	25	25	25	100	100	100	50	50	100
M00580	Pentose phosphate pathway, archaea, fructose 6P => ribose 5P	100	100	50	50	50	50	50	50	50	50	50	50	50	50	50	50	50	50	50	50	50	50	50	100	100	100
M00005	PRPP biosynthesis, ribose 5P => PRPP	100	100	100	100	100	100	100	100	100	100	100	100	100	100	100	100	100	100	100	100	100	100	100	100	100	100
M00008	Entner-Doudoroff pathway, glucose-6P => glyceraldehyde-3P + pyruvate	0	0	0	25	25	0	25	25	25	25	25	25	0	25	25	0	25	25	25	50	0	0	0	0	0	0
M00308	Semi-phosphorylative Entner-Doudoroff pathway, gluconate => glyceraldehyde-3P + pyruvate	20	20	75	100	100	20	100	80	100	100	100	100	80	100	100	75	100	100	100	100	40	40	40	25	25	20
M00633	Semi-phosphorylative Entner-Doudoroff pathway, gluconate/ galactonate => glycerate-3P	0	0	75	25	25	0	25	0	25	50	50	25	25	25	25	50	25	25	25	25	50	50	50	25	25	0
M00309	Non-phosphorylative Entner-Doudoroff pathway, gluconate => glyceraldehyde + pyruvate	33.3	33.3	66.7	66.7	33.3	33.3	66.7	0	33.3	66.7	66.7	66.7	66.7	66.7	66.7	66.7	66.7	33.3	66.7	33.3	100	100	100	33.3	33.3	33.3
M00012	Glyoxylate cycle	40	40	60	60	80	60	60	40	60	60	60	60	60	60	60	60	60	60	60	60	60	60	60	40	0	20
M00373	Ethylmalonyl pathway	7.1	21.4	21.4	21.4	14.3	14.3	21.4	7.1	21.4	21.4	21.4	21.4	21.4	21.4	21.4	21.4	21.4	28.6	21.4	21.4	7.1	7.1	7.1	21.4	21.4	21.4
M00532	Photorespiration	20	30	30	40	20	50	40	20	40	50	60	50	40	40	50	40	40	50	50	40	40	40	20	30	30	40
M00013	Malonate semialdehyde pathway, propanoyl-CoA => Acetyl-CoA	25	25	0	25	0	25	25	0	25	50	50	25	25	25	0	50	25	0	50	25	25	25	0	0	0	0
M00632	Galactose degradation, Leloir pathway, galactose => alpha-D-glucose-1P	25	25	25	25	25	25	25	50	25	25	25	50	25	25	25	25	25	25	25	25	25	50	25	75	75	50

ID	Module name	Module completion ratio (%)																									
		afu	fpl	hsl	hma	hwa	nph	hla	hut	hmu	htu	nmg	hvo	hje	hbo	hxa	npe	nge	hru	nou	sali	tac	pto	fac	pfu	tko	abi
M00552	D-galactonate degradation, De Ley-Doudoroff pathway, D-galactonate => glycerate-3P	20	20	20	60	40	20	60	40	60	40	40	60	60	60	60	20	40	40	60	60	40	20	40	20	20	20
M00014	Glucuronate pathway (uronate pathway)	28.6	14.3	14.3	14.3	14.3	14.3	0	28.6	14.3	14.3	14.3	28.6	28.6	14.3	14.3	14.3	14.3	14.3	28.6	14.3	0	0	14.3	14.3	0	0
M00631	D-Galacturonate degradation (bacteria), D-galacturonate => pyruvate + D-glyceraldehyde 3P	0	0	20	40	40	0	40	60	40	80	40	60	40	40	60	20	40	40	40	40	0	20	0	0	0	0
M00061	Uronic acid metabolism	0	0	20	40	40	0	60	80	40	80	40	40	40	40	80	20	40	40	40	60	20	20	20	0	0	0
M00081	Pectin degradation	0	0	0	33.3	0	0	0	0	0	33.3	0	0	0	0	33.3	0	0	0	0	0	0	0	0	0	0	0
M00082	Fatty acid biosynthesis, initiation	0	0	0	0	0	0	0	0	33.3	33.3	0	0	0	66.7	0	0	0	33.3	0	33.3	0	0	0	0	0	0
M00113	Jasmonic acid biosynthesis	12.5	0	12.5	12.5	12.5	12.5	12.5	0	12.5	12.5	12.5	12.5	12.5	12.5	12.5	12.5	12.5	12.5	12.5	0	0	0	0	0	0	0
M00086	beta-Oxidation, acyl-CoA synthesis	100	100	100	100	0	100	100	0	100	100	100	100	100	100	100	100	100	100	100	100	0	0	100	0	0	0
M00087	beta-Oxidation	66.7	33.3	33.3	66.7	33.3	66.7	66.7	0	33.3	66.7	66.7	33.3	66.7	66.7	33.3	66.7	66.7	33.3	66.7	33.3	33.3	33.3	25	0	0	0
M00088	Ketone body biosynthesis, acetyl-CoA => acetoacetate/3-hydroxybutyrate/acetone	40	40	40	60	20	40	60	60	40	60	60	60	60	60	40	80	40	40	60	60	40	40	40	40	40	40
M00093	Phosphatidylethanolamine (PE) biosynthesis, PA => PS => PE	66.7	66.7	66.7	33.3	66.7	66.7	66.7	33.3	33.3	66.7	66.7	66.7	66.7	66.7	66.7	66.7	66.7	66.7	66.7	66.7	0	0	0	0	0	0
M00131	Inositol phosphate metabolism, Ins(1,3,4,5)P4 => Ins(1,3,4)P3 => myo-inositol	25	25	25	25	25	25	25	25	25	25	25	25	25	25	25	25	25	25	25	25	0	0	0	25	25	25
M00094	Ceramide biosynthesis	0	0	0	0	0	0	0	0	0	0	0	0	0	0	0	0	0	0	0	0	25	0	0	0	0	0
M00099	Sphingosine biosynthesis	0	0	0	0	0	0	0	0	0	0	0	0	0	0	0	0	0	0	0	0	20	0	0	0	0	0
M00060	Lipopolysaccharide biosynthesis, KDO2-lipid A	0	0	0	0	0	0	0	0	0	0	0	0	11.1	0	0	0	0	0	0	0	0	0	0	0	0	0
M00064	ADP-L-glycero-D-manno-heptose biosynthesis	0	0	0	0	0	0	0	0	0	0	0	0	0	0	0	0	0	0	0	0	25	25	50	0	0	0
M00080	Lipopolysaccharide biosynthesis, inner core => outer core => O-antigen	0	0	0	0	0	0	0	0	0	0	0	0	0	0	0	0	0	0	0	0	0	0	0	0	0	0
M00549	Nucleotide sugar biosynthesis, glucose => UDP-glucose	0	66.7	33.3	33.3	33.3	0	33.3	33.3	33.3	33.3	33.3	33.3	33.3	33.3	33.3	33.3	33.3	33.3	33.3	33.3	33.3	33.3	33.3	33.3	33.3	33.3
M00554	Nucleotide sugar biosynthesis, galactose => UDP-galactose	0	0	0	0	0	0	0	50	0	0	0	50	0	0	0	0	0	0	0	0	0	0	0	100	100	50
M00565	Trehalose biosynthesis, D-glucose 1P => trehalose	0	0	0	0	0	0	0	0	0	0	0	0	0	0	0	0	0	0	0	0	0	20	0	40	40	0
M00055	N-glycan precursor biosynthesis	0	7.7	0	0	0	0	0	0	0	0	0	0	0	0	0	0	0	0	0	0	0	0	0	7.7	7.7	7.7
M00076	Dermatan sulfate degradation	0	0	0	0	0	0	0	20	0	20	0	0	0	0	0	0	0	0	0	0	0	0	0	0	0	0
M00077	Chondroitin sulfate degradation	0	0	0	0	0	0	0	25	0	25	0	0	0	0	0	0	0	0	0	0	0	0	0	0	0	0
M00078	Heparan sulfate degradation	0	0	0	0	0	0	0	12.5	0	12.5	0	0	0	0	0	0	0	0	0	0	0	0	0	0	0	0
M00095	C5 isoprenoid biosynthesis, mevalonate pathway	57.1	57.1	71.4	71.4	57.1	57.1	71.4	71.4	71.4	71.4	71.4	71.4	71.4	71.4	71.4	71.4	71.4	71.4	71.4	71.4	42.9	57.1	57.1	71.4	71.4	57.1
M00096	C5 isoprenoid biosynthesis, non-mevalonate pathway	12.5	12.5	12.5	25	12.5	12.5	12.5	12.5	12.5	12.5	12.5	12.5	12.5	12.5	12.5	12.5	12.5	12.5	12.5	12.5	12.5	12.5	12.5	12.5	12.5	25
M00364	C10-C20 isoprenoid biosynthesis, bacteria	100	100	100	100	100	100	100	100	100	100	100	100	100	100	100	100	100	100	100	100	100	100	100	100	100	100
M00365	C10-C20 isoprenoid biosynthesis, archaea	100	100	100	100	100	100	100	100	100	100	100	100	100	100	100	100	100	100	100	100	100	100	100	100	100	100

ID	Module name	Module completion ratio (%)																									
		afu	fpl	hsl	hma	hwa	nph	hla	hut	hmu	htu	nmg	hvo	hje	hbo	hxa	npe	nge	hru	nou	sali	tac	pto	fac	pfu	tko	abi
M00366	C10-C20 isoprenoid biosynthesis, plants	25	25	25	25	25	25	25	25	25	25	25	25	25	25	25	25	25	25	25	25	25	25	25	25	25	25
M00367	C10-C20 isoprenoid biosynthesis, non-plant eukaryotes	33.3	33.3	33.3	33.3	33.3	33.3	33.3	33.3	33.3	33.3	33.3	33.3	33.3	33.3	33.3	33.3	33.3	33.3	33.3	33.3	33.3	33.3	33.3	33.3	33.3	33.3
M00097	beta-Carotene biosynthesis, GGAP => beta-carotene	0	0	16.7	16.7	16.7	16.7	16.7	16.7	16.7	16.7	16.7	16.7	16.7	16.7	16.7	16.7	16.7	16.7	16.7	16.7	0	16.7	0	0	0	0
M00372	Abscisic acid biosynthesis, beta-carotene => abscisic acid	0	0	0	0	0	0	0	0	0	0	0	0	0	0	0	0	0	0	0	0	0	16.7	0	0	0	0
Nucleotide & amino acid metabolism																											
M00048	Inosine monophosphate biosynthesis, PRPP + glutamine => IMP	80	72.7	90	90	100	90	90	90	90	90	90	90	90	90	90	90	90	90	90	90	100	100	100	90.9	100	90
M00049	Adenine ribonucleotide biosynthesis, IMP => ADP, ATP	100	100	100	100	100	100	100	100	100	100	100	100	100	100	100	100	100	100	100	100	100	100	100	100	100	100
M00050	Guanine ribonucleotide biosynthesis IMP => GDP, GTP	75	75	75	75	75	75	75	75	75	75	75	75	75	75	75	75	75	75	75	75	75	75	75	75	75	75
M00546	Purine degradation, xanthine => urea	0	0	0	0	0	0	0	0	0	0	0	50	50	0	20	0	20	0	66.7	0	0	0	0	0	0	0
M00051	Uridine monophosphate biosynthesis, glutamine (+ PRPP) => UMP	100	100	100	100	100	100	100	100	100	100	100	100	100	100	83.3	83.3	83.3	83.3	83.3	100	100	100	100	100	83.3	83.3
M00052	Pyrimidine ribonucleotide biosynthesis, UMP => UDP/UTP, CDP/CTP	100	100	100	100	100	100	100	100	100	100	100	100	100	100	100	100	100	100	100	100	100	100	100	100	100	100
M00053	Pyrimidine deoxyribonuleotide biosynthesis, CDP/CTP => dCDP/dCTP, dTDP/dTTP	62.5	62.5	75	62.5	62.5	75	87.5	87.5	87.5	87.5	87.5	75	75	87.5	75	87.5	87.5	87.5	62.5	87.5	50	50	50	62.5	62.5	62.5
M00046	Pyrimidine degradation, uracil => beta-alanine, thymine => 3-aminoisobutanoate	0	0	0	0	0	0	33.3	0	0	33.3	33.3	33.3	33.3	0	0	0	0	0	33.3	0	0	0	33.3	0	0	0
M00020	Serine biosynthesis, glycerate-3P => serine	66.7	66.7	66.7	66.7	66.7	66.7	66.7	66.7	66.7	66.7	66.7	66.7	66.7	66.7	66.7	66.7	66.7	66.7	66.7	66.7	33.3	66.7	66.7	66.7	66.7	33.3
M00018	Threonine biosynthesis, aspartate => homoserine => threonine	80	80	100	100	100	100	100	100	100	100	100	100	100	100	100	100	100	100	100	100	100	100	100	100	100	20
M00555	Betaine biosynthesis, choline => betaine	0	0	0	0	0	0	0	0	0	0	0	0	0	0	0	0	0	0	50	0	0	0	0	0	0	0
M00021	Cysteine biosynthesis, serine => cysteine	0	0	100	100	100	100	50	100	100	100	100	100	100	100	100	100	100	50	100	50	50	50	50	50	50	50
M00338	Cysteine biosynthesis, homocysteine + serine => cysteine	0	0	50	0	0	0	0	0	0	50	50	50	50	0	100	0	50	50	0	0	50	0	0	0	0	0
M00609	Cysteine biosynthesis, methionine => cysteine	16.7	16.7	16.7	16.7	16.7	16.7	16.7	16.7	16.7	16.7	16.7	16.7	16.7	16.7	16.7	16.7	16.7	16.7	16.7	16.7	16.7	16.7	16.7	16.7	16.7	16.7
M00017	Methionine biosynthesis, apartate => homoserine => methionine	57.1	57.1	57.1	71.4	57.1	57.1	57.1	71.4	57.1	57.1	57.1	57.1	57.1	57.1	57.1	57.1	57.1	57.1	57.1	57.1	71.4	71.4	71.4	71.4	71.4	0
M00034	Methionine salvage pathway	62.5	62.5	12.5	25	37.5	12.5	25	12.5	25	12.5	25	37.5	25	25	25	12.5	12.5	12.5	25	12.5	50	50	62.5	50	50	62.5
M00035	Methionine degradation	50	75	50	50	50	50	50	50	75	75	75	75	75	50	100	75	75	75	50	50	50	50	75	75	50	50
M00368	Ethylene biosynthesis, methionine => ethylene	33.3	33.3	33.3	33.3	33.3	33.3	33.3	33.3	33.3	33.3	33.3	33.3	33.3	33.3	33.3	33.3	33.3	33.3	33.3	33.3	33.3	33.3	33.3	33.3	33.3	33.3
M00019	Valine/isoleucine biosynthesis, pyruvate => valine / 2-oxobutanoate => isoleucine	100	100	25	100	100	100	100	100	100	100	100	100	100	100	100	100	100	100	100	100	0	50	50	50	0	0
M00535	Isoleucine biosynthesis, pyruvate => 2-oxobutanoate	100	100	0	100	100	100	100	100	100	100	100	100	100	100	100	100	100	66.7	100	100	0	66.7	66.7	66.7	33.3	66.7
M00570	Isoleucine biosynthesis, threonine => 2-oxobutanoate => isoleucine	80	80	40	100	100	100	100	100	100	100	100	100	100	100	100	100	100	100	100	100	20	60	60	40	0	0
M00432	Leucine biosynthesis, 2-oxoisovalerate => 2-oxoisocaproate	100	100	0	100	66.7	100	100	100	100	100	100	100	100	100	100	100	100	66.7	100	100	0	100	100	100	66.7	66.7
M00036	Leucine degradation, leucine => acetoacetate + acetyl-CoA	33.3	33.3	16.7	33.3	16.7	33.3	50	16.7	16.7	33.3	33.3	33.3	33.3	33.3	16.7	33.3	33.3	16.7	33.3	33.3	16.7	16.7	16.7	0	0	0

ID	Module name	Module completion ratio (%)																									
		afu	fpl	hsl	hma	hwa	nph	hla	hut	hmu	htu	nmg	hvo	hje	hbo	hxa	npe	nge	hru	nou	sali	tac	pto	fac	pfu	tko	abi
M00016	Lysine biosynthesis, succinyl-DAP pathway, aspartate => lysine	88.9	88.9	22.2	77.8	88.9	66.7	88.9	66.7	66.7	77.8	77.8	88.9	77.8	77.8	77.8	77.8	77.8	33.3	66.7	66.7	44.4	44.4	55.6	44.4	44.4	11.1
M00525	Lysine biosynthesis, acetyl-DAP pathway, aspartate => lysine	66.7	66.7	22.2	55.6	66.7	55.6	66.7	55.6	55.6	66.7	55.6	66.7	66.7	66.7	55.6	66.7	55.6	33.3	55.6	55.6	33.3	33.3	44.4	33.3	33.3	0
M00526	Lysine biosynthesis, DAP dehydrogenase pathway, aspartate => lysine	83.3	83.3	33.3	83.3	83.3	83.3	83.3	83.3	83.3	83.3	83.3	83.3	83.3	83.3	83.3	83.3	83.3	50	83.3	83.3	50	50	66.7	50	50	0
M00527	Lysine biosynthesis, DAP aminotransferase pathway, aspartate => lysine	100	100	28.6	71.4	85.7	71.4	85.7	71.4	71.4	85.7	71.4	85.7	85.7	85.7	71.4	85.7	71.4	42.9	71.4	71.4	42.9	42.9	57.1	42.9	42.9	0
M00030	Lysine biosynthesis, AAA pathway, 2-oxoglutarate => 2-aminoadipate => lysine	0	0	0	0	0	0	0	0	0	0	0	0	0	0	0	0	0	0	0	0	0	0	0	0	0	0
M00433	Lysine biosynthesis, 2-oxoglutarate => 2-oxoadipate	0	0	0	0	0	0	0	0	0	0	0	0	0	0	0	0	0	0	0	0	0	0	0	0	0	0
M00031	Lysine biosynthesis, 2-aminoadipate => lysine	0	0	0	100	100	100	100	100	100	100	100	100	100	100	100	100	100	100	100	100	0	100	100	100	100	0
M00032	Lysine degradation, lysine => saccharopine => acetoacetyl-CoA	14.3	0	14.3	14.3	0	14.3	14.3	0	14.3	14.3	14.3	14.3	14.3	14.3	14.3	14.3	14.3	14.3	14.3	14.3	0	0	12.5	0	0	0
M00608	2-oxocarboxylic acid chain extension, 2-oxoglutarate => 2-oxoadipate => 2-oxopimelate => 2-oxosuberate	0	0	0	0	0	0	0	0	0	0	0	0	0	0	0	0	0	0	0	0	0	0	0	0	0	0
M00015	Proline biosynthesis, glutamate => proline	0	50	0	0	100	100	0	100	100	100	0	100	0	0	0	100	100	0	0	100	50	50	50	50	50	0
M00028	Ornithine biosynthesis, glutamate => ornithine	100	100	0	80	80	80	80	80	80	100	80	80	80	80	100	80	80	80	80	100	0	60	60	80	80	0
M00029	Urea cycle	60	60	60	80	60	60	80	60	80	80	80	80	80	80	80	80	60	80	80	80	60	80	60	60	20	20
M00026	Histidine biosynthesis, PRPP => histidine	77.8	88.9	88.9	88.9	88.9	88.9	100	100	100	100	100	88.9	88.9	88.9	100	100	100	88.9	100	88.9	14.3	87.5	87.5	87.5	87.5	0
M00045	Histidine degradation, histidine => N-formiminoglutamate => glutamate	0	0	100	100	0	0	0	0	0	100	75	100	0	75	0	0	75	75	75	0	75	100	50	0	0	75
M00022	Shikimate pathway, phosphoenolpyruvate + erythrose-4P => chorismate	71.4	71.4	71.4	71.4	71.4	71.4	71.4	71.4	71.4	71.4	71.4	71.4	71.4	71.4	71.4	71.4	71.4	71.4	71.4	71.4	100	100	100	100	100	0
M00023	Tryptophan biosynthesis, chorismate => tryptophan	75	100	100	100	100	100	100	100	100	100	100	100	100	100	100	100	100	100	100	100	100	100	100	100	100	0
M00024	Phenylalanine biosynthesis, chorismate => phenylalanine	50	50	66.7	66.7	66.7	66.7	66.7	66.7	66.7	66.7	66.7	66.7	66.7	66.7	66.7	66.7	66.7	66.7	66.7	33.3	33.3	33.3	66.7	66.7	33.3	0
M00025	Tyrosine biosynthesis, chorismate => tyrosine	33.3	33.3	66.7	66.7	66.7	66.7	66.7	66.7	66.7	66.7	66.7	66.7	66.7	66.7	66.7	66.7	66.7	66.7	66.7	33.3	33.3	33.3	50	66.7	66.7	0
M00042	Catecholamine biosynthesis, tyrosine => dopamine => noradrenaline => adrenaline	25	25	25	25	0	25	25	25	25	25	25	25	25	25	25	25	25	25	25	25	0	0	0	25	25	25
M00044	Tyrosine degradation, tyrosine => homogentisate	0	0	0	0	0	0	0	0	0	0	0	0	0	0	0	0	0	0	0	0	0	40	40	0	0	0
M00038	Tryptophan metabolism, tryptophan => kynurenine => 2-aminomuconate	0	0	14.3	0	0	0	0	0	0	0	0	14.3	0	14.3	0	0	0	28.6	0	0	0	0	0	0	0	0
M00027	GABA (gamma-Aminobutyrate) shunt	0	0	66.7	66.7	33.3	33.3	66.7	0	66.7	66.7	66.7	66.7	66.7	66.7	66.7	66.7	66.7	66.7	66.7	66.7	33.3	33.3	100	33.3	33.3	33.3
M00127	Thiamine biosynthesis, AIR => thiamine-P/thiamine-2P	100	80	60	80	80	100	40	80	80	100	100	100	40	100	100	100	100	100	80	100	60	60	60	100	60	80
M00125	Riboflavin biosynthesis, GTP => riboflavin/FMN/FAD	66.7	66.7	50	50	50	50	50	66.7	50	50	50	50	50	50	50	50	50	50	50	50	16.7	66.7	66.7	83.3	83.3	50
M00124	Pyridoxal biosynthesis, erythrose-4P => pyridoxal-5P	0	0	0	0	0	0	0	0	0	0	0	16.7	16.7	0	0	0	0	0	16.7	16.7	0	0	16.7	0	0	0
M00115	NAD biosynthesis, aspartate => NAD	60	60	80	80	60	80	20	80	80	80	80	80	20	80	80	80	80	80	80	80	20	20	20	80	80	60
M00622	Nicotinate degradation, nicotinate => fumarate	0	0	0	0	0	0	0	0	0	16.7	0	0	16.7	16.7	0	16.7	16.7	0	16.7	0	0	0	16.7	16.7	16.7	0
M00119	Pantothenate biosynthesis, valine/L-aspartate => pantothenate	40	40	60	60	40	60	60	60	60	60	60	60	60	60	60	60	60	60	60	60	0	0	0	40	40	20

ID	Module name	Module completion ratio (%)																									
		afu	fpl	hsl	hma	hwa	nph	hla	hut	hmu	htu	nmg	hvo	hje	hbo	hxa	npe	nge	hru	nou	sali	tac	pto	fac	pfu	tko	abi
M00120	Coenzyme A biosynthesis, pantothenate => CoA	50	50	50	50	50	50	50	50	50	50	50	50	50	50	50	50	50	50	50	50	50	75	75	75	75	33.3
M00572	Pimeloyl-ACP biosynthesis, BioC-BioH pathway, malonyl-ACP => pimeloyl-ACP	0	0	16.7	16.7	16.7	16.7	16.7	16.7	16.7	33.3	16.7	16.7	16.7	33.3	16.7	16.7	16.7	16.7	16.7	16.7	16.7	16.7	16.7	16.7	16.7	16.7
M00123	Biotin biosynthesis, pimeloyl-ACP/CoA => biotin	0	0	0	50	0	75	0	0	75	75	0	25	0	75	50	75	75	25	75	75	0	0	0	25	25	25
M00573	Biotin biosynthesis, BioI pathway, long-chain-acyl-ACP => pimeloyl-ACP => biotin	0	0	0	40	0	60	0	0	60	60	0	20	0	60	40	60	60	20	60	60	0	0	0	20	20	20
M00577	Biotin biosynthesis, BioW pathway, pimelate => pimeloyl-CoA => biotin	0	0	0	40	0	60	0	0	60	60	0	20	0	60	40	60	60	20	60	60	0	0	0	20	20	20
M00126	Tetrahydrofolate biosynthesis, GTP => THF	0	0	33.3	66.7	33.3	33.3	33.3	16.7	50	50	33.3	33.3	16.7	50	50	50	33.3	33.3	50	50	33.3	33.3	50	33.3	16.7	33.3
M00121	Heme biosynthesis, glutamate => protoheme/siroheme	54.5	63.6	54.5	54.5	54.5	54.5	54.5	81.8	54.5	81.8	63.6	63.6	54.5	54.5	81.8	54.5	81.8	81.8	81.8	63.6	72.7	72.7	72.7	9.1	9.1	9.1
M00129	Ascorbate biosynthesis, animals, glucose-1P => ascorbate	16.7	33.3	16.7	16.7	16.7	16.7	0	33.3	16.7	33.3	16.7	16.7	16.7	16.7	16.7	16.7	16.7	16.7	16.7	16.7	0	0	16.7	33.3	0	0
M00114	Ascorbate biosynthesis, plants, glucose-6P => ascorbate	44.4	44.4	33.3	33.3	33.3	33.3	22.2	12.5	33.3	22.2	22.2	33.3	22.2	33.3	22.2	33.3	22.2	22.2	22.2	22.2	37.5	37.5	25	44.4	44.4	22.2
M00550	Ascorbate degradation, ascorbate => D-xylulose-5P	0	0	20	0	0	0	0	20	20	20	20	20	20	20	20	20	20	20	20	20	0	0	0	0	0	0
M00122	Cobalamin biosynthesis, cobinamide => cobalamin	37.5	37.5	50	50	50	50	50	50	50	50	50	50	50	50	50	50	50	50	50	0	50	37.5	37.5	50	50	12.5
M00117	Ubiquinone biosynthesis, prokaryotes, chorismate =>ubiquinone	33.3	44.4	11.1	22.2	11.1	22.2	11.1	22.2	11.1	33.3	33.3	22.2	22.2	22.2	11.1	22.2	11.1	11.1	22.2	11.1	33.3	22.2	33.3	22.2	11.1	22.2
M00116	Menaquinone biosynthesis, chorismate => menaquinone	11.1	33.3	66.7	66.7	66.7	66.7	66.7	66.7	66.7	66.7	66.7	66.7	66.7	66.7	66.7	66.7	66.7	66.7	66.7	66.7	22.2	22.2	22.2	11.1	0	11.1
M00118	Glutathione biosynthesis, glutamate => glutathione	0	0	0	0	0	0	0	0	0	0	0	0	0	0	0	0	0	0	0	0	0	0	0	0	0	0
M00140	C1-unit interconversion, prokaryotes	33.3	33.3	66.7	100	100	66.7	100	66.7	66.7	66.7	66.7	66.7	66.7	100	66.7	66.7	100	66.7	100	66.7	100	100	100	33.3	33.3	100
M00141	C1-unit interconversion, eukaryotes	50	50	50	50	50	50	50	50	50	50	50	50	50	50	50	50	50	50	50	50	50	50	50	50	50	50
M00133	Polyamine biosynthesis, arginine => agmatine => putrescine => spermidine	100	100	50	75	75	50	50	50	75	50	50	75	50	50	50	50	50	50	50	50	100	75	100	100	100	100
M00134	Polyamine biosynthesis, arginine => ornithine => putrescine	0	0	0	100	0	0	50	0	50	50	50	50	50	50	50	50	0	50	50	50	0	50	0	0	0	0
M00135	GABA biosynthesis, eukaryotes, putrescine => GABA	0	0	33.3	33.3	33.3	33.3	33.3	0	33.3	33.3	66.7	33.3	33.3	33.3	33.3	33.3	66.7	33.3	66.7	33.3	33.3	33.3	0	0	0	0
M00039	Lignin biosynthesis, cinnamate => lignin	20	20	20	20	0	20	20	20	20	20	20	20	20	20	20	20	20	20	20	20	0	0	0	0	0	0
M00033	Ectoine biosynthesis, aspartate => ectoine	40	40	60	40	40	40	60	40	60	60	60	60	60	60	60	60	40	40	60	40	40	40	60	40	40	0
Secondary metabolism																											
M00569	Catechol meta-cleavage, catechol => pyruvate + acetaldehyde	16.7	16.7	0	0	0	0	0	0	0	0	0	0	0	0	0	0	0	0	0	0	0	0	0	0	0	0
M00541	Benzoyl-CoA degradation, benzoyl-CoA => 3-hydroxypimeloyl-CoA	0	25	0	0	0	0	0	0	0	0	0	0	0	0	0	0	0	0	0	0	0	0	0	0	0	0
M00540	Benzoate degradation, cyclohexanecarboxylic acid => pimeloyl-CoA	0	20	0	0	0	0	0	0	0	0	0	0	0	0	0	0	0	0	0	0	0	0	0	0	0	0
M00623	Phthalate degradation, phthalate => protocatechuate	0	0	0	0	0	0	0	33.3	0	0	0	0	0	0	0	0	0	0	0	0	0	0	0	0	0	0

ID	Module name	Module completion ratio (%)																									
		afu	fpl	hsl	hma	hwa	nph	hla	hut	hmu	htu	nmg	hvo	hje	hbo	hxa	npe	nge	hru	nou	sali	tac	pto	fac	pfu	tko	abi
Structural complex																											
Energy metabolism																											
M00163	Photosystem I	0	0	0	0	0	0	0	0	0	0	0	0	0	0	0	0	0	0	0	0	0	0	0	0	0	0
M00144	NADH:quinone oxidoreductase, prokaryotes	71.4	78.6	76.9	84.6	76.9	76.9	76.9	92.3	76.9	84.6	76.9	84.6	84.6	84.6	84.6	76.9	76.9	76.9	84.6	76.9	78.6	78.6	71.4	50	71.4	57.1
M00145	NAD(P)H:quinone oxidoreductase, chloroplasts and cyanobacteria	0	0	0	9.1	9.1	9.1	0	9.1	9.1	9.1	0	9.1	0	9.1	9.1	9.1	0	0	9.1	0	0	0	0	0	0	0
M00149	Succinate dehydrogenase, prokaryotes	100	100	100	100	100	100	100	100	100	100	100	100	100	100	100	100	100	100	100	100	100	75	75	0	0	25
M00150	Fumarate reductase, prokaryotes	25	0	0	0	0	0	25	0	0	0	0	25	0	0	0	0	0	0	0	0	0	0	0	0	0	0
M00162	Cytochrome b6f complex	0	0	0	0	0	0	0	0	0	0	0	0	0	0	0	0	0	0	0	0	0	0	0	0	0	0
M00151	Cytochrome bc1 complex respiratory unit	0	0	0	0	0	0	0	0	0	0	0	0	0	0	0	0	0	0	0	0	0	0	0	0	0	0
M00152	Cytochrome bc1 complex	0	0	0	0	0	0	0	0	0	0	0	0	0	0	0	0	0	0	0	0	0	0	0	0	0	0
M00154	Cytochrome c oxidase	0	0	6.7	0	0	0	6.7	6.7	6.7	6.7	6.7	6.7	6.7	6.7	6.7	6.7	6.7	6.7	6.7	6.7	0	0	0	0	0	0
M00155	Cytochrome c oxidase, prokaryotes	50	50	100	100	100	100	100	100	100	100	100	100	100	100	100	100	100	100	100	100	0	100	100	0	0	0
M00153	Cytochrome d ubiquinol oxidase	50	0	100	0	0	0	0	100	100	100	100	100	100	100	0	100	100	0	0	100	100	50	100	0	0	0
M00416	Cytochrome aa3-600 menaquinol oxidase	0	0	0	0	0	0	0	0	0	0	0	0	0	0	0	0	0	0	0	0	0	0	0	0	0	0
M00157	F-type ATPase, prokaryotes and chloroplasts	0	0	0	0	0	0	0	0	0	0	0	0	0	0	0	0	0	0	0	0	0	0	0	0	0	0
M00159	V-type ATPase, prokaryotes	100	100	100	100	100	100	100	100	100	100	100	100	100	100	100	100	100	100	100	100	100	100	100	100	100	87.5
Carbohydrate & lipid metabolism																											
M00310	Pyruvate:ferredoxin oxidoreductase	100	100	0	0	0	0	0	100	0	0	0	0	0	0	0	0	0	0	0	0	75	0	0	75	75	100
M00311	2-oxoglutarate:ferredoxin oxidoreductase	100	75	50	50	50	50	50	50	50	50	50	50	50	50	50	50	50	50	50	50	50	50	50	100	100	100
M00312	2-oxoisovalerate:ferredoxin oxidoreductase	0	0	0	0	0	0	0	0	0	0	0	0	0	0	0	0	0	0	0	0	25	0	0	100	100	50
M00313	indolepyruvate:ferredoxin oxidoreductase	100	100	0	0	0	0	0	0	0	0	0	0	0	0	0	0	0	0	0	0	100	0	100	100	100	100
M00072	Oligosaccharyltransferase	16.7	16.7	16.7	16.7	16.7	16.7	16.7	16.7	16.7	16.7	16.7	16.7	16.7	16.7	16.7	16.7	16.7	16.7	16.7	16.7	16.7	16.7	16.7	16.7	16.7	16.7
Genetic information processing																											
M00260	DNA polymerase III complex, bacteria	16.7	16.7	0	0	0	0	0	0	0	0	0	0	0	0	0	0	0	0	0	0	16.7	0	0	0	0	0
M00264	DNA polymerase II complex, archaea	100	100	100	100	100	100	100	100	100	100	100	100	100	100	100	100	100	100	100	100	100	100	100	100	100	100
M00261	DNA polymerase alpha / primase complex	25	25	25	25	25	25	25	25	25	25	25	25	25	25	25	25	25	25	25	25	25	25	25	25	25	25
M00288	RPA complex	33.3	33.3	33.3	33.3	33.3	33.3	33.3	33.3	33.3	33.3	33.3	33.3	33.3	33.3	33.3	33.3	33.3	33.3	33.3	33.3	33.3	33.3	33.3	33.3	33.3	33.3
M00290	Holo-TFIIH complex	0	10	20	10	10	10	20	10	20	20	20	20	20	20	20	20	20	20	20	20	10	10	10	10	10	10

ID	Module name	Module completion ratio (%)																									
		afu	fpl	hsl	hma	hwa	nph	hla	hut	hmu	htu	nmg	hvo	hje	hbo	hxa	npe	nge	hru	nou	sali	tac	pto	fac	pfu	tko	abi
M00413	FA core complex	10	10	10	10	10	10	10	10	10	10	10	10	10	10	10	10	10	10	10	10	10	0	0	10	10	10
M00184	RNA polymerase, archaea	100	100	100	100	100	100	100	100	100	100	100	100	100	100	100	100	100	100	100	100	100	100	100	100	100	100
M00180	RNA polymerase II, eukaryotes	0	0	0	0	0	0	0	0	0	0	0	0	0	0	0	0	0	0	0	0	0	0	0	0	0	0
M00181	RNA polymerase III, eukaryotes	0	0	0	0	0	0	0	0	0	0	0	0	0	0	0	0	0	0	0	0	0	0	0	0	0	0
M00182	RNA polymerase I, eukaryotes	0	0	0	0	0	0	0	0	0	0	0	0	0	0	0	0	0	0	0	0	0	0	0	0	0	0
M00425	H/ACA ribonucleoprotein complex	50	50	25	25	25	25	25	25	25	25	25	25	25	25	25	25	25	25	25	25	25	25	50	50	50	50
M00390	Exosome, archaea	100	100	0	0	0	0	0	0	0	0	0	0	0	0	0	0	0	0	0	0	100	100	100	100	100	100
M00391	Exosome, eukaryotes	40	40	0	0	0	0	0	0	0	0	0	0	0	0	0	0	0	0	0	0	40	40	40	40	40	30
M00392	Ski complex	0	0	0	0	0	0	0	0	0	0	0	0	0	0	0	0	0	0	0	0	0	0	0	0	0	0
M00394	RNA degradosome	25	25	25	25	25	25	25	25	25	25	25	25	25	25	25	25	25	25	25	25	25	25	25	25	25	25
M00178	Ribosome, bacteria	59.6	59.6	59.6	59.6	59.6	59.6	59.6	59.6	59.6	59.6	59.6	59.6	59.6	59.6	59.6	59.6	59.6	59.6	59.6	59.6	59.6	59.6	57.7	59.6	59.6	59.6
M00179	Ribosome, archaea	100	100	96.6	93.2	96.6	98.3	96.6	98.3	98.3	96.6	96.6	96.6	96.6	96.6	98.3	98.3	98.3	96.6	96.6	98.3	96.6	93.2	91.5	100	100	100
M00177	Ribosome, eukaryotes	32.9	34.2	31.6	29.1	30.4	31.6	30.4	31.6	31.6	31.6	31.6	30.4	31.6	30.4	31.6	31.6	31.6	31.6	31.6	31.6	31.6	29.1	29.1	38	38	32.9
M00341	Proteasome, 19S regulatory particle (PA700)	0	0	0	0	0	0	0	0	0	0	0	0	0	0	0	0	0	0	0	0	0	0	0	0	0	0
M00342	Bacterial proteasome	50	50	50	50	50	50	50	50	50	50	50	50	50	50	50	50	50	50	50	50	50	50	50	50	50	50
M00343	Archaeal proteasome	100	100	100	100	100	100	100	100	100	100	100	100	100	100	100	100	100	100	100	100	66.7	66.7	66.7	100	100	100
M00401	Sec61 complex	33.3	33.3	33.3	33.3	33.3	33.3	33.3	33.3	33.3	33.3	33.3	33.3	33.3	33.3	33.3	33.3	33.3	33.3	33.3	33.3	33.3	33.3	33.3	33.3	33.3	33.3
Environmental information processing																											
M00189	Molybdate transport system	0	66.7	0	0	0	0	0	0	0	0	0	0	0	0	0	0	0	0	0	0	0	0	0	0	100	33.3
M00423	Molybdate/tungstate transport system	100	33.3	66.7	66.7	100	100	100	100	100	66.7	0	66.7	100	66.7	66.7	66.7	100	0	66.7	0	100	0	0	100	100	66.7
M00186	Tungstate transport system	0	100	0	100	0	100	100	0	0	100	100	100	100	100	100	100	100	0	100	0	0	0	0	0	0	0
M00188	NitT/TauT family transport system	100	100	0	0	100	0	100	0	0	100	33.3	100	100	100	100	33.3	0	0	100	0	66.7	33.3	66.7	0	0	0
M00190	Iron(III) transport system	0	0	100	100	100	100	100	100	100	33.3	100	100	100	100	100	100	100	100	100	100	0	0	0	0	0	0
M00191	Thiamine transport system	0	0	100	100	0	0	100	100	100	100	100	100	100	100	100	100	100	100	100	0	0	0	0	100	100	0
M00299	Spermidine/putrescine transport system	100	0	100	100	100	0	0	0	0	0	100	0	75	100	100	0	100	0	100	0	0	0	0	0	0	0
M00193	Putative spermidine/putrescine transport system	0	0	75	0	75	0	0	0	75	0	100	75	0	75	0	75	0	0	0	0	0	0	0	0	0	0
M00208	Glycine betaine/proline transport system	0	0	0	0	0	0	0	0	0	0	0	0	0	0	0	0	0	0	0	0	0	0	0	0	0	0
M00209	Osmoprotectant transport system	100	0	33.3	0	0	0	0	0	0	100	0	0	0	0	0	0	33.3	0	0	0	0	0	0	0	0	0

ID	Module name	Module completion ratio (%)																									
		afu	fpl	hsl	hma	hwa	nph	hla	hut	hmu	htu	nmg	hvo	hje	hbo	hxa	npe	nge	hru	nou	sali	tac	pto	fac	pfu	tko	abi
M00194	Maltose/maltodextrin transport system	0	0	25	25	25	0	25	0	25	25	25	25	25	25	25	25	25	25	25	25	25	25	0	25	25	0
M00196	Multiple sugar transport system	0	0	25	25	25	0	25	0	25	25	25	25	25	25	25	25	25	25	25	25	25	25	0	25	25	0
M00203	Glucose/arabinose transport system	0	0	0	0	0	0	0	0	0	0	0	0	0	0	0	0	0	0	0	0	0	100	100	0	0	0
M00605	Glucose/mannose transport system	0	0	25	100	25	0	100	25	100	100	25	100	100	100	100	25	100	25	25	100	25	25	0	25	25	0
M00604	Trehalose transport system	0	0	0	0	0	0	0	0	0	0	0	0	0	0	0	0	0	0	0	0	100	100	0	0	0	0
M00206	Cellobiose transport system	0	0	25	25	25	0	25	0	25	25	25	25	25	25	25	25	25	25	25	25	25	25	0	25	25	0
M00606	N,N'-Diacetylchitobiose transport system	0	0	25	25	25	0	25	0	25	25	25	25	25	25	25	25	25	25	25	25	25	25	0	25	25	0
M00212	Ribose transport system	0	0	0	0	0	0	0	0	0	0	0	0	66.7	0	0	0	0	0	0	0	0	0	0	0	0	0
M00599	Inositol-phosphate transport system	0	0	0	0	0	0	0	0	0	0	0	0	0	0	0	0	0	0	0	0	0	0	0	100	0	0
M00197	Putative fructooligosaccharide transport system	0	0	25	25	25	0	25	0	25	25	25	25	25	25	25	25	25	25	25	25	25	25	0	25	25	0
M00198	Putative sn-glycerol-phosphate transport system	0	0	75	75	0	0	0	0	75	0	0	75	0	75	75	0	0	0	0	0	0	0	0	0	0	0
M00200	Putative sorbitol/mannitol transport system	0	0	25	25	25	0	25	0	25	25	25	25	25	25	25	25	25	25	25	25	25	25	0	25	25	0
M00602	Arabinosaccharide transport system	0	0	25	25	25	0	25	0	25	25	25	25	25	25	25	25	25	25	25	25	25	25	0	25	25	0
M00491	arabinogalactan oligomer/maltooligosaccharide transport system	0	0	25	100	25	0	100	0	100	100	100	100	25	100	100	25	25	25	25	25	100	25	0	100	100	0
M00207	Putative multiple sugar transport system	0	0	25	100	75	0	100	0	100	75	100	75	75	100	100	100	100	100	100	100	75	100	0	100	25	0
M00221	Putative simple sugar transport system	66.7	0	66.7	100	66.7	0	66.7	0	66.7	0	0	66.7	66.7	66.7	100	0	0	0	66.7	66.7	0	0	0	66.7	66.7	0
M00211	Putative ABC transport system (Saccharide and polyol_2)	0	0	0	0	0	0	0	0	0	100	0	0	0	0	0	0	0	0	100	0	0	0	0	0	0	0
M00222	Phosphate transport system	100	100	100	100	100	100	100	100	100	100	100	100	100	100	100	100	100	100	100	100	0	0	0	100	100	0
M00223	Phosphonate transport system	0	0	100	100	100	100	100	0	0	100	100	100	0	100	100	100	100	0	100	0	0	0	0	0	0	0
M00237	Branched-chain amino acid transport system	100	100	20	100	100	100	100	0	100	100	100	100	100	100	20	100	100	100	100	100	0	0	0	0	0	0
M00236	Putative polar amino acid transport system	100	0	0	100	100	100	100	0	100	100	0	100	0	100	100	0	0	0	100	0	0	0	0	0	0	33.3
M00439	Oligopeptide transport system	0	0	0	0	0	0	0	0	0	0	0	0	0	0	0	0	0	0	0	0	0	0	0	0	0	0
M00239	Peptides/nickel transport system	100	100	100	100	100	100	100	100	100	100	100	100	100	100	100	100	100	100	100	80	100	100	100	100	100	100
M00240	Iron complex transport system	100	100	100	100	100	100	100	100	100	100	100	100	100	100	100	100	100	100	100	100	100	100	100	100	100	0
M00242	Zinc transport system	0	0	100	100	0	100	100	100	100	100	100	100	100	100	100	100	100	100	100	0	0	0	0	0	0	0
M00318	Iron/zinc/copper transport system	0	0	0	0	0	0	0	0	0	0	0	0	0	0	0	0	0	0	0	0	0	0	0	0	0	0
M00317	Manganese/iron transport system	0	0	0	0	0	0	0	0	0	0	0	0	0	0	0	0	0	0	0	0	0	0	0	0	0	0
M00319	Manganese/zinc/iron transport system	0	0	0	0	0	0	0	0	0	0	0	0	0	0	0	0	0	0	0	0	0	0	0	0	0	0

ID	Module name	Module completion ratio (%)																									
		afu	fpl	hsl	hma	hwa	nph	hla	hut	hmu	htu	nmg	hvo	hje	hbo	hxa	npe	nge	hru	nou	sali	tac	pto	fac	pfu	tko	abi
M00243	Manganese/iron transport system	0	0	0	0	0	0	0	0	0	0	0	0	0	0	0	0	0	0	0	0	0	0	0	0	0	0
M00245	Cobalt/nickel transport system	100	100	100	75	100	0	0	75	100	0	0	100	0	100	0	0	0	0	0	0	0	0	0	100	100	100
M00246	Nickel transport system	100	100	100	66.7	100	0	0	100	100	0	0	100	0	100	0	0	0	0	0	0	0	0	0	100	100	100
M00581	Biotin transport system	33.3	33.3	100	100	100	100	100	0	100	100	100	100	100	100	100	100	100	100	100	33.3	0	0	0	33.3	33.3	0
M00582	Energy-coupling factor transport system	100	100	25	25	25	25	25	0	25	25	25	100	25	25	100	25	25	25	25	25	0	0	0	100	100	100
M00244	Putative zinc/manganese transport system	100	0	0	0	0	0	0	0	0	0	0	0	0	0	0	0	0	0	0	0	0	0	0	100	100	100
M00298	Multidrug/hemolysin transport system	0	0	0	0	0	0	0	0	0	0	0	0	0	0	0	0	0	0	0	0	0	0	0	0	0	0
M00250	Lipopolysaccharide transport system	100	0	0	0	0	0	0	0	0	0	0	0	0	0	0	0	0	0	0	0	0	0	0	0	0	0
M00320	Lipopolysaccharide export system	0	33.3	0	0	0	0	0	0	0	0	0	0	0	0	0	0	0	0	0	0	0	0	0	0	0	0
M00252	Lipooligosaccharide transport system	0	0	0	0	0	0	0	0	0	0	0	0	0	0	0	0	0	0	0	0	0	0	0	0	0	0
M00224	Fluoroquinolones transport system	0	0	0	0	0	0	0	0	0	0	0	0	0	0	0	0	0	0	0	0	0	0	0	100	100	0
M00255	Lipoprotein-releasing system	0	0	0	0	0	0	0	0	0	0	0	0	0	0	0	0	0	0	0	0	0	0	0	0	0	0
M00259	Heme transport system	100	100	0	100	0	100	0	0	0	0	0	0	0	0	0	0	100	0	0	0	0	0	0	0	0	0
M00258	Uncharacterized ABC transport system_2	100	100	100	100	100	100	100	50	100	100	100	100	50	100	100	100	100	100	100	100	0	0	0	0	50	100
M00254	Uncharacterized ABC transport system_3	100	100	100	100	100	100	100	100	100	100	100	100	100	100	100	100	100	100	100	100	100	100	100	100	100	100
M00273	PTS system, fructose-specific II component	0	0	0	100	0	0	0	0	0	100	0	100	100	0	0	0	0	0	0	0	0	0	0	0	0	0
M00279	PTS system, galactitol-specific II component	0	0	0	0	0	0	0	0	0	0	0	100	0	0	0	0	0	0	0	0	0	0	0	0	0	0
M00276	PTS system, mannose-specific II component	0	0	0	0	0	0	0	0	0	0	0	0	0	0	0	0	0	0	0	0	0	0	0	0	0	0
M00334	Type VI secretion system	0	0	0	0	0	0	0	0	0	0	0	0	0	0	0	0	0	0	0	0	0	0	0	0	0	0
M00335	Sec (secretion) system	25	25	44.4	44.4	44.4	44.4	44.4	44.4	44.4	44.4	44.4	44.4	44.4	44.4	44.4	44.4	44.4	44.4	44.4	44.4	25	25	25	44.4	44.4	25

ID	Module name	Module completion ratio (%)																									
		afu	fpl	hsl	hma	hwa	nph	hla	hut	hmu	htu	nmg	hvo	hje	hbo	hxa	npe	nge	hru	nou	sali	tac	pto	fac	pfu	tko	abi
Functional set																											
Metabolism																											
M00360	Aminoacyl-tRNA biosynthesis, prokaryotes	100	100	100	100	100	100	100	100	100	100	100	100	100	100	100	100	100	100	100	100	100	100	100	100	100	100
M00359	Aminoacyl-tRNA biosynthesis, eukaryotes	85	85	85	85	85	85	85	85	85	85	85	85	85	85	85	85	90	85	85	85	90	90	90	90	90	95
M00362	Nucleotide sugar biosynthesis, prokaryotes	42.9	71.4	57.1	71.4	57.1	57.1	28.6	57.1	71.4	42.9	57.1	71.4	57.1	71.4	57.1	57.1	57.1	42.9	42.9	42.9	28.6	28.6	28.6	100	71.4	28.6
M00361	Nucleotide sugar biosynthesis, eukaryotes	33.3	50	50	50	50	50	16.7	33.3	50	33.3	33.3	50	33.3	50	33.3	50	33.3	33.3	33.3	33.3	33.3	33.3	33.3	66.7	33.3	16.7
Environmental information processing																											
M00454	KdpD-KdpE (potassium transport) two-component regulatory system	0	0	0	0	0	0	0	0	0	0	0	0	0	0	0	0	0	0	0	0	0	0	0	0	0	0
M00459	VicK-VicR (cell wall metabolism) two-component regulatory system	0	0	0	0	0	0	0	0	0	0	0	0	0	0	0	0	0	0	0	0	0	0	0	0	0	0
M00460	MprB-MprA (maintenance of persistent infection two-component regulatory system	0	0	0	0	0	0	0	50	0	0	0	0	0	0	0	0	0	0	0	0	0	0	0	0	0	0
M00506	CheA-CheYBV (chemotaxis) two-component regulatory system	60	60	60	60	0	60	60	60	60	60	60	60	20	60	60	60	60	60	0	60	0	0	0	0	60	20
M00507	ChpA-ChpB/PilGH (chemosensory) two-component regulatory system	0	0	0	0	0	0	0	0	0	0	0	0	0	0	0	0	0	0	0	0	0	0	0	0	0	0
M00511	PleC-PleD (cell fate control) two-component regulatory system	0	0	0	0	0	0	0	0	0	0	0	0	0	0	0	0	0	0	0	0	0	0	0	0	0	0
M00520	ChvG-ChvI (acidity sensing) two-component regulatory system	0	0	0	0	0	0	0	0	0	0	0	0	0	0	0	0	0	0	0	0	0	0	0	0	0	0

Full species name of each abbreviation is described in Table S2.

Table S3 Module completion patterns in 79 archaeal species listed in Table S2 (continied: *Crenarchaeota*).

ID	Module name	Module completion ratio (%)																			
		ape	smr	iho	dmu	tag	iag	thg	hbu	pfm	sai	mse	aho	pis	cma	ttn	vdi	tpe	asc	clg	ffo
Pathway module																					
Energy metabolism																					
M00165	Reductive pentose phosphate cycle (Calvin cycle)	45.5	36.4	45.5	36.4	36.4	36.4	36.4	36.4	45.5	45.5	45.5	45.5	45.5	45.5	45.5	45.5	36.4	27.3	45.5	27.3
M00166	Reductive pentose phosphate cycle, ribulose-5P => glyceraldehyde-3P	50	75	50	75	75	25	75	75	50	50	50	50	50	50	50	50	75	50	50	50
M00167	Reductive pentose phosphate cycle, glyceraldehyde-3P => ribulose-5P	42.9	14.3	42.9	14.3	14.3	42.9	14.3	14.3	42.9	42.9	42.9	42.9	42.9	42.9	42.9	42.9	14.3	14.3	42.9	14.3
M00168	CAM (Crassulacean acid metabolism), dark	50	0	100	0	0	50	0	0	100	100	100	100	100	100	100	100	50	50	50	0
M00169	CAM (Crassulacean acid metabolism), light	0	0	0	0	0	50	0	0	0	0	50	0	0	50	50	0	0	50	0	50
M00172	C4-dicarboxylic acid cycle, NADP - malic enzyme type	0	0	25	0	0	50	0	0	25	25	50	25	25	50	50	25	25	25	0	25
M00171	C4-dicarboxylic acid cycle, NAD - malic enzyme type	0	0	14.3	0	0	28.6	0	0	14.3	14.3	14.3	14.3	14.3	28.6	28.6	14.3	14.3	14.3	0	14.3
M00170	C4-dicarboxylic acid cycle, phosphoenolpyruvate carboxykinase type	25	0	25	0	0	25	0	0	25	25	25	25	25	25	25	25	25	0	0	25
M00173	Reductive citrate cycle (Arnon-Buchanan cycle)	72.7	36.4	81.8	36.4	36.4	45.5	40	40	72.7	90.9	90.9	90.9	90.9	90.9	90.9	90.9	45.5	81.8	81.8	36.4
M00376	3-Hydroxypropionate bi-cycle	18.8	0	12.5	0	0	0	0	12.5	12.5	52.6	52.6	52.6	16.7	16.7	16.7	27.8	0	18.8	18.8	0
M00375	Hydroxypropionate-hydroxybutylate cycle	25	0	33.3	8.3	8.3	8.3	8.3	0	33.3	100	100	93.3	50	28.6	50	28.6	8.3	25	33.3	8.3
M00374	Dicarboxylate-hydroxybutyrate cycle	53.8	23.1	84.6	30.8	30.8	23.1	30.8	30.8	84.6	100	100	92.3	100	69.2	100	69.2	38.5	61.5	69.2	23.1
M00377	Reductive acetyl-CoA pathway (Wood-Ljungdahl pathway)	0	0	0	0	0	0	0	0	0	0	0	0	14.3	0	14.3	0	0	0	0	0
M00579	Phosphate acetyltransferase-acetate kinase pathway, acetyl-CoA => acetate	0	0	0	0	0	0	0	0	0	0	0	0	0	0	0	0	0	0	0	0
M00175	Nitrogen fixation, nitrogen => ammonia	0	0	0	0	0	0	0	0	0	0	0	0	0	0	0	0	0	0	0	0
M00531	Assimilatory nitrate reduction, nitrate => ammonia	0	0	0	0	0	0	0	0	0	0	100	0	0	50	0	50	0	0	0	0
M00530	Dissimilatory nitrate reduction, nitrate => ammonia	0	0	0	0	0	0	0	0	0	0	0	0	0	0	0	50	0	0	0	0
M00529	Denitrification, nitrate => nitrogen	0	0	0	0	0	0	0	0	0	0	0	0	0	0	0	25	0	0	0	0
M00528	Nitrification, ammonia => nitrite	0	0	0	0	0	0	0	0	0	0	0	0	0	0	0	0	0	0	0	0
M00567	Methanogenesis, CO2 => methane	0	0	0	0	0	0	0	0	0	12.5	12.5	12.5	0	0	0	0	0	0	0	0
M00357	Methanogenesis, acetate => methane	20	0	20	0	0	0	0	0	20	40	40	40	20	20	20	20	20	20	20	0
M00356	Methanogenesis, methanol => methane	0	0	0	0	0	0	0	0	0	33.3	33.3	33.3	0	0	0	0	0	0	0	0
M00563	Methanogenesis, mono-/di-/tri-methylamine => methane	0	0	0	0	0	0	0	0	0	25	25	25	0	0	0	0	0	0	0	0
M00358	Coenzyme M biosynthesis	0	0	0	0	0	0	0	0	0	0	0	0	0	0	0	0	0	0	0	0
M00174	Methane oxidation, methanotroph, methane => formaldehyde	0	0	0	0	0	0	0	0	0	0	0	0	0	0	0	0	0	0	0	0
M00346	Formaldehyde assimilation, serine pathway	44.4	33.3	44.4	22.2	22.2	44.4	22.2	33.3	44.4	55.6	55.6	55.6	44.4	55.6	44.4	55.6	33.3	44.4	44.4	22.2

ID	Module name	Module completion ratio (%)																			
		ape	smr	iho	dmu	tag	iag	thg	hbu	pfm	sai	mse	aho	pis	cma	ttn	vdi	tpe	asc	clg	ffo
M00345	Formaldehyde assimilation, ribulose monophosphate pathway	50	50	50	50	50	50	50	50	50	50	50	50	50	75	75	75	50	50	50	50
M00344	Formaldehyde assimilation, xylulose monophosphate pathway	0	0	0	0	0	0	0	0	0	0	0	0	0	0	0	0	0	0	0	0
M00378	F420 biosynthesis	0	0	0	0	0	0	0	0	0	0	0	0	0	0	0	0	0	20	0	0
M00422	Acetyl-CoA pathway, CO2 => acetyl-CoA	0	0	0	0	0	0	0	0	0	0	0	0	0	0	0	0	0	0	0	0
M00176	Assimilatory sulfate reduction, sulfate => H2S	33.3	66.7	66.7	33.3	33.3	33.3	33.3	66.7	66.7	33.3	33.3	0	66.7	0	33.3	33.3	33.3	33.3	33.3	0
M00596	Dissimilatory sulfate reduction, sulfate => H2S	33.3	33.3	33.3	0	0	0	0	33.3	33.3	33.3	33.3	0	66.7	100	100	100	0	33.3	33.3	0
Carbohydrate & lipid metabolism																					
M00001	Glycolysis (Embden-Meyerhof pathway), glucose => pyruvate	90	90	70	90	90	88.9	90	70	60	70	80	80	80	100	100	100	90	90	90	70
M00002	Glycolysis, core module involving three-carbon compounds	100	100	83.3	100	100	100	100	100	83.3	100	100	100	100	100	100	100	100	100	100	100
M00003	Gluconeogenesis, oxaloacetate => fructose-6P	100	62.5	62.5	100	100	50	100	100	62.5	100	100	62.5	62.5	100	62.5	75	100	75	75	100
M00307	Pyruvate oxidation, pyruvate => acetyl-CoA	100	100	100	100	100	100	100	100	100	100	100	100	100	100	100	100	100	100	100	100
M00009	Citrate cycle (TCA cycle, Krebs cycle)	87.5	12.5	62.5	25	25	12.5	37.5	37.5	75	75	75	87.5	75	87.5	75	100	12.5	87.5	87.5	12.5
M00010	Citrate cycle, first carbon oxidation, oxaloacetate => 2-oxoglutarate	100	0	33.3	0	0	33.3	33.3	0	66.7	100	100	100	100	100	100	100	0	100	100	0
M00011	Citrate cycle, second carbon oxidation, 2-oxoglutarate => oxaloacetate	80	20	80	40	40	0	40	60	80	60	60	80	60	80	60	100	20	80	80	20
M00004	Pentose phosphate pathway (Pentose phosphate cycle)	42.9	28.6	42.9	28.6	28.6	42.9	28.6	28.6	42.9	42.9	42.9	42.9	42.9	42.9	42.9	42.9	28.6	28.6	42.9	28.6
M00006	Pentose phosphate pathway, oxidative phase, glucose 6P => ribulose 5P	0	0	0	0	0	0	0	0	0	0	0	0	0	0	0	0	0	0	0	0
M00007	Pentose phosphate pathway, non-oxidative phase,fructose 6P => ribose 5P	50	25	50	25	25	50	25	25	50	50	50	50	50	50	50	50	25	25	50	25
M00580	Pentose phosphate pathway, archaea, fructose 6P => ribose 5P	100	100	100	100	100	100	100	100	100	100	100	100	100	100	100	100	100	100	100	100
M00005	PRPP biosynthesis, ribose 5P => PRPP	100	100	100	100	100	100	100	100	100	100	100	100	100	100	100	100	100	100	100	100
M00008	Entner-Doudoroff pathway, glucose-6P => glyceraldehyde-3P + pyruvate	0	0	0	0	0	0	0	0	0	0	0	0	0	0	0	0	0	0	0	0
M00308	Semi-phosphorylative Entner-Doudoroff pathway, gluconate => glyceraldehyde-3P + pyruvate	25	25	20	25	25	25	25	20	20	50	50	40	20	50	50	50	20	25	25	25
M00633	Semi-phosphorylative Entner-Doudoroff pathway, gluconate/galactonate => glycerate-3P	25	25	0	25	25	25	25	0	0	100	100	50	0	75	100	75	0	25	25	25
M00309	Non-phosphorylative Entner-Doudoroff pathway, gluconate => glyceraldehyde + pyruvate	33.3	33.3	33.3	33.3	33.3	0	33.3	33.3	33.3	100	100	100	33.3	66.7	100	66.7	33.3	33.3	33.3	33.3
M00012	Glyoxylate cycle	60	0	20	0	0	0	0	0	40	100	80	60	80	60	80	60	0	60	60	0
M00373	Ethylmalonyl pathway	7.1	0	7.1	7.1	7.1	7.1	7.1	0	7.1	21.4	21.4	21.4	7.1	7.1	7.1	14.3	7.1	7.1	7.1	7.1
M00532	Photorespiration	20	40	10	40	40	20	40	30	20	20	20	20	20	20	10	30	10	20	20	30
M00013	Malonate semialdehyde pathway, propanoyl-CoA => Acetyl-CoA	25	0	0	0	0	0	0	0	0	0	25	0	25	25	0	25	0	25	25	0
M00632	Galactose degradation, Leloir pathway, galactose => alpha-D-glucose-1P	0	75	25	25	25	75	25	0	0	25	25	25	25	75	25	75	75	75	75	25

ID	Module name	Module completion ratio (%)																			
		ape	smr	iho	dmu	tag	iag	thg	hbu	pfm	sai	mse	aho	pis	cma	ttn	vdi	tpe	asc	clg	ffo
M00552	D-galactonate degradation, De Ley-Doudoroff pathway, D-galactonate => glycerate-3P	20	20	20	20	20	0	20	20	20	20	20	40	20	40	20	20	20	20	20	20
M00014	Glucuronate pathway (uronate pathway)	14.3	0	0	0	0	14.3	0	0	0	14.3	14.3	0	14.3	0	0	0	28.6	0	0	0
M00631	D-Galacturonate degradation (bacteria), D-galacturonate => pyruvate + D-glyceraldehyde 3P	0	0	0	0	0	20	0	0	0	0	0	0	0	40	0	20	0	0	20	0
M00061	Uronic acid metabolism	0	0	0	0	0	20	0	0	0	0	0	20	0	40	0	0	0	0	0	0
M00081	Pectin degradation	0	0	0	0	0	0	0	0	0	0	0	0	0	0	0	0	0	0	0	0
M00082	Fatty acid biosynthesis, initiation	0	0	0	0	0	0	0	0	0	0	0	0	0	0	0	0	0	0	0	0
M00113	Jasmonic acid biosynthesis	12.5	12.5	0	0	0	0	0	12.5	0	12.5	12.5	0	12.5	0	0	0	0	0	0	0
M00086	beta-Oxidation, acyl-CoA synthesis	100	0	0	0	0	0	0	0	100	100	100	100	100	0	100	100	0	100	100	0
M00087	beta-Oxidation	66.7	33.3	0	0	0	0	0	33.3	0	33.3	66.7	0	66.7	33.3	0	33.3	0	33.3	0	0
M00088	Ketone body biosynthesis, acetyl-CoA => acetoacetate/3-hydroxybutyrate/ acetone	40	20	40	40	40	40	40	20	40	40	40	40	40	40	40	60	40	40	40	40
M00093	Phosphatidylethanolamine (PE) biosynthesis, PA => PS => PE	0	0	0	0	0	0	0	0	0	0	0	0	0	0	0	0	0	0	0	33.3
M00131	Inositol phosphate metabolism, Ins(1,3,4,5)P4 => Ins(1,3,4)P3 => myo-inositol	25	25	25	25	25	25	25	25	25	25	25	25	25	25	25	25	25	25	25	25
M00094	Ceramide biosynthesis	0	0	0	0	0	0	0	0	0	0	0	0	0	0	0	0	0	0	0	0
M00099	Sphingosine biosynthesis	0	0	0	0	0	0	0	0	0	0	0	0	0	0	0	0	0	0	0	0
M00060	Lipopolysaccharide biosynthesis, KDO2-lipid A	0	0	0	0	0	0	0	0	0	0	0	0	0	0	0	0	0	0	0	0
M00064	ADP-L-glycero-D-manno-heptose biosynthesis	0	0	0	0	0	0	0	0	0	0	0	0	0	0	0	0	0	0	0	0
M00080	Lipopolysaccharide biosynthesis, inner core => outer core => O-antigen	0	0	0	0	0	0	0	0	0	0	0	0	0	0	0	0	0	0	0	0
M00549	Nucleotide sugar biosynthesis, glucose => UDP-glucose	33.3	66.7	33.3	33.3	33.3	66.7	33.3	0	0	0	33.3	0	66.7	66.7	33.3	66.7	66.7	66.7	66.7	0
M00554	Nucleotide sugar biosynthesis, galactose => UDP-galactose	0	100	0	0	0	100	0	0	0	0	0	0	0	100	0	100	100	100	100	0
M00565	Trehalose biosynthesis, D-glucose 1P => trehalose	0	40	0	40	40	40	40	0	0	60	40	40	40	20	40	20	40	40	40	0
M00055	N-glycan precursor biosynthesis	0	7.7	7.7	7.7	7.7	7.7	7.7	7.7	7.7	7.7	7.7	7.7	7.7	7.7	7.7	7.7	7.7	7.7	7.7	7.7
M00076	Dermatan sulfate degradation	0	0	0	0	0	20	0	0	0	0	0	0	0	20	0	0	0	0	0	0
M00077	Chondroitin sulfate degradation	0	0	0	0	0	25	0	0	0	0	0	0	0	25	0	0	0	0	0	0
M00078	Heparan sulfate degradation	0	0	0	0	0	12.5	0	0	0	0	0	0	0	12.5	0	0	0	0	0	0
M00095	C5 isoprenoid biosynthesis, mevalonate pathway	71.4	57.1	71.4	57.1	57.1	57.1	57.1	57.1	71.4	100	100	100	71.4	71.4	71.4	71.4	57.1	71.4	71.4	57.1
M00096	C5 isoprenoid biosynthesis, non-mevalonate pathway	12.5	12.5	12.5	12.5	12.5	12.5	12.5	12.5	12.5	12.5	12.5	12.5	12.5	12.5	12.5	12.5	12.5	12.5	12.5	12.5
M00364	C10-C20 isoprenoid biosynthesis, bacteria	100	100	100	100	100	100	100	100	100	100	100	100	100	100	100	100	100	100	100	100
M00365	C10-C20 isoprenoid biosynthesis, archaea	100	100	100	100	100	100	100	100	100	100	100	100	100	100	100	100	100	100	100	100

ID	Module name	Module completion ratio (%)																			
		ape	smr	iho	dmu	tag	iag	thg	hbu	pfm	sai	mse	aho	pis	cma	ttn	vdi	tpe	asc	clg	ffo
M00366	C10-C20 isoprenoid biosynthesis, plants	25	25	25	25	25	25	25	25	25	25	25	25	25	25	25	25	25	25	25	25
M00367	C10-C20 isoprenoid biosynthesis, non-plant eukaryotes	33.3	33.3	33.3	33.3	33.3	33.3	33.3	33.3	33.3	33.3	33.3	33.3	33.3	33.3	33.3	33.3	33.3	33.3	33.3	33.3
M00097	beta-Carotene biosynthesis, GGAP => beta-carotene	0	0	0	0	0	0	0	0	0	16.7	16.7	0	0	0	0	0	0	0	0	0
M00372	Abscisic acid biosynthesis, beta-carotene => abscisic acid	0	0	0	0	0	0	0	0	0	16.7	16.7	0	0	0	0	0	0	0	0	0
Nucleotide & amino acid metabolism																					
M00048	Inosine monophosphate biosynthesis, PRPP + glutamine => IMP	0	10	81.8	0	0	81.8	0	0	81.8	90.9	90.9	90.9	90.9	90.9	90.9	90.9	0	0	0	81.8
M00049	Adenine ribonucleotide biosynthesis, IMP => ADP, ATP	50	50	100	50	50	100	50	50	100	100	100	100	100	100	100	100	50	50	50	100
M00050	Guanine ribonucleotide biosynthesis IMP => GDP, GTP	75	25	50	50	50	75	25	25	50	50	50	50	50	75	50	50	50	75	75	50
M00546	Purine degradation, xanthine => urea	0	20	0	0	0	0	0	0	0	0	0	0	0	0	0	0	0	0	0	0
M00051	Uridine monophosphate biosynthesis, glutamine (+ PRPP) => UMP	83.3	83.3	100	80	0	100	75	100	100	100	100	100	100	100	100	100	83.3	83.3	100	83.3
M00052	Pyrimidine ribonucleotide biosynthesis, UMP => UDP/UTP, CDP/CTP	100	100	100	66.7	66.7	66.7	100	100	100	100	100	100	100	100	100	100	66.7	100	100	100
M00053	Pyrimidine deoxyribonuleotide biosynthesis, CDP/CTP => dCDP/dCTP, dTDP/dTTP	62.5	75	62.5	37.5	37.5	62.5	62.5	75	75	62.5	50	50	75	50	75	62.5	37.5	62.5	62.5	62.5
M00046	Pyrimidine degradation, uracil => beta-alanine, thymine => 3-aminoisobutanoate	0	0	0	33.3	33.3	0	33.3	0	0	0	0	0	0	0	0	0	0	0	0	0
M00020	Serine biosynthesis, glycerate-3P => serine	33.3	33.3	66.7	66.7	66.7	66.7	66.7	33.3	66.7	33.3	33.3	33.3	66.7	33.3	66.7	66.7	0	33.3	66.7	33.3
M00018	Threonine biosynthesis, aspartate => homoserine => threonine	100	20	80	20	20	100	100	60	80	100	100	100	100	100	100	100	20	20	100	20
M00555	Betaine biosynthesis, choline => betaine	0	0	0	0	0	0	0	0	0	50	0	0	0	0	0	0	0	0	0	0
M00021	Cysteine biosynthesis, serine => cysteine	0	50	50	50	50	0	50	50	50	50	50	50	50	50	50	50	50	0	0	0
M00338	Cysteine biosynthesis, homocysteine + serine => cysteine	100	0	0	50	50	50	50	50	50	0	0	0	50	0	0	0	50	50	50	0
M00609	Cysteine biosynthesis, methionine => cysteine	16.7	16.7	16.7	16.7	16.7	16.7	16.7	16.7	16.7	16.7	16.7	16.7	16.7	16.7	16.7	16.7	16.7	16.7	33.3	16.7
M00017	Methionine biosynthesis, apartate => homoserine => methionine	71.4	14.3	57.1	0	0	71.4	71.4	42.9	57.1	71.4	71.4	71.4	71.4	71.4	71.4	71.4	14.3	28.6	71.4	0
M00034	Methionine salvage pathway	50	62.5	62.5	62.5	62.5	62.5	62.5	62.5	62.5	50	62.5	50	50	50	50	50	62.5	62.5	62.5	62.5
M00035	Methionine degradation	100	75	75	100	100	100	100	100	100	75	75	75	100	75	75	75	100	100	100	75
M00368	Ethylene biosynthesis, methionine => ethylene	33.3	33.3	33.3	33.3	33.3	33.3	33.3	33.3	33.3	33.3	33.3	33.3	33.3	33.3	33.3	33.3	33.3	33.3	33.3	33.3
M00019	Valine/isoleucine biosynthesis, pyruvate => valine/2-oxobutanoate => isoleucine	25	0	75	0	0	75	0	0	75	50	50	50	75	75	100	100	0	25	50	0
M00535	Isoleucine biosynthesis, pyruvate => 2-oxobutanoate	0	0	66.7	0	0	66.7	33.3	0	66.7	66.7	66.7	66.7	66.7	66.7	66.7	66.7	0	0	0	0
M00570	Isoleucine biosynthesis, threonine => 2-oxobutanoate => isoleucine	40	20	60	0	0	80	0	20	60	60	60	60	80	80	100	100	20	40	60	20
M00432	Leucine biosynthesis, 2-oxoisovalerate => 2-oxoisocaproate	0	0	100	0	0	100	33.3	0	100	100	100	100	100	100	100	100	0	0	33.3	0
M00036	Leucine degradation, leucine => acetoacetate + acetyl-CoA	16.7	0	0	0	0	16.7	0	0	0	0	16.7	0	16.7	16.7	16.7	33.3	0	16.7	0	0

ID	Module name	Module completion ratio (%)																			
		ape	smr	iho	dmu	tag	iag	thg	hbu	pfm	sai	mse	aho	pis	cma	ttn	vdi	tpe	asc	clg	ffo
M00016	Lysine biosynthesis, succinyl-DAP pathway, aspartate => lysine	44.4	22.2	33.3	11.1	11.1	44.4	33.3	33.3	33.3	44.4	44.4	44.4	44.4	66.7	44.4	66.7	22.2	22.2	55.6	44.4
M00525	Lysine biosynthesis, acetyl-DAP pathway, aspartate => lysine	33.3	11.1	22.2	0	0	33.3	22.2	22.2	22.2	33.3	33.3	33.3	33.3	55.6	33.3	55.6	11.1	11.1	44.4	33.3
M00526	Lysine biosynthesis, DAP dehydrogenase pathway, aspartate => lysine	50	16.7	33.3	0	0	50	33.3	33.3	33.3	50	50	50	50	83.3	50	83.3	16.7	16.7	66.7	50
M00527	Lysine biosynthesis, DAP aminotransferase pathway, aspartate => lysine	42.9	14.3	28.6	0	0	42.9	28.6	28.6	28.6	42.9	42.9	42.9	42.9	71.4	42.9	71.4	14.3	14.3	57.1	42.9
M00030	Lysine biosynthesis, AAA pathway, 2-oxoglutarate => 2-aminoadipate => lysine	0	0	0	0	0	0	0	0	0	0	0	0	0	0	0	0	0	0	0	0
M00433	Lysine biosynthesis, 2-oxoglutarate => 2-oxoadipate	0	0	0	0	0	0	0	0	0	0	0	0	0	0	0	0	0	0	0	0
M00031	Lysine biosynthesis, 2-aminoadipate => lysine	100	0	100	0	0	100	0	0	100	100	100	100	100	100	100	100	0	0	0	0
M00032	Lysine degradation, lysine => saccharopine => acetoacetyl-CoA	0	0	0	0	0	0	0	0	0	0	0	0	0	0	0	0	0	0	0	0
M00608	2-oxocarboxylic acid chain extension, 2-oxoglutarate =>2-oxoadipate => 2-oxopimelate => 2-oxosuberate	0	0	0	0	0	0	0	0	0	0	0	0	0	0	0	0	0	0	0	0
M00015	Proline biosynthesis, glutamate => proline	50	0	50	50	0	50	50	50	50	0	50	50	50	50	50	50	50	50	50	0
M00028	Ornithine biosynthesis, glutamate => ornithine	60	0	60	0	0	60	0	0	60	60	60	60	60	60	60	60	0	0	0	0
M00029	Urea cycle	20	20	60	20	20	60	20	60	60	60	60	60	60	60	60	60	20	20	20	20
M00026	Histidine biosynthesis, PRPP => histidine	14.3	0	88.9	0	0	88.9	14.3	14.3	77.8	88.9	88.9	88.9	88.9	88.9	88.9	88.9	0	0	0	0
M00045	Histidine degradation, histidine => N-formiminoglutamate => glutamate	0	0	0	0	0	0	0	0	0	0	0	0	0	75	0	75	0	75	75	0
M00022	Shikimate pathway, phosphoenolpyruvate + erythrose-4P => chorismate	100	0	100	0	0	100	0	0	100	100	100	100	100	100	100	100	0	0	100	0
M00023	Tryptophan biosynthesis, chorismate => tryptophan	100	0	100	0	0	100	0	0	100	100	100	100	100	100	100	100	0	0	100	0
M00024	Phenylalanine biosynthesis, chorismate => phenylalanine	0	0	66.7	0	0	50	0	0	66.7	66.7	33.3	66.7	33.3	33.3	33.3	33.3	0	0	0	0
M00025	Tyrosine biosynthesis, chorismate => tyrosine	33.3	0	50	0	0	33.3	0	0	50	50	50	50	33.3	0	33.3	0	0	0	33.3	0
M00042	Catecholamine biosynthesis, tyrosine => dopamine => noradrenaline => adrenaline	0	25	0	25	25	0	25	25	0	0	0	0	0	0	0	0	0	25	0	0
M00044	Tyrosine degradation, tyrosine => homogentisate	0	0	0	0	0	0	0	0	0	0	0	0	0	0	0	0	0	0	0	0
M00038	Tryptophan metabolism, tryptophan => kynurenine => 2-aminomuconate	0	0	0	0	0	0	0	0	0	0	0	0	0	0	0	0	0	0	0	0
M00027	GABA (gamma-Aminobutyrate) shunt	66.7	33.3	0	33.3	33.3	33.3	33.3	33.3	0	33.3	33.3	0	33.3	33.3	33.3	33.3	33.3	33.3	33.3	33.3
M00127	Thiamine biosynthesis, AIR => thiamine-P/thiamine-2P	40	40	60	40	80	60	60	60	60	60	60	60	60	80	60	100	20	40	40	40
M00125	Riboflavin biosynthesis, GTP => riboflavin/FMN/FAD	50	50	50	0	0	16.7	0	50	50	50	50	50	50	0	50	50	0	50	50	0
M00124	Pyridoxal biosynthesis, erythrose-4P => pyridoxal-5P	0	0	0	0	0	16.7	0	0	0	0	0	0	0	16.7	0	0	0	0	0	0
M00115	NAD biosynthesis, aspartate => NAD	20	20	60	20	20	20	20	60	60	80	20	80	20	80	80	80	40	20	20	20
M00622	Nicotinate degradation, nicotinate => fumarate	16.7	0	0	0	0	0	0	0	0	0	0	16.7	0	16.7	0	16.7	0	16.7	0	0
M00119	Pantothenate biosynthesis, valine/L-aspartate => pantothenate	40	40	20	40	40	20	40	40	40	20	20	20	60	40	80	60	0	40	40	40

ID	Module name	Module completion ratio (%)																			
		ape	smr	iho	dmu	tag	iag	thg	hbu	pfm	sai	mse	aho	pis	cma	ttn	vdi	tpe	asc	clg	ffo
M00120	Coenzyme A biosynthesis, pantothenate => CoA	33.3	33.3	33.3	33.3	33.3	33.3	33.3	33.3	33.3	33.3	33.3	33.3	50	50	50	50	25	33.3	33.3	33.3
M00572	Pimeloyl-ACP biosynthesis, BioC-BioH pathway, malonyl-ACP => pimeloyl-ACP	16.7	16.7	16.7	16.7	0	0	0	16.7	16.7	16.7	16.7	16.7	16.7	16.7	16.7	16.7	16.7	16.7	16.7	16.7
M00123	Biotin biosynthesis, pimeloyl-ACP/CoA => biotin	0	25	0	25	25	0	0	25	25	25	25	25	0	0	0	0	0	0	0	0
M00573	Biotin biosynthesis, BioI pathway, long-chain-acyl-ACP => pimeloyl-ACP => biotin	0	20	0	20	20	0	0	20	20	20	20	20	0	0	0	0	0	0	0	0
M00577	Biotin biosynthesis, BioW pathway, pimelate => pimeloyl-CoA => biotin	0	20	0	20	20	0	0	20	20	20	20	20	0	0	0	0	0	0	0	0
M00126	Tetrahydrofolate biosynthesis, GTP => THF	0	0	20	0	0	0	16.7	20	20	20	20	20	33.3	33.3	33.3	33.3	0	0	0	0
M00121	Heme biosynthesis, glutamate => protoheme/siroheme	54.5	9.1	54.5	9.1	9.1	27.3	18.2	54.5	54.5	54.5	54.5	54.5	54.5	54.5	45.5	54.5	9.1	54.5	54.5	9.1
M00129	Ascorbate biosynthesis, animals, glucose-1P => ascorbate	0	0	0	0	0	16.7	0	0	0	0	33.3	0	33.3	16.7	0	0	33.3	0	0	0
M00114	Ascorbate biosynthesis, plants, glucose-6P => ascorbate	37.5	37.5	22.2	37.5	37.5	37.5	37.5	25	25	37.5	37.5	37.5	37.5	37.5	37.5	25	25	37.5	37.5	37.5
M00550	Ascorbate degradation, ascorbate => D-xylulose-5P	0	0	0	0	0	0	0	0	0	0	0	0	0	0	0	0	0	0	0	0
M00122	Cobalamin biosynthesis, cobinamide => cobalamin	37.5	0	0	0	0	25	0	0	25	37.5	37.5	50	37.5	50	37.5	50	0	25	25	25
M00117	Ubiquinone biosynthesis, prokaryotes, chorismate => ubiquinone	22.2	11.1	22.2	11.1	11.1	11.1	11.1	33.3	33.3	33.3	33.3	33.3	33.3	22.2	33.3	22.2	22.2	22.2	22.2	33.3
M00116	Menaquinone biosynthesis, chorismate => menaquinone	11.1	11.1	11.1	0	0	0	0	22.2	11.1	11.1	22.2	11.1	11.1	22.2	11.1	22.2	33.3	33.3	33.3	22.2
M00118	Glutathione biosynthesis, glutamate => glutathione	0	0	0	0	0	0	0	0	0	0	0	0	0	0	0	0	0	0	0	0
M00140	C1-unit interconversion, prokaryotes	33.3	33.3	33.3	33.3	33.3	33.3	33.3	33.3	33.3	33.3	33.3	33.3	66.7	33.3	66.7	33.3	0	33.3	33.3	33.3
M00141	C1-unit interconversion, eukaryotes	50	50	50	50	50	50	50	50	50	50	50	50	50	50	50	50	0	50	50	50
M00133	Polyamine biosynthesis, arginine => agmatine => putrescine => spermidine	75	100	75	100	100	75	100	75	75	75	75	75	75	75	75	75	100	75	75	75
M00134	Polyamine biosynthesis, arginine => ornithine => putrescine	0	0	0	0	0	0	0	0	0	0	0	0	0	0	0	0	0	0	0	0
M00135	GABA biosynthesis, eukaryotes, putrescine => GABA	0	0	0	0	0	0	0	0	0	33.3	0	0	0	33.3	0	33.3	0	0	0	0
M00039	Lignin biosynthesis, cinnamate => lignin	0	0	0	0	0	0	0	0	0	0	0	0	0	0	0	0	0	0	0	0
M00033	Ectoine biosynthesis, aspartate => ectoine	40	0	40	0	0	40	40	20	40	40	40	40	40	40	40	60	0	0	40	0
Secondary metabolism																					
M00569	Catechol meta-cleavage, catechol => pyruvate + acetaldehyde	0	0	0	0	16.7	16.7	0	0	0	16.7	16.7	0	0	0	0	0	0	0	0	0
M00541	Benzoyl-CoA degradation, benzoyl-CoA => 3-hydroxypimeloyl-CoA	0	0	0	0	0	0	0	0	0	0	0	0	0	0	0	0	0	0	0	0
M00540	Benzoate degradation, cyclohexanecarboxylic acid => pimeloyl-CoA	0	0	0	0	0	0	0	0	0	0	0	0	0	0	0	0	0	0	0	0
M00623	Phthalate degradation, phthalate => protocatechuate	0	0	0	0	0	0	0	0	0	0	0	0	0	0	0	0	0	0	0	0

ID	Module name	Module completion ratio (%)																			
		ape	smr	iho	dmu	tag	iag	thg	hbu	pfm	sai	mse	aho	pis	cma	ttn	vdi	tpe	asc	clg	ffo
Structural complex																					
Energy metabolism																					
M00163	Photosystem I	0	0	16.7	0	0	0	0	0	0	0	0	0	0	0	0	0	0	0	0	0
M00144	NADH:quinone oxidoreductase, prokaryotes	78.6	38.5	76.9	38.5	42.9	23.1	42.9	71.4	78.6	76.9	76.9	76.9	71.4	71.4	71.4	71.4	78.6	78.6	71.4	64.3
M00145	NAD(P)H:quinone oxidoreductase, chloroplasts and cyanobacteria	0	0	0	0	0	0	0	9.1	0	0	0	0	9.1	0	0	0	9.1	0	0	0
M00149	Succinate dehydrogenase, prokaryotes	100	0	75	0	0	0	0	100	50	75	75	100	75	100	75	100	75	100	100	0
M00150	Fumarate reductase, prokaryotes	0	0	0	0	0	0	0	0	0	0	0	0	0	0	0	0	0	0	0	0
M00162	Cytochrome b6f complex	0	0	0	0	0	0	0	0	0	0	0	0	0	0	0	0	0	0	0	0
M00151	Cytochrome bc1 complex respiratory unit	0	0	0	0	0	0	0	0	0	0	0	0	0	0	0	0	0	0	0	0
M00152	Cytochrome bc1 complex	0	0	0	0	0	0	0	0	0	0	0	0	0	0	0	0	0	0	0	0
M00154	Cytochrome c oxidase	0	0	0	0	0	0	0	0	0	0	0	0	0	0	0	0	0	0	0	0
M00155	Cytochrome c oxidase, prokaryotes	100	0	0	0	0	0	0	0	0	0	66.7	0	0	66.7	0	0	0	0	0	0
M00153	Cytochrome d ubiquinol oxidase	0	0	0	0	0	0	0	50	0	0	0	50	50	50	50	50	50	50	50	0
M00416	Cytochrome aa3-600 menaquinol oxidase	0	0	0	0	0	0	0	0	0	75	75	50	0	0	0	0	0	0	0	0
M00157	F-type ATPase, prokaryotes and chloroplasts	0	0	0	0	0	0	0	0	0	0	0	0	0	0	0	0	0	0	0	0
M00159	V-type ATPase, prokaryotes	87.5	75	87.5	75	75	87.5	87.5	87.5	87.5	87.5	87.5	87.5	100	100	100	100	75	87.5	100	100
Carbohydrate & lipid metabolism																					
M00310	Pyruvate:ferredoxin oxidoreductase	0	100	100	100	100	100	100	100	100	100	100	100	100	100	100	100	100	100	100	100
M00311	2-oxoglutarate:ferredoxin oxidoreductase	50	50	100	100	100	50	100	25	100	50	50	50	50	50	50	100	50	50	50	50
M00312	2-oxoisovalerate:ferredoxin oxidoreductase	0	0	0	0	0	0	0	0	0	0	0	0	0	0	0	0	0	0	0	0
M00313	indolepyruvate:ferredoxin oxidoreductase	0	100	0	100	100	0	100	100	0	100	100	0	100	100	100	100	100	100	100	100
M00072	Oligosaccharyltransferase	0	16.7	16.7	16.7	16.7	16.7	16.7	16.7	16.7	16.7	16.7	16.7	16.7	16.7	16.7	16.7	16.7	16.7	16.7	16.7
Genetic information processing																					
M00260	DNA polymerase III complex, bacteria	0	0	0	0	0	0	0	0	0	0	0	0	0	0	0	0	0	0	0	0
M00264	DNA polymerase II complex, archaea	0	0	0	0	0	0	0	0	0	0	0	0	0	0	0	0	0	0	0	0
M00261	DNA polymerase alpha / primase complex	25	25	25	25	25	25	25	25	25	25	25	25	25	25	25	25	25	25	25	25
M00288	RPA complex	33.3	33.3	33.3	33.3	33.3	33.3	33.3	33.3	0	33.3	33.3	33.3	0	0	0	0	33.3	33.3	33.3	33.3
M00290	Holo-TFIIH complex	10	0	10	0	0	10	0	10	10	10	10	10	10	10	10	10	10	0	10	0

ID	Module name	Module completion ratio (%)																			
		ape	smr	iho	dmu	tag	iag	thg	hbu	pfm	sai	mse	aho	pis	cma	ttn	vdi	tpe	asc	clg	ffo
M00413	FA core complex	0	0	0	0	0	0	0	0	0	0	0	0	0	0	0	0	0	0	0	0
M00184	RNA polymerase, archaea	100	100	100	100	100	100	100	100	100	100	100	91.7	100	100	100	100	91.7	100	91.7	100
M00180	RNA polymerase II, eukaryotes	0	0	0	0	0	0	0	0	0	0	0	0	0	0	0	0	8.3	0	0	0
M00181	RNA polymerase III, eukaryotes	0	0	0	0	0	0	0	0	0	0	0	0	0	0	0	0	6.2	0	0	0
M00182	RNA polymerase I, eukaryotes	0	0	0	0	0	0	0	0	0	0	0	0	0	0	0	0	8.3	0	0	0
M00425	H/ACA ribonucleoprotein complex	50	25	50	25	25	25	50	50	50	50	50	50	50	50	50	50	50	25	25	50
M00390	Exosome, archaea	100	100	100	100	100	100	100	100	100	100	100	100	100	100	100	100	100	100	100	100
M00391	Exosome, eukaryotes	40	40	40	40	40	40	40	40	40	40	40	40	40	40	40	40	40	40	40	40
M00392	Ski complex	0	0	0	0	0	0	0	0	0	0	0	0	0	0	0	0	0	0	0	0
M00394	RNA degradosome	25	25	25	25	25	25	25	25	25	25	25	25	25	25	25	25	25	25	25	25
M00178	Ribosome, bacteria	59.6	59.6	59.6	59.6	59.6	59.6	59.6	59.6	59.6	59.6	59.6	59.6	59.6	57.7	59.6	59.6	59.6	59.6	59.6	59.6
M00179	Ribosome, archaea	100	98.3	100	100	100	100	100	100	100	100	100	100	100	98.3	100	100	100	98.3	100	100
M00177	Ribosome, eukaryotes	41.8	41.8	41.8	39.2	39.2	41.8	39.2	41.8	41.8	39.2	39.2	40.5	40.5	39.2	40.5	41.8	41.8	36.7	38	39.2
M00341	Proteasome, 19S regulatory particle (PA700)	0	0	0	0	0	0	0	0	0	0	0	0	0	0	0	0	0	0	0	0
M00342	Bacterial proteasome	50	50	50	50	50	50	50	50	50	50	50	50	50	50	50	50	50	50	50	50
M00343	Archaeal proteasome	100	100	100	100	100	100	100	100	100	100	100	100	66.7	66.7	66.7	66.7	66.7	100	100	100
M00401	Sec61 complex	33.3	0	0	33.3	0	0	0	0	33.3	0	0	0	0	0	0	0	0	33.3	33.3	33.3
Environmental information processing																					
M00189	Molybdate transport system	66.7	0	100	100	100	0	100	100	100	0	0	0	0	0	0	0	0	0	0	0
M00423	Molybdate/tungstate transport system	0	100	0	0	0	0	0	0	0	100	100	100	100	100	100	100	0	100	100	100
M00186	Tungstate transport system	0	0	100	0	0	100	100	100	100	0	0	0	100	0	0	0	100	0	0	0
M00188	NitT/TauT family transport system	0	66.7	0	66.7	0	33.3	66.7	100	0	66.7	66.7	66.7	100	66.7	66.7	66.7	66.7	100	66.7	100
M00190	Iron(III) transport system	0	0	0	0	0	0	0	0	0	0	0	0	0	0	0	0	100	0	0	0
M00191	Thiamine transport system	0	0	0	0	0	0	0	66.7	0	0	0	0	100	0	0	0	0	0	0	0
M00299	Spermidine/putrescine transport system	100	0	0	0	0	0	0	0	0	0	0	0	100	0	0	0	100	0	0	0
M00193	Putative spermidine/putrescine transport system	0	0	0	0	0	0	0	0	0	0	0	0	0	0	0	0	0	0	0	0
M00208	Glycine betaine/proline transport system	0	0	0	0	0	0	0	0	0	0	0	0	0	0	0	0	0	0	0	0
M00209	Osmoprotectant transport system	0	0	0	0	0	0	0	0	0	0	0	0	0	0	0	0	0	0	0	0

ID	Module name	Module completion ratio (%)																			
		ape	smr	iho	dmu	tag	iag	thg	hbu	pfm	sai	mse	aho	pis	cma	ttn	vdi	tpe	asc	clg	ffo
M00194	Maltose/maltodextrin transport system	25	25	0	25	25	25	25	25	0	25	25	25	0	0	25	0	25	25	25	25
M00196	Multiple sugar transport system	25	25	0	25	25	25	25	25	0	25	25	25	0	0	25	0	25	25	25	25
M00203	Glucose/arabinose transport system	0	0	0	0	0	0	0	0	0	0	0	0	0	100	100	0	100	100	100	0
M00605	Glucose/mannose transport system	25	100	0	25	25	25	25	25	0	25	25	25	0	0	25	0	25	25	25	25
M00604	Trehalose transport system	0	0	0	0	0	0	0	0	0	0	100	0	0	0	0	0	0	0	0	0
M00206	Cellobiose transport system	25	25	0	25	25	25	25	25	0	25	25	25	0	0	25	0	25	25	25	25
M00606	N,N'-Diacetylchitobiose transport system	25	25	0	25	25	25	25	25	0	25	25	25	0	0	25	0	25	25	25	25
M00212	Ribose transport system	0	0	0	0	0	100	100	0	0	0	0	0	0	0	0	0	0	0	0	0
M00599	Inositol-phosphate transport system	100	100	0	0	0	0	0	100	100	0	0	0	0	0	0	0	75	0	0	0
M00197	Putative fructooligosaccharide transport system	25	25	0	25	25	25	25	25	0	25	25	25	0	0	25	0	25	25	25	25
M00198	Putative sn-glycerol-phosphate transport system	0	0	0	0	0	0	0	0	0	0	0	0	0	0	0	0	0	0	0	0
M00200	Putative sorbitol/mannitol transport system	25	25	0	25	25	25	25	25	0	25	25	25	0	0	25	0	25	25	25	25
M00602	Arabinosaccharide transport system	25	25	0	25	25	25	25	25	0	25	25	25	0	0	25	0	25	25	25	25
M00491	arabinogalactan oligomer/maltooligosaccharide transport system	25	25	0	100	75	100	100	25	0	100	25	100	75	0	100	75	100	100	25	25
M00207	Putative multiple sugar transport system	100	100	0	100	100	25	25	100	0	25	100	50	0	0	50	75	100	100	50	50
M00221	Putative simple sugar transport system	100	100	0	100	100	100	100	100	100	66.7	0	0	100	0	66.7	0	100	0	0	0
M00211	Putative ABC transport system (Saccharide and polyol_2)	0	0	0	0	0	0	0	0	0	0	0	0	0	0	0	0	0	0	0	0
M00222	Phosphate transport system	100	100	100	100	0	100	100	100	100	0	0	0	100	0	0	0	100	0	0	100
M00223	Phosphonate transport system	0	0	0	0	0	0	0	0	0	0	0	0	0	0	0	0	0	0	0	0
M00237	Branched-chain amino acid transport system	100	0	100	0	0	100	100	60	100	0	0	0	100	0	100	0	100	100	100	0
M00236	Putative polar amino acid transport system	100	66.7	0	0	0	100	0	100	0	0	0	0	100	0	0	0	100	0	0	0
M00439	Oligopeptide transport system	0	0	0	0	0	0	0	0	0	0	0	0	0	0	0	0	0	0	0	0
M00239	Peptides/nickel transport system	100	100	100	80	100	100	80	100	100	80	100	80	100	100	60	60	100	60	60	60
M00240	Iron complex transport system	100	100	66.7	100	100	100	100	66.7	100	100	100	100	100	100	100	100	100	100	100	100
M00242	Zinc transport system	0	0	0	0	0	0	0	0	0	0	0	0	0	0	0	0	0	0	0	0
M00318	Iron/zinc/copper transport system	0	0	0	0	0	0	0	0	0	0	0	0	0	0	0	0	0	0	0	0
M00317	Manganese/iron transport system	0	0	0	0	0	0	0	0	0	0	0	0	0	0	0	0	0	0	0	0
M00319	Manganese/zinc/iron transport system	0	0	0	0	0	0	0	0	0	0	0	0	0	0	0	0	0	0	0	0

ID	Module name	Module completion ratio (%)																			
		ape	smr	iho	dmu	tag	iag	thg	hbu	pfm	sai	mse	aho	pis	cma	ttn	vdi	tpe	asc	clg	ffo
M00243	Manganese/iron transport system	0	0	0	0	0	0	0	0	0	0	0	0	0	0	0	0	0	0	0	0
M00245	Cobalt/nickel transport system	0	100	0	0	0	100	75	100	100	0	0	0	100	0	100	0	100	0	0	100
M00246	Nickel transport system	0	100	0	0	0	100	66.7	100	100	0	0	0	100	0	100	0	100	0	0	100
M00581	Biotin transport system	0	0	0	33.3	0	33.3	33.3	0	0	0	0	0	0	0	0	0	33.3	0	0	0
M00582	Energy-coupling factor transport system	75	100	0	100	100	100	100	0	0	75	75	75	0	50	75	0	100	0	0	100
M00244	Putative zinc/manganese transport system	100	100	100	100	100	100	100	100	66.7	0	0	0	100	0	100	0	100	0	0	100
M00298	Multidrug/hemolysin transport system	0	0	0	0	0	0	0	0	0	0	0	0	0	0	0	0	0	0	0	0
M00250	Lipopolysaccharide transport system	0	0	0	0	0	0	0	0	0	0	0	0	0	0	0	0	0	0	0	0
M00320	Lipopolysaccharide export system	0	0	0	0	0	0	0	0	0	0	0	0	0	0	0	0	0	0	0	0
M00252	Lipooligosaccharide transport system	0	0	0	0	0	0	0	0	0	0	0	0	0	0	0	0	0	0	0	0
M00224	Fluoroquinolones transport system	0	0	0	0	0	0	0	0	0	0	0	0	0	0	0	0	0	0	0	0
M00255	Lipoprotein-releasing system	0	0	0	0	0	0	0	0	0	0	0	0	0	0	0	0	0	0	0	0
M00259	Heme transport system	0	0	0	0	0	0	0	0	66.7	0	0	0	0	0	0	0	0	0	0	0
M00258	Uncharacterized ABC transport system_2	50	100	50	100	100	100	100	100	100	0	100	0	100	100	100	100	100	0	0	100
M00254	Uncharacterized ABC transport system_3	100	100	100	100	100	100	100	100	100	100	100	100	100	100	100	100	100	100	100	100
M00273	PTS system, fructose-specific II component	0	0	0	0	0	0	0	0	0	0	0	0	0	0	0	0	0	0	0	0
M00279	PTS system, galactitol-specific II component	0	0	0	0	0	0	0	0	0	0	0	0	0	0	0	0	0	0	0	0
M00276	PTS system, mannose-specific II component	0	0	0	0	0	0	0	0	0	0	0	0	0	0	0	0	75	0	0	0
M00334	Type VI secretion system	0	0	0	0	0	0	0	0	0	0	0	0	0	0	0	0	0	0	0	0
M00335	Sec (secretion) system	25	25	25	25	25	25	25	25	25	25	25	25	25	25	25	25	25	25	25	25

ID	Module name	Module completion ratio (%)																			
		ape	smr	iho	dmu	tag	iag	thg	hbu	pfm	sai	mse	aho	pis	cma	ttn	vdi	tpe	asc	clg	ffo
Functional set																					
Metabolism																					
M00360	Aminoacyl-tRNA biosynthesis, prokaryotes	100	100	100	100	100	100	100	100	100	100	100	100	100	100	94.4	100	100	100	100	100
M00359	Aminoacyl-tRNA biosynthesis, eukaryotes	85	90	85	90	90	90	95	85	85	90	90	90	95	95	90	90	90	95	95	95
M00362	Nucleotide sugar biosynthesis, prokaryotes	28.6	57.1	28.6	42.9	42.9	57.1	42.9	14.3	14.3	42.9	71.4	42.9	57.1	42.9	28.6	42.9	85.7	57.1	57.1	42.9
M00361	Nucleotide sugar biosynthesis, eukaryotes	16.7	33.3	16.7	33.3	33.3	33.3	33.3	0	0	33.3	66.7	33.3	66.7	33.3	33.3	33.3	66.7	33.3	33.3	33.3
Environmental information processing																					
M00454	KdpD-KdpE (potassium transport) two-component regulatory system	0	0	0	0	0	0	0	0	0	0	0	0	0	0	0	0	0	0	0	0
M00459	VicK-VicR (cell wall metabolism) two-component regulatory system	0	0	0	0	0	0	0	0	0	0	0	0	0	0	0	0	0	0	0	0
M00460	MprB-MprA (maintenance of persistent infection two-component regulatory system	0	0	0	0	0	0	0	0	0	0	0	0	0	0	0	0	0	0	0	0
M00506	CheA-CheYBV (chemotaxis) two-component regulatory system	0	0	0	0	0	0	0	0	0	0	0	0	0	0	0	0	0	0	0	0
M00507	ChpA-ChpB/PilGH (chemosensory) two-component regulatory system	0	0	0	0	0	0	0	0	0	0	0	0	0	0	0	0	0	0	0	0
M00511	PleC-PleD (cell fate control) two-component regulatory system	0	0	0	0	0	0	0	0	0	0	0	0	0	0	0	0	0	0	0	0
M00520	ChvG-ChvI (acidity sensing) two-component regulatory system	0	0	0	0	0	0	0	0	0	0	0	0	0	0	0	0	0	0	0	0

Full species name of each abbreviation is described in Table S2.

Table S3 Module completion patterns in 79 archaeal species listed in Table S2 (continied: Thaumarchaeota and other archaea).

ID	Module name	Module completion ratio (%)					
		nmr	csy	nga	csu	neq	kcr
Pathway module							
Energy metabolism							
M00165	Reductive pentose phosphate cycle (Calvin cycle)	45.5	45.5	45.5	27.3	0	27.3
M00166	Reductive pentose phosphate cycle, ribulose-5P => glyceraldehyde-3P	50	50	50	50	0	50
M00167	Reductive pentose phosphate cycle, glyceraldehyde-3P => ribulose-5P	42.9	42.9	42.9	14.3	0	14.3
M00168	CAM (Crassulacean acid metabolism), dark	50	50	50	100	0	0
M00169	CAM (Crassulacean acid metabolism), light	50	50	50	50	0	50
M00172	C4-dicarboxylic acid cycle, NADP - malic enzyme type	25	25	25	50	0	25
M00171	C4-dicarboxylic acid cycle, NAD - malic enzyme type	14.3	14.3	14.3	28.6	0	14.3
M00170	C4-dicarboxylic acid cycle, phosphoenolpyruvate carboxykinase type	25	25	25	25	0	25
M00173	Reductive citrate cycle (Arnon-Buchanan cycle)	63.6	63.6	63.6	90.9	9.1	54.5
M00376	3-Hydroxypropionate bi-cycle	18.8	18.8	18.8	12.5	0	12.5
M00375	Hydroxypropionate-hydroxybutylate cycle	41.7	41.7	41.7	16.7	0	16.7
M00374	Dicarboxylate-hydroxybutyrate cycle	46.2	46.2	46.2	61.5	7.7	46.2
M00377	Reductive acetyl-CoA pathway (Wood-Ljungdahl pathway)	14.3	14.3	14.3	0	0	14.3
M00579	Phosphate acetyltransferase-acetate kinase pathway, acetyl-CoA => acetate	0	0	0	0	0	0
M00175	Nitrogen fixation, nitrogen => ammonia	0	0	0	0	0	0
M00531	Assimilatory nitrate reduction, nitrate => ammonia	0	50	0	0	0	0
M00530	Dissimilatory nitrate reduction, nitrate => ammonia	0	0	0	0	0	0
M00529	Denitrification, nitrate => nitrogen	25	0	25	25	0	0
M00528	Nitrification, ammonia => nitrite	50	50	50	0	0	0
M00567	Methanogenesis, CO2 => methane	0	0	0	12.5	0	12.5
M00357	Methanogenesis, acetate => methane	20	0	20	20	0	20
M00356	Methanogenesis, methanol => methane	0	0	0	0	0	33.3
M00563	Methanogenesis, mono-/di-/tri-methylamine => methane	0	0	0	0	0	25
M00358	Coenzyme M biosynthesis	0	0	0	0	0	0
M00174	Methane oxidation, methanotroph, methane => formaldehyde	50	50	50	0	0	0
M00346	Formaldehyde assimilation, serine pathway	33.3	33.3	33.3	44.4	0	33.3

ID	Module name	Module completion ratio (%)					
		nmr	csy	nga	csu	neq	kcr
M00345	Formaldehyde assimilation, ribulose monophosphate pathway	0	0	0	0	0	33.3
M00344	Formaldehyde assimilation, xylulose monophosphate pathway	0	0	0	0	0	0
M00378	F420 biosynthesis	60	60	60	60	0	20
M00422	Acetyl-CoA pathway, CO2 => acetyl-CoA	0	0	0	0	0	0
M00176	Assimilatory sulfate reduction, sulfate => H2S	100	100	100	0	0	33.3
M00596	Dissimilatory sulfate reduction, sulfate => H2S	33.3	33.3	33.3	0	0	0
Carbohydrate & lipid metabolism							
M00001	Glycolysis (Embden-Meyerhof pathway), glucose => pyruvate	70	70	60	90	0	80
M00002	Glycolysis, core module involving three-carbon compounds	83.3	83.3	83.3	100	0	100
M00003	Gluconeogenesis, oxaloacetate => fructose-6P	100	100	100	85.7	0	100
M00307	Pyruvate oxidation, pyruvate => acetyl-CoA	0	0	0	100	0	100
M00009	Citrate cycle (TCA cycle, Krebs cycle)	75	75	75	87.5	0	62.5
M00010	Citrate cycle, first carbon oxidation, oxaloacetate => 2-oxoglutarate	100	100	100	100	0	33.3
M00011	Citrate cycle, second carbon oxidation, 2-oxoglutarate => oxaloacetate	60	60	60	80	0	80
M00004	Pentose phosphate pathway (Pentose phosphate cycle)	57.1	57.1	57.1	42.9	0	28.6
M00006	Pentose phosphate pathway, oxidative phase, glucose 6P => ribulose 5P	0	0	50	50	0	0
M00007	Pentose phosphate pathway, non-oxidative phase,fructose 6P => ribose 5P	75	75	75	25	0	25
M00580	Pentose phosphate pathway, archaea, fructose 6P => ribose 5P	50	50	50	50	0	100
M00005	PRPP biosynthesis, ribose 5P => PRPP	100	100	100	100	0	100
M00008	Entner-Doudoroff pathway, glucose-6P => glyceraldehyde-3P + pyruvate	0	0	0	0	0	0
M00308	Semi-phosphorylative Entner-Doudoroff pathway, gluconate => glyceraldehyde-3P + pyruvate	20	20	20	25	0	20
M00633	Semi-phosphorylative Entner-Doudoroff pathway, gluconate/galactonate => glycerate-3P	0	0	0	50	0	0
M00309	Non-phosphorylative Entner-Doudoroff pathway, gluconate => glyceraldehyde + pyruvate	0	0	0	33.3	0	33.3
M00012	Glyoxylate cycle	60	60	60	100	0	0
M00373	Ethylmalonyl pathway	21.4	21.4	21.4	7.1	0	7.1
M00532	Photorespiration	10	10	10	20	10	20
M00013	Malonate semialdehyde pathway, propanoyl-CoA => Acetyl-CoA	0	0	0	25	0	50
M00632	Galactose degradation, Leloir pathway, galactose => alpha-D-glucose-1P	50	50	50	25	0	25

ID	Module name	Module completion ratio (%)					
		nmr	csy	nga	csu	neq	kcr
M00552	D-galactonate degradation, De Ley-Doudoroff pathway, D-galactonate => glycerate-3P	20	20	20	20	0	20
M00014	Glucuronate pathway (uronate pathway)	0	0	0	28.6	0	0
M00631	D-Galacturonate degradation (bacteria), D-galacturonate => pyruvate + D-glyceraldehyde 3P	0	0	0	0	0	0
M00061	Uronic acid metabolism	0	0	0	0	0	0
M00081	Pectin degradation	0	0	0	0	0	0
M00082	Fatty acid biosynthesis, initiation	0	0	0	0	0	0
M00113	Jasmonic acid biosynthesis	0	0	0	0	0	0
M00086	beta-Oxidation, acyl-CoA synthesis	100	0	0	100	0	0
M00087	beta-Oxidation	0	0	0	0	0	33.3
M00088	Ketone body biosynthesis, acetyl-CoA => acetoacetate/3-hydroxybutyrate/acetone	40	40	40	40	0	40
M00093	Phosphatidylethanolamine (PE) biosynthesis, PA => PS => PE	0	0	0	0	0	0
M00131	Inositol phosphate metabolism, Ins(1,3,4,5)P4 => Ins(1,3,4)P3 => myo-inositol	25	25	25	0	0	25
M00094	Ceramide biosynthesis	0	0	0	0	0	0
M00099	Sphingosine biosynthesis	0	0	0	0	0	0
M00060	Lipopolysaccharide biosynthesis, KDO2-lipid A	0	0	0	0	0	0
M00064	ADP-L-glycero-D-manno-heptose biosynthesis	0	0	0	0	0	0
M00080	Lipopolysaccharide biosynthesis, inner core => outer core => O-antigen	0	0	0	0	0	0
M00549	Nucleotide sugar biosynthesis, glucose => UDP-glucose	33.3	33.3	33.3	66.7	0	0
M00554	Nucleotide sugar biosynthesis, galactose => UDP-galactose	50	50	50	0	0	0
M00565	Trehalose biosynthesis, D-glucose 1P => trehalose	0	0	0	0	0	0
M00055	N-glycan precursor biosynthesis	7.7	7.7	7.7	7.7	7.7	7.7
M00076	Dermatan sulfate degradation	0	0	0	0	0	0
M00077	Chondroitin sulfate degradation	0	0	0	0	0	0
M00078	Heparan sulfate degradation	0	0	0	0	0	0
M00095	C5 isoprenoid biosynthesis, mevalonate pathway	57.1	57.1	57.1	57.1	0	57.1
M00096	C5 isoprenoid biosynthesis, non-mevalonate pathway	12.5	12.5	12.5	12.5	0	12.5
M00364	C10-C20 isoprenoid biosynthesis, bacteria	100	100	100	100	0	100
M00365	C10-C20 isoprenoid biosynthesis, archaea	100	100	100	100	0	100

ID	Module name	Module completion ratio (%)					
		nmr	csy	nga	csu	neq	kcr
M00366	C10-C20 isoprenoid biosynthesis, plants	25	25	25	25	0	25
M00367	C10-C20 isoprenoid biosynthesis, non-plant eukaryotes	33.3	33.3	33.3	33.3	0	33.3
M00097	beta-Carotene biosynthesis, GGAP => beta-carotene	0	0	0	16.7	0	0
M00372	Abscisic acid biosynthesis, beta-carotene => abscisic acid	0	0	0	0	0	0
Nucleotide & amino acid metabolism							
M00048	Inosine monophosphate biosynthesis, PRPP + glutamine => IMP	90.9	90.9	90.9	20	0	20
M00049	Adenine ribonucleotide biosynthesis, IMP => ADP, ATP	100	100	100	100	50	100
M00050	Guanine ribonucleotide biosynthesis IMP => GDP, GTP	75	75	75	75	25	75
M00546	Purine degradation, xanthine => urea	0	0	0	0	0	0
M00051	Uridine monophosphate biosynthesis, glutamine (+ PRPP) => UMP	83.3	83.3	83.3	40	0	83.3
M00052	Pyrimidine ribonucleotide biosynthesis, UMP => UDP/UTP, CDP/CTP	100	100	100	100	33.3	100
M00053	Pyrimidine deoxyribonuleotide biosynthesis, CDP/CTP => dCDP/dCTP, dTDP/dTTP	50	50	50	50	50	62.5
M00046	Pyrimidine degradation, uracil => beta-alanine, thymine => 3-aminoisobutanoate	33.3	33.3	33.3	33.3	0	0
M00020	Serine biosynthesis, glycerate-3P => serine	66.7	66.7	66.7	33.3	0	33.3
M00018	Threonine biosynthesis, aspartate => homoserine => threonine	100	100	100	100	0	100
M00555	Betaine biosynthesis, choline => betaine	0	0	0	0	0	0
M00021	Cysteine biosynthesis, serine => cysteine	50	0	50	50	0	50
M00338	Cysteine biosynthesis, homocysteine + serine => cysteine	0	0	0	0	0	0
M00609	Cysteine biosynthesis, methionine => cysteine	16.7	16.7	16.7	16.7	0	16.7
M00017	Methionine biosynthesis, apartate => homoserine => methionine	57.1	57.1	57.1	57.1	0	71.4
M00034	Methionine salvage pathway	37.5	37.5	37.5	62.5	0	62.5
M00035	Methionine degradation	75	75	75	50	0	50
M00368	Ethylene biosynthesis, methionine => ethylene	33.3	33.3	33.3	33.3	0	33.3
M00019	Valine/isoleucine biosynthesis, pyruvate => valine / 2-oxobutanoate => isoleucine	100	100	100	75	25	75
M00535	Isoleucine biosynthesis, pyruvate => 2-oxobutanoate	66.7	66.7	66.7	66.7	0	66.7
M00570	Isoleucine biosynthesis, threonine => 2-oxobutanoate => isoleucine	100	100	100	80	20	80
M00432	Leucine biosynthesis, 2-oxoisovalerate => 2-oxoisocaproate	100	100	100	100	0	100
M00036	Leucine degradation, leucine => acetoacetate + acetyl-CoA	16.7	16.7	16.7	16.7	16.7	16.7

ID	Module name	Module completion ratio (%)					
		nmr	csy	nga	csu	neq	kcr
M00016	Lysine biosynthesis, succinyl-DAP pathway, aspartate => lysine	22.2	22.2	33.3	33.3	11.1	55.6
M00525	Lysine biosynthesis, acetyl-DAP pathway, aspartate => lysine	22.2	22.2	33.3	33.3	0	44.4
M00526	Lysine biosynthesis, DAP dehydrogenase pathway, aspartate => lysine	33.3	33.3	50	50	0	66.7
M00527	Lysine biosynthesis, DAP aminotransferase pathway, aspartate => lysine	28.6	28.6	42.9	42.9	0	57.1
M00030	Lysine biosynthesis, AAA pathway, 2-oxoglutarate => 2-aminoadipate => lysine	0	0	0	12.5	0	0
M00433	Lysine biosynthesis, 2-oxoglutarate => 2-oxoadipate	0	0	0	0	0	0
M00031	Lysine biosynthesis, 2-aminoadipate => lysine	100	100	80	100	0	100
M00032	Lysine degradation, lysine => saccharopine => acetoacetyl-CoA	0	0	0	12.5	0	0
M00608	2-oxocarboxylic acid chain extension, 2-oxoglutarate =>2-oxoadipate => 2-oxopimelate => 2-oxosuberate	0	0	0	0	0	0
M00015	Proline biosynthesis, glutamate => proline	0	0	0	0	0	50
M00028	Ornithine biosynthesis, glutamate => ornithine	60	60	40	80	0	80
M00029	Urea cycle	60	60	60	60	0	60
M00026	Histidine biosynthesis, PRPP => histidine	75	75	77.8	77.8	0	0
M00045	Histidine degradation, histidine => N-formiminoglutamate => glutamate	25	0	0	0	0	0
M00022	Shikimate pathway, phosphoenolpyruvate + erythrose-4P => chorismate	71.4	71.4	71.4	71.4	0	71.4
M00023	Tryptophan biosynthesis, chorismate => tryptophan	80	80	80	80	0	0
M00024	Phenylalanine biosynthesis, chorismate => phenylalanine	33.3	33.3	33.3	33.3	50	50
M00025	Tyrosine biosynthesis, chorismate => tyrosine	33.3	33.3	33.3	33.3	33.3	66.7
M00042	Catecholamine biosynthesis, tyrosine => dopamine => noradrenaline => adrenaline	0	0	0	0	0	0
M00044	Tyrosine degradation, tyrosine => homogentisate	0	0	0	0	0	0
M00038	Tryptophan metabolism, tryptophan => kynurenine => 2-aminomuconate	0	0	14.3	0	0	0
M00027	GABA (gamma-Aminobutyrate) shunt	33.3	0	66.7	33.3	0	33.3
M00127	Thiamine biosynthesis, AIR => thiamine-P/thiamine-2P	60	60	60	60	0	40
M00125	Riboflavin biosynthesis, GTP => riboflavin/FMN/FAD	50	50	50	0	0	16.7
M00124	Pyridoxal biosynthesis, erythrose-4P => pyridoxal-5P	0	0	0	0	0	16.7
M00115	NAD biosynthesis, aspartate => NAD	60	60	60	20	0	20
M00622	Nicotinate degradation, nicotinate => fumarate	0	0	0	16.7	0	0
M00119	Pantothenate biosynthesis, valine/L-aspartate => pantothenate	40	40	40	20	20	0

ID	Module name	Module completion ratio (%)					
		nmr	csy	nga	csu	neq	kcr
M00120	Coenzyme A biosynthesis, pantothenate => CoA	50	50	50	25	0	50
M00572	Pimeloyl-ACP biosynthesis, BioC-BioH pathway, malonyl-ACP => pimeloyl-ACP	16.7	16.7	16.7	16.7	16.7	16.7
M00123	Biotin biosynthesis, pimeloyl-ACP/CoA => biotin	75	75	75	0	0	25
M00573	Biotin biosynthesis, BioI pathway, long-chain-acyl-ACP => pimeloyl-ACP => biotin	60	60	60	0	0	20
M00577	Biotin biosynthesis, BioW pathway, pimelate => pimeloyl-CoA => biotin	60	60	60	0	0	20
M00126	Tetrahydrofolate biosynthesis, GTP => THF	33.3	33.3	33.3	0	0	16.7
M00121	Heme biosynthesis, glutamate => protoheme/siroheme	54.5	54.5	54.5	18.2	9.1	18.2
M00129	Ascorbate biosynthesis, animals, glucose-1P => ascorbate	0	0	0	33.3	0	0
M00114	Ascorbate biosynthesis, plants, glucose-6P => ascorbate	37.5	37.5	25	37.5	0	44.4
M00550	Ascorbate degradation, ascorbate => D-xylulose-5P	0	0	0	0	0	0
M00122	Cobalamin biosynthesis, cobinamide => cobalamin	50	50	50	0	0	12.5
M00117	Ubiquinone biosynthesis, prokaryotes, chorismate => ubiquinone	22.2	22.2	33.3	11.1	0	33.3
M00116	Menaquinone biosynthesis, chorismate => menaquinone	22.2	22.2	22.2	11.1	0	22.2
M00118	Glutathione biosynthesis, glutamate => glutathione	0	0	0	0	0	0
M00140	C1-unit interconversion, prokaryotes	66.7	66.7	66.7	33.3	0	33.3
M00141	C1-unit interconversion, eukaryotes	50	50	50	50	0	50
M00133	Polyamine biosynthesis, arginine => agmatine => putrescine => spermidine	50	50	50	75	25	75
M00134	Polyamine biosynthesis, arginine => ornithine => putrescine	0	0	0	0	0	50
M00135	GABA biosynthesis, eukaryotes, putrescine => GABA	0	0	0	33.3	0	0
M00039	Lignin biosynthesis, cinnamate => lignin	0	0	0	0	0	0
M00033	Ectoine biosynthesis, aspartate => ectoine	100	40	40	40	0	40
Secondary metabolism							
M00569	Catechol meta-cleavage, catechol => pyruvate + acetaldehyde	33.3	16.7	16.7	0	0	16.7
M00541	Benzoyl-CoA degradation, benzoyl-CoA => 3-hydroxypimeloyl-CoA	0	0	0	0	0	0
M00540	Benzoate degradation, cyclohexanecarboxylic acid => pimeloyl-CoA	0	0	0	0	0	0
M00623	Phthalate degradation, phthalate => protocatechuate	0	0	0	0	0	0

ID	Module name	Module completion ratio (%)					
		nmr	csy	nga	csu	neq	kcr
Structural complex							
Energy metabolism							
M00163	Photosystem I	0	0	0	0	0	0
M00144	NADH:quinone oxidoreductase, prokaryotes	64.3	71.4	71.4	57.1	0	71.4
M00145	NAD(P)H:quinone oxidoreductase, chloroplasts and cyanobacteria	0	9.1	0	0	0	0
M00149	Succinate dehydrogenase, prokaryotes	100	100	100	100	0	100
M00150	Fumarate reductase, prokaryotes	0	25	0	0	0	0
M00162	Cytochrome b6f complex	0	0	0	12.5	0	0
M00151	Cytochrome bc1 complex respiratory unit	33.3	33.3	0	0	0	0
M00152	Cytochrome bc1 complex	10	10	0	0	0	0
M00154	Cytochrome c oxidase	6.7	6.7	6.7	6.7	0	0
M00155	Cytochrome c oxidase, prokaryotes	66.7	66.7	66.7	66.7	0	0
M00153	Cytochrome d ubiquinol oxidase	0	0	0	0	0	50
M00416	Cytochrome aa3-600 menaquinol oxidase	0	0	0	25	0	0
M00157	F-type ATPase, prokaryotes and chloroplasts	0	0	0	0	0	0
M00159	V-type ATPase, prokaryotes	100	100	100	87.5	62.5	100
Carbohydrate & lipid metabolism							
M00310	Pyruvate:ferredoxin oxidoreductase	0	0	0	100	0	100
M00311	2-oxoglutarate:ferredoxin oxidoreductase	50	50	50	50	0	100
M00312	2-oxoisovalerate:ferredoxin oxidoreductase	0	0	0	0	0	0
M00313	indolepyruvate:ferredoxin oxidoreductase	0	0	0	0	0	100
M00072	Oligosaccharyltransferase	16.7	16.7	0	16.7	16.7	16.7
Genetic information processing							
M00260	DNA polymerase III complex, bacteria	0	0	0	0	0	0
M00264	DNA polymerase II complex, archaea	100	100	100	100	100	100
M00261	DNA polymerase alpha / primase complex	25	25	25	25	0	25
M00288	RPA complex	33.3	33.3	33.3	33.3	33.3	33.3
M00290	Holo-TFIIH complex	0	0	10	0	0	10

ID	Module name	Module completion ratio (%)					
		nmr	csy	nga	csu	neq	kcr
M00413	FA core complex	10	10	10	10	10	0
M00184	RNA polymerase, archaea	83.3	83.3	83.3	75	100	83.3
M00180	RNA polymerase II, eukaryotes	8.3	8.3	0	0	0	0
M00181	RNA polymerase III, eukaryotes	6.2	6.2	0	0	0	0
M00182	RNA polymerase I, eukaryotes	8.3	8.3	0	0	0	0
M00425	H/ACA ribonucleoprotein complex	50	25	50	25	50	25
M00390	Exosome, archaea	100	100	100	100	100	100
M00391	Exosome, eukaryotes	40	30	40	40	30	40
M00392	Ski complex	0	0	0	0	0	0
M00394	RNA degradosome	25	25	25	25	0	25
M00178	Ribosome, bacteria	59.6	55.8	57.7	59.6	59.6	59.6
M00179	Ribosome, archaea	94.9	88.1	91.5	94.9	94.9	96.6
M00177	Ribosome, eukaryotes	34.2	30.4	34.2	35.4	34.2	39.2
M00341	Proteasome, 19S regulatory particle (PA700)	0	0	0	5.3	0	0
M00342	Bacterial proteasome	50	50	50	50	50	50
M00343	Archaeal proteasome	66.7	66.7	66.7	66.7	100	66.7
M00401	Sec61 complex	33.3	33.3	0	0	33.3	0
Environmental information processing							
M00189	Molybdate transport system	0	0	0	0	0	0
M00423	Molybdate/tungstate transport system	0	0	0	66.7	0	100
M00186	Tungstate transport system	0	0	0	0	0	0
M00188	NitT/TauT family transport system	100	100	100	100	0	100
M00190	Iron(III) transport system	0	0	0	66.7	0	0
M00191	Thiamine transport system	0	0	0	0	0	0
M00299	Spermidine/putrescine transport system	0	0	0	75	0	0
M00193	Putative spermidine/putrescine transport system	0	0	0	0	0	0
M00208	Glycine betaine/proline transport system	0	0	0	0	0	0
M00209	Osmoprotectant transport system	0	0	0	0	0	0

ID	Module name	Module completion ratio (%)					
		nmr	csy	nga	csu	neq	kcr
M00194	Maltose/maltodextrin transport system	0	0	0	25	0	0
M00196	Multiple sugar transport system	0	0	0	25	0	0
M00203	Glucose/arabinose transport system	0	0	0	0	0	0
M00605	Glucose/mannose transport system	0	0	0	25	0	0
M00604	Trehalose transport system	0	0	0	0	0	0
M00206	Cellobiose transport system	0	0	0	25	0	0
M00606	N,N'-Diacetylchitobiose transport system	0	0	0	25	0	0
M00212	Ribose transport system	0	0	0	0	0	0
M00599	Inositol-phosphate transport system	0	0	0	0	0	0
M00197	Putative fructooligosaccharide transport system	0	0	0	25	0	0
M00198	Putative sn-glycerol-phosphate transport system	0	0	0	0	0	0
M00200	Putative sorbitol/mannitol transport system	0	0	0	25	0	0
M00602	Arabinosaccharide transport system	0	0	0	25	0	0
M00491	arabinogalactan oligomer/maltooligosaccharide transport system	0	0	0	25	0	0
M00207	Putative multiple sugar transport system	0	0	0	100	0	0
M00221	Putative simple sugar transport system	0	0	0	100	0	100
M00211	Putative ABC transport system (Saccharide and polyol_2)	0	0	0	0	0	0
M00222	Phosphate transport system	100	100	100	100	0	100
M00223	Phosphonate transport system	100	100	0	100	0	0
M00237	Branched-chain amino acid transport system	0	100	0	100	0	100
M00236	Putative polar amino acid transport system	0	0	0	0	0	100
M00439	Oligopeptide transport system	0	0	0	0	0	0
M00239	Peptides/nickel transport system	100	80	80	60	0	80
M00240	Iron complex transport system	33.3	33.3	33.3	100	0	0
M00242	Zinc transport system	100	66.7	100	100	0	0
M00318	Iron/zinc/copper transport system	0	0	0	33.3	0	0
M00317	Manganese/iron transport system	0	0	0	25	0	0
M00319	Manganese/zinc/iron transport system	100	0	0	25	0	0

ID	Module name	Module completion ratio (%)					
		nmr	csy	nga	csu	neq	kcr
M00243	Manganese/iron transport system	0	0	33.3	0	0	0
M00245	Cobalt/nickel transport system	0	0	0	0	0	100
M00246	Nickel transport system	0	0	0	0	0	100
M00581	Biotin transport system	0	0	0	0	0	0
M00582	Energy-coupling factor transport system	0	0	0	100	0	100
M00244	Putative zinc/manganese transport system	0	0	0	0	0	100
M00298	Multidrug/hemolysin transport system	0	0	0	0	0	0
M00250	Lipopolysaccharide transport system	0	0	0	0	0	0
M00320	Lipopolysaccharide export system	0	0	0	0	0	0
M00252	Lipooligosaccharide transport system	0	0	0	0	0	0
M00224	Fluoroquinolones transport system	0	0	0	0	0	0
M00255	Lipoprotein-releasing system	100	100	0	0	0	0
M00259	Heme transport system	0	0	0	0	0	0
M00258	Uncharacterized ABC transport system_2	0	0	100	100	100	100
M00254	Uncharacterized ABC transport system_3	100	100	100	100	0	100
M00273	PTS system, fructose-specific II component	0	0	0	0	0	0
M00279	PTS system, galactitol-specific II component	0	0	0	0	0	0
M00276	PTS system, mannose-specific II component	0	0	0	0	0	0
M00334	Type VI secretion system	0	0	20	0	0	0
M00335	Sec (secretion) system	25	12.5	25	25	33.3	25

ID	Module name	Module completion ratio (%)					
		nmr	csy	nga	csu	neq	kcr
Functional set							
Metabolism							
M00360	Aminoacyl-tRNA biosynthesis, prokaryotes	100	100	100	100	100	94.4
M00359	Aminoacyl-tRNA biosynthesis, eukaryotes	85	85	90	90	85	85
M00362	Nucleotide sugar biosynthesis, prokaryotes	42.9	57.1	71.4	71.4	0	57.1
M00361	Nucleotide sugar biosynthesis, eukaryotes	33.3	33.3	33.3	66.7	0	16.7
Environmental information processing							
M00454	KdpD-KdpE (potassium transport) two-component regulatory system	0	0	0	0	0	0
M00459	VicK-VicR (cell wall metabolism) two-component regulatory system	0	0	0	0	0	0
M00460	MprB-MprA (maintenance of persistent infection two-component regulatory system	0	0	0	0	0	0
M00506	CheA-CheYBV (chemotaxis) two-component regulatory system	20	0	60	0	0	0
M00507	ChpA-ChpB/PilGH (chemosensory) two-component regulatory system	0	0	0	0	0	0
M00511	PleC-PleD (cell fate control) two-component regulatory system	0	0	0	0	0	0
M00520	ChvG-ChvI (acidity sensing) two-component regulatory system	0	0	50	0	0	0

Full species name of each abbreviation is described in Table S2.
